# Adsorption Mechanisms and AI‐Driven Discovery of Biomass‐Based CO_2_ Sorbents

**DOI:** 10.1002/smll.202504877

**Published:** 2025-08-29

**Authors:** Faezeh Hajiali, Jingqian Chen, Tao Zou, Scott Renneckar, Bhushan Gopaluni, Naoko Ellis, Orlando J. Rojas

**Affiliations:** ^1^ Bioproducts Institute University of British Columbia 2385 East Mall Vancouver BC V6T 1Z4 Canada; ^2^ Department of Chemical and Biological Engineering University of British Columbia 2360 East Mall Vancouver BC V6T 1Z3 Canada; ^3^ Department of Wood Science Faculty of Forestry The University of British Columbia 2424 Main Mall #2900 Vancouver BC V6T 1Z4 Canada; ^4^ Department of Chemistry The University of British Columbia 2036 Main Mall Vancouver BC V6T 1Z1 Canada

**Keywords:** AI‐driven materials discovery, biomass‐derived activated carbon, carbon capture and storage, CO_2_ adsorption, functional groups, thermal activation

## Abstract

The pressing need to reduce carbon dioxide emissions has driven recent advances in carbon capture technologies. Among these, adsorption has emerged as one of the most efficient and promising methods for CO_2_ sequestration. This review provides a comprehensive analysis of recent progress in biomass‐derived activated carbon (AC) as a sustainable solution for carbon capture. It explores the influence of various biomass precursors, their composition, and the effects of chemical and thermal treatments on the textural properties and CO_2_ adsorption capacity of AC. The role of functional groups and pore structures in enhancing adsorption performance, particularly under humid conditions, is also examined. Additionally, the integration of artificial intelligence (AI)‐driven technologies in process modeling and the discovery of optimized bio‐based AC materials is highlighted. Classic adsorption kinetic models are reviewed to provide deeper insights into CO_2_ adsorption mechanisms and the efficiency of bio‐based AC. The discussion underscores the necessity of continued research to enhance the properties, scalability, and cost‐effectiveness of bio‐based AC while leveraging AI‐driven innovations to advance carbon capture and storage (CCS) solutions.

## Graphical abstract

This review analyzes recent advances in biomass‐derived activated carbons for CO_2_ capture. It highlights the influence of precursors, activation methods, and surface modifications on adsorption performance. The integration of AI‐driven approaches for material optimization is discussed. Challenges related to scalability, regeneration, environmental impact, and economic feasibility are also critically examined.

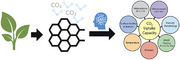

## 1 Introduction

The escalating concentration of atmospheric CO_2_, driven by fossil fuel combustion, industrial processes, and deforestation, has intensified the urgency for effective carbon mitigation strategies. Carbon capture and storage (CCS) is recognized as a critical transitional measure to achieve deep emission reductions, especially in hard‐to‐abate sectors. Among available CCS technologies, adsorption stands out for its energy efficiency, operational flexibility, and compatibility with existing infrastructure. In particular, biomass‐derived adsorbents; such as activated carbons (ACs); offer a sustainable and cost‐effective pathway, leveraging abundant, low‐cost precursors and contributing to carbon sequestration through the bioeconomy. These materials combine favorable surface chemistry, tunable porosity, and resilience under realistic flue gas conditions. This review examines the current state and future potential of biomass‐based CO_2_ adsorbents, covering feedstock selection, activation and modification strategies, performance under varying environmental conditions, and integration with Artificial Intelligence for accelerated discovery and optimization. By bridging advances in material design with emerging AI‐driven approaches, this work aims to guide the development of scalable, circular, and economically viable carbon capture solutions.

### 1.1 CO_2_ Emissions and Climate Change

Over the past 20 years, the world has witnessed a significant rise in carbon emissions into the atmosphere. This surge was driven by rapid industrialization, urbanization, increased economic development and consumption, and a soaring world population.^[^ ^1^ ^]^ The industrial revolution triggered the overconsumption of carbon‐rich compounds, such as fossil fuels, to meet growing energy demands. This consumption rate has continuously increased, with economic gains being prioritized over the environmental consequences of depleting natural resources. While nature's carbon cycle previously maintained a balance through photosynthesis, current carbon emissions have far surpassed nature's capacity to recycle the excess CO_2_. As a result, there has been a notable rise in global temperatures, leading to a consequent rise in sea levels among other extreme weather events.^[^ ^1^, ^2^, ^3^ ^]^

According to the latest data released in April 2024, the atmospheric concentration of CO_2_ has risen from 280 ppm before industrialization to a record high of 426.57 ppm.^[^ ^4^ ^]^ This is an increase of 3.2 ppm compared to April 2023, reflecting the ongoing upward trend in CO_2_ levels due to human activities such as fossil fuel combustion and deforestation. The power sector, including electricity and heat production, accounted for a major portion of global CO_2_ emissions. For instance, in 2023, global energy‐related CO_2_ emissions increased by 1.1%, with coal emissions accounting for more than 65% of this increase.^[^ ^5^ ^]^

To address this challenge, a fundamental transformation in energy generation is required to reduce fossil fuel consumption. Expanding renewable sources is crucial for decreasing reliance on fossil carbon and ultimately lowering CO_2_ emissions. However, transitioning to renewables remains challenging due to society's deep dependence on fossil fuels and the benefits they have historically provided. Therefore, active CO_2_ removal through carbon capture is essential to mitigate emissions until net‐zero energy solutions from alternative sources are fully developed.

### 1.2 Significance of Carbon Capture and Storage

The continuous rise of CO_2_ emissions highlights the importance of implementing effective carbon capture and reduction strategies to mitigate climate change impacts, as the current level significantly exceeds the natural carbon cycle's consumption capacity. The International Energy Agency (IEA) emphasizes that carbon capture is a crucial measure for reducing CO_2_ emissions. The IEA's Sustainable Development Scenario predicts that carbon capture technologies can capture and store up to 10.4 gigatons of CO_2_ by 2070, with ≈50% (around 5.2 gigatons) coming from fossil fuel use and industrial processes.^[^ ^6^ ^]^ Carbon capture technologies are particularly relevant in achieving emission reductions in sectors where decarbonization is difficult, such as long‐distance transportation, heavy industries, and power generation.^[^ ^7^ ^]^

**Figure 1:**
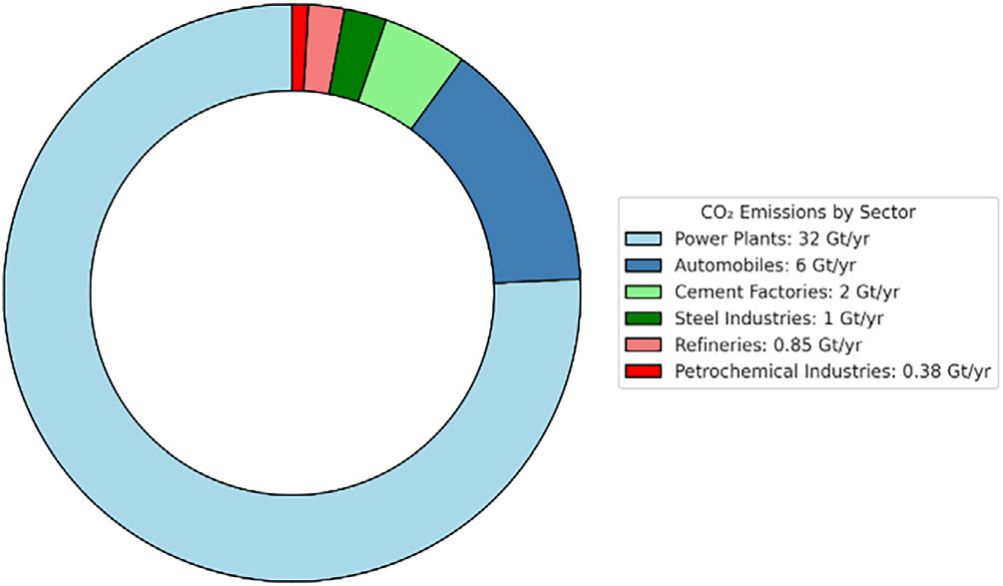
Illustration of CO_2_ emissions from various sectors (data from Ray et al.^[^ ^11^ ^]^).

CCS has emerged as a promising technology to manage CO_2_ levels in the short term. A classical example of CCS is capturing CO_2_, transporting it through pipelines to geological sites, and injecting it into geological formations for permanent storage.^[^ ^8^ ^]^ CCS can be categorized into three options: post‐combustion, pre‐combustion, and oxy‐fuel combustion. Post‐combustion involves removing CO_2_ after the combustion process has occurred. The post‐combustion carbon capture technologies can be integrated with major stationary emission sources to remove CO_2_ from flue gases, thereby reducing their greenhouse gas footprints.^[^ ^9^ ^]^ This includes steel, cement, and iron plants, petrochemical sites, refineries, and fossil fuel‐fired power plants, among others.^[^ ^10^ ^]^ **Figure** **1** illustrates CO_2_ emissions across different sectors, highlighting that power plants contribute the largest share of total emissions.^[^ ^11^ ^]^

**Figure 2:**
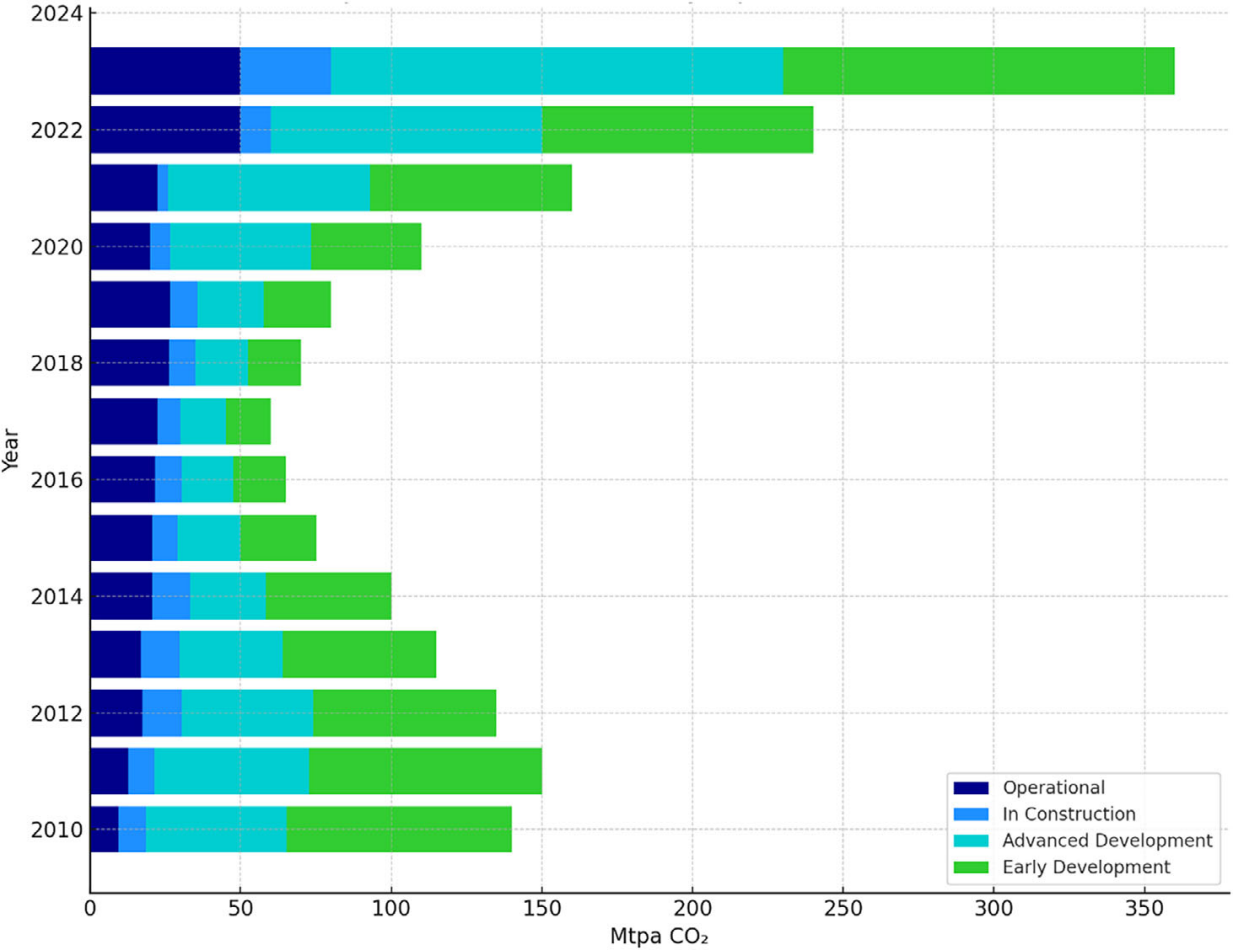
Estimated capacity of commercial CCS facilities by project status from 2010 to 2023. The figure was recreated based on data presented in the Global Status of CCS 2023 report by the Global CCS Institute.^[^ ^12^ ^]^

The recent trends in CO_2_ capture technology development further highlight the significance of advancing CCS. As shown in **Figure** **2**, the capture capacity of commercial facilities has steadily increased over the years. Since 2017, the capacity has grown at a compound annual rate of over 35%. This growth has accelerated from 2022 to 2023, with a 50% increase in capture capacity from 2022, marking the highest rise since the upward trend began in 2018. As of July 2023, there were 101 transport and/or storage projects in the deployment pipeline, either under construction or operational. Capture capacity across all project development stages has significantly increased, with a 47% rise in both Advanced Development and Early Development projects.

Post‐combustion CO_2_ capture can be easily integrated into existing plants; however, it often comes with a high energy penalty.^[^ ^13^ ^]^ Among post‐combustion capture technologies, liquid‐phase chemisorption, adsorption, and calcium looping are notable options due to their ease of retrofitting and ability to function under various temperature and pressure conditions. However, liquid‐phase chemisorption requires high energy for liquid regeneration, suffers from low thermal and oxidative stability (amine degradation), and incurs high operational costs.^[^ ^14^ ^]^ Similarly, calcium looping systems require high operating temperatures, leading to high energy consumption, sorbent degradation over multiple cycles, and increased complexity and cost of operation.^[^ ^15^ ^]^ The adsorption process, however, is more promising. There is an opportunity and growing need to enhance CO_2_ capture using adsorption by improving working capacity, selectivity, reducing water uptake, and increasing tolerance to impurities.^[^ ^16^ ^]^

#### 1.2.1 Motivations for Bio‐Based Adsorbents for Carbon Capture

**Figure 3:**
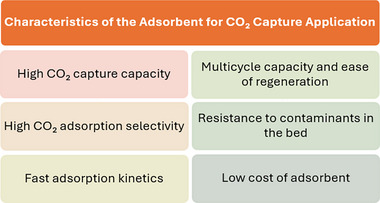
Key general characteristics of prospective materials in CCS technologies.

Solid adsorbents offer several advantages over liquid counterparts, including a wider operating temperature range up to 700 °C, lower toxicity upon disposal, and reduced waste generation.^[^ ^17^ ^]^ As shown in **Figure** **3**, an ideal adsorbent for capturing CO_2_ from combustion gases in industrial applications should possess several key characteristics.^[^ ^18^ ^]^ It should have a high CO_2_ adsorption capacity (over 7–8 mmol g^−1^ at 0 °C and 4–5 mmol g^−1^ at 25 °C) to minimize the volume of adsorbent required, which is economically advantageous.

High CO_2_ selectivity is essential for separating CO_2_ from other gases, such as nitrogen and oxygen, while maintaining effectiveness in the presence of water vapor. Durability across multiple cycles (>30) ensures longevity and reduces replacement frequency. Fast adsorption kinetics (1 min to 1 h) allow for quicker cycles and smaller installations, further optimizing cost and efficiency. The adsorbent should be regenerated easily,^[^ ^19^ ^]^ with moderate adsorbate‐adsorbent interactions to balance sorption capacity and energy use for regeneration. It must also withstand contaminants in flue gases, such as SOx, NOx, and heavy metals, to remain stable in harsh environments. Finally, low production cost (below $2–5 kg^−1^) is crucial for economic feasibility in large‐scale applications.^[^ ^20^, ^21^ ^]^

Solid sorbents can be categorized based on their operating temperatures into low, intermediate, and high‐temperature adsorbents. Low‐temperature adsorbents, which operate at temperatures below 200 °C, include carbon‐based adsorbents,^[^ ^22^ ^]^ zeolites,^[^ ^23^ ^]^ metal‐organic frameworks,^[^ ^24^ ^]^ mesoporous silica,^[^ ^25^ ^]^ and covalent organic frameworks.^[^ ^26^ ^]^ Intermediate‐temperature adsorbents, operating between 200 and 400 °C, primarily consist of hydrotalcite‐like compounds. High‐temperature adsorbents, functioning at temperatures above 400 °C, include calcium‐based and alkali ceramic‐based adsorbents.^[^ ^16^ ^]^

Among low‐temperature adsorbents, biomass‐derived porous activated carbons (ACs) are highly promising adsorbents for CO_2_ capture and gas separation. Various inexpensive biomass carbon precursors, such as wood ash,^[^ ^27^ ^]^ shrimp shell,^[^ ^28^ ^]^ rice husk,^[^ ^29^ ^]^ coconut shell,^[^ ^30^ ^]^ almond shell,^[^ ^31^ ^]^ walnut shell,^[^ ^32^ ^]^ sawdust,^[^ ^33^ ^]^ and orange peel,^[^ ^34^ ^]^ can be easily processed to prepare biomass adsorbents like AC. ACs are an excellent choice for carbon capture applications due to their customizable surface chemistry, enhanced pore structure, high separation efficiency, and cost‐effectiveness.^[^ ^35^ ^]^

**Figure 4:**
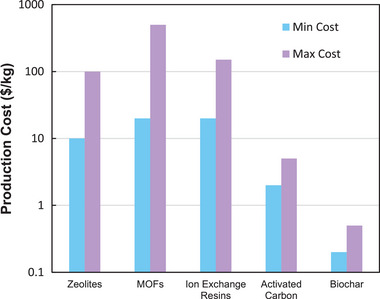
Comparison of Production Costs for five types of adsorbents.^[^ ^36^, ^37^, ^38^, ^39^ ^]^

In terms of production costs, several factors influence the price of adsorbents, including availability, collection or transportation, processing or modification, and further treatment of the adsorbent. The production costs of biobased adsorbents involve raw biomass materials and adsorbent preparations. Typically, the biomass raw material cost is relatively low or even negligible, depending on local precursor availability. For instance, CO_2_ adsorbents such as zeolites typically cost between $10 and $100 kg^−1^, depending on the type and purity.^[^ ^36^ ^]^ Metal–organic frameworks (MOFs) have a wider range of prices, often costing between $20 and $500 kg^−1^, depending on the linker type, synthesis complexity, production yield, and scale of production.^[^ ^37^, ^38^ ^]^ Ion‐exchange resins cost around $150 kg^−1^.^[^ ^39^ ^]^ In contrast, the cost of AC production is significantly lower, ranging between $2 and $5 kg^−1^.^[^ ^40^ ^]^ Biobased adsorbents like biochar, derived from biomass such as woody waste, have an even lower cost, typically ranging from $0.2 to $0.5 kg^−1^.^[^ ^39^ ^]^ This significant cost difference, along with lower lifecycle emissions and the wide availability of biomass precursors, underscores the economic feasibility and practical cost‐effectiveness of biomass‐derived ACs for large‐scale CO_2_ capture applications. **Figure** **4** illustrates the comparison between the production costs of various commercial adsorbents.^[^ ^36^, ^37^, ^38^, ^39^ ^]^

Bio‐based products are made up of ≈50% carbon by mass, playing a significant role in carbon sequestration.^[^ ^41^ ^]^ These products have the potential to capture and store carbon both above and below ground. During photosynthesis, plants absorb CO_2_, with some carbon stored in the leaves and stems above ground. A significant amount is also transferred to the roots and soil below ground. For example, switchgrass can sequester 1.5 tons of CO_2_ equivalent per hectare per year in the soil. Therefore, biomass production systems can be considered net absorbers of CO_2_, effectively functioning as carbon sinks. As a result, many biomass production systems can have negative global warming potential (GWP) values, indicating that they sequester more CO_2_ than they emit during their lifecycle. This potential needs to consider sustainable harvesting practices along with land‐use change factors.

Numerous studies have investigated the life cycle assessment of various biomass productions, including wheat straw,^[^ ^42^ ^]^ rice husk,^[^ ^43^ ^]^ bamboo,^[^ ^44^ ^]^ sugarcane bagasse,^[^ ^45^ ^]^ and northern softwood^[^ ^46^ ^]^ for different applications. From a cradle‐to‐gate perspective, the evaluated global warming potential (GWP) for biomass production ranges from 12 to 245 kg CO_2,eq_ per oven‐dry ton, considering only anthropogenic emissions. However, when accounting for potential soil organic carbon sequestration, the GWP values shift to ‐170 to 228 kg CO_2,eq_ per oven‐dry ton. This indicates the potential of biomass production to effectively function as carbon sink systems.^[^ ^47^ ^]^

Additionally, the life cycle assessment of biomass‐derived AC production reveals significant benefits. Studies show that the cradle‐to‐gate cumulative energy demand for biomass‐derived AC is nearly 35% less than that for coal‐based AC. Furthermore, biomass‐derived AC production results in less than half the greenhouse gas emissions of coal‐based AC, with 8.60 kg CO_2_ equivalent per kg of biomass‐derived AC produced compared to 18.28 kg CO_2_ equivalent for coal AC.^[^ ^48^ ^]^

**Figure 5:**
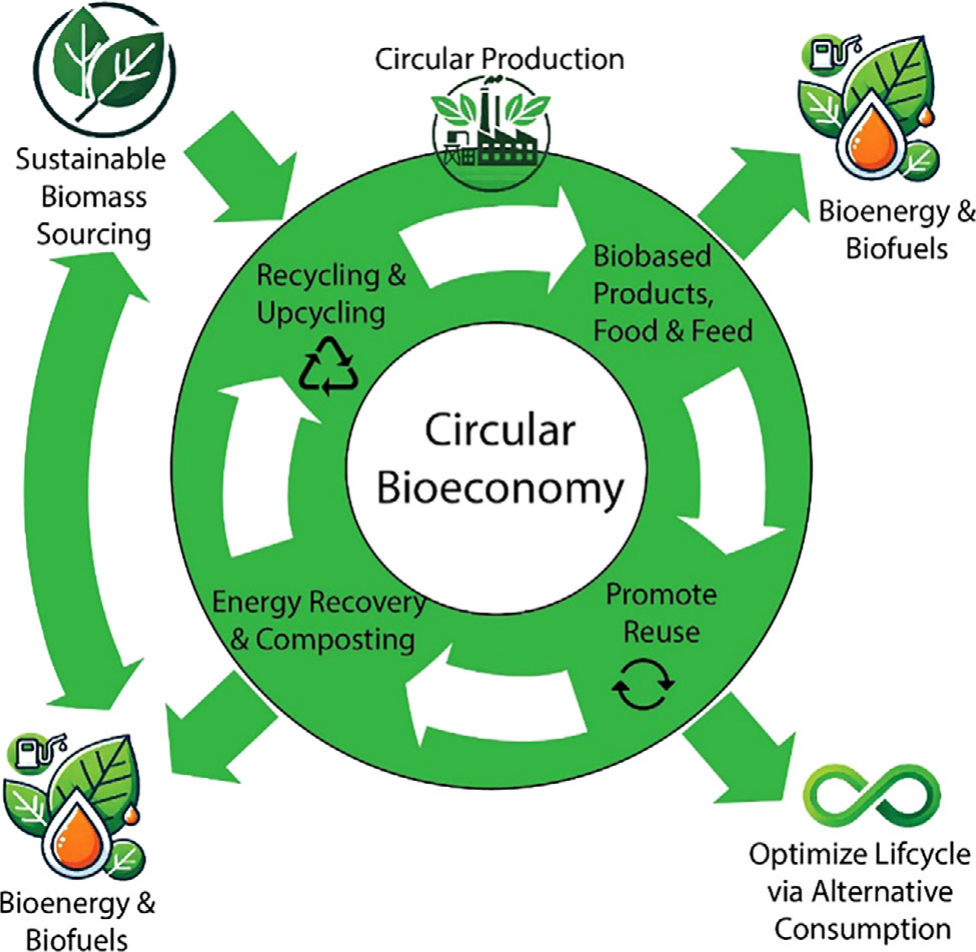
Components and structure of the circular bioeconomy.

**Figure 6:**
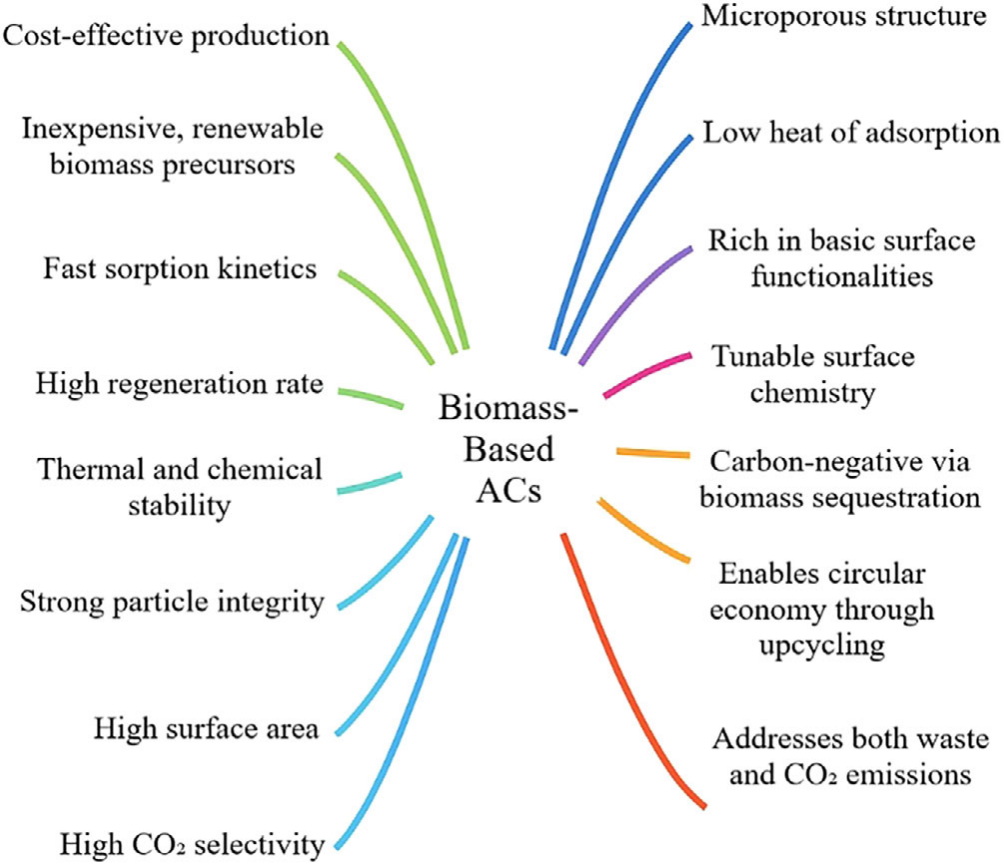
Key advantages of biomass‐derived ACs for CO_2_ capture. Information collected from studies on material properties, production costs, and environmental benefits.^[^ ^36^, ^37^, ^38^, ^39^, ^48^, ^50^, ^51^ ^]^

**Figure** **5** illustrates the core principles and structure of a circular bioeconomy, which relies on sustainably sourced biomass. In this framework, biomass is utilized efficiently through circular production models, such as using integrated, multi‐output production systems and biorefineries. The target in circular production is to maximize the use and upcycling of biomass waste into value‐added products and to optimize the use of such resources through recycling and cascading processes. In fact, the optimization of biomass usage focuses on all three pillars of sustainability simultaneously: economic, environmental, and social benefits. Cascading, or the sequential use of biomass in multiple stages, aims to maintain biomass quality by following the bio‐based value pyramid and the waste hierarchy where appropriate. This method will maximize the value and efficiency of biomass resources.^[^ ^49^ ^]^ Overall, the key advantages of biomass‐derived ACs for CO_2_ capture are summarized in **Figure** **6**. To contextualize our contribution within the broader literature, **Table** **1** summarizes the focus and scope of recent review articles on biomass‐derived ACs for CO_2_ capture, highlighting the unique aspects and innovations of the present work.

**Table 1:** Summary of recent reviews on biomass‐based ACs for CO_2_ capture.

Reference	Year	Key focus/ Contribution
Singh et al.^[^ ^52^ ^]^	2019	Provides a fundamental understanding of biochar synthesis and formation via pyrolysis, examines the development of activated porous carbons, and explores their potential for CO_2_ capture.
Javed et al.^[^ ^53^ ^]^	2021	Critically analyzes process parameters affecting synthesis and performance of adsorbents for CO_2_ capture. Reviews the impact of biomass type, activation method, and operating conditions on adsorption efficiency.
Azmi et al.^[^ ^50^ ^]^	2022	Reviews waste‐derived precursors for AC in CO_2_ capture, focusing on properties like composition, porosity, and surface functionality. Analyzes effects of activation type, impregnation ratio, and temperature on adsorption performance.
Malini et al.^[^ ^54^ ^]^	2023	Compiles research on AC preparation and modifications (nitrogen doping, metal oxide impregnation) to enhance basicity and CO_2_ adsorption. Highlights effects of carbonization and activation on surface properties
Zaker et al.^[^ ^55^ ^]^	2023	Recommends using nitrogen/sulfur‐rich biomasses (e.g., sludge, microalgae) and calls for further research into mechanisms, techno‐economic viability, and scale‐up for industrial applications
Adeniny et al.^[^ ^56^ ^]^	2023	Reviews production routes, properties, and commercialization challenges of biomass‐derived AC monoliths. Highlights the impact of pre‐treatment, activation, and shaping on material properties, and explores applications in purification, energy storage, and catalysis. Identifies feedstock variability, process optimization, and market acceptance as key barriers, and emphasizes the role of interdisciplinary approaches and data‐driven tools in advancing the field.
Ramalingam et al.^[^ ^57^ ^]^	2024	Highlights advances in extraction, processing, and functionalization, with emphasis on low‐cost, renewable feedstocks. Identifies opportunities in improving synthesis methods and integrating ammonia into the energy system.
Serafin et al.^[^ ^18^ ^]^	2024	Highlights the role of KOH activation, narrow microporosity, and functionalization with nitrogen or metal oxides. Emphasizes the need for optimization, techno‐economic analysis, and scalable regeneration strategies.
Makepa et al.^[^ ^58^ ^]^	2025	Reviews sustainable biomass production and conversion methods alongside CCS strategies. Highlights the role of integrated biorefineries and bioenergy with CCS in achieving negative emissions. Discusses technological advancements, life cycle considerations, and real‐world case studies, emphasizing the need for continued research and global scale‐up.
Khandaker et al.^[^ ^59^ ^]^	2025	Reviews advances in synthesis, activation, and heteroatom doping. Addresses challenges in large‐scale production and calls for further research to optimize performance and scalability.
Umar et al.^[^ ^60^ ^]^	2025	Reviews the structural diversity (0D–3D), synthesis methods, and surface modifications. Highlights their role in circular economy, industrial applicability, and integration with hybrid systems.
Present Work		Reviews recent advances in bio‐based AC for CO_2_ capture, emphasizing the impact of precursor selection, activation methods, and surface modifications on performance, especially under humid conditions. Highlights AI‐driven tools for material optimization and process modeling, while addressing challenges in scalability, regeneration, environmental impact, and economic viability.

In this review, we focus on bio‐based adsorbents for CO_2_ capture, with particular emphasis on ACs. We begin by detailing the various biomass sources and their characteristics, highlighting aspects of sustainability, circularity, and cost‐effectiveness. We then examine activation methods for producing biomass‐derived ACs and discuss surface chemistry modifications aimed at enhancing CO_2_ capture performance and selectivity, especially under humid conditions. The integration of Artificial Intelligence in the design, modeling, and optimization of bio‐based adsorbents is also explored. Finally, we review kinetic and equilibrium models commonly used to evaluate adsorption behavior. Although reactor design plays a critical role in system‐level deployment, this review centers on the material science and performance aspects of biomass‐based sorbents, which form the foundation for future advances in scalable and sustainable carbon capture technologies.

## 2 Bio‐Based Precursors for Adsorbents

**Scheme 1:**
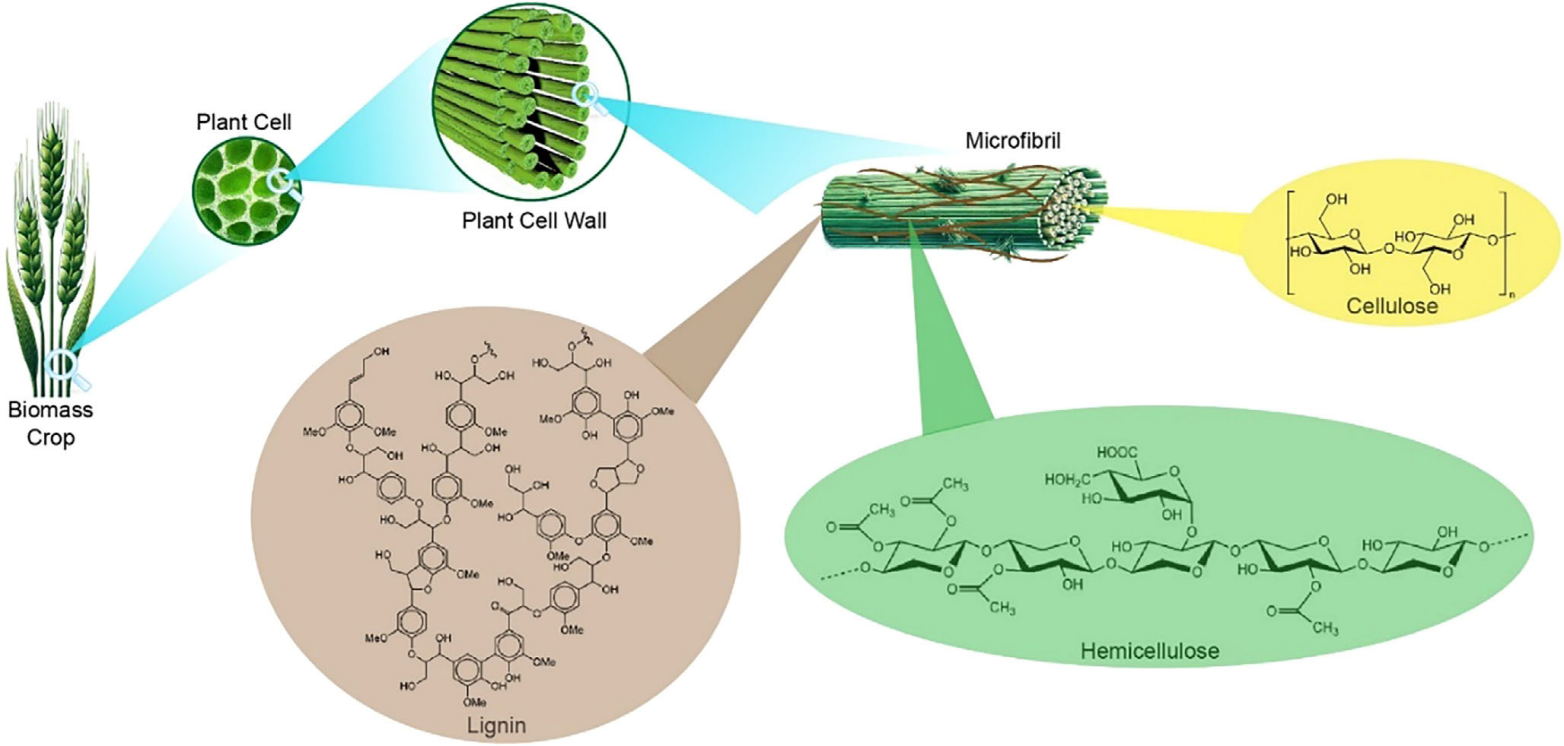
Scheme of lignocellulose biomass and the constituent components (cellulose, lignin, hemicellulose).

Biomass typically consists of natural polymers, including cellulose (40‐50 wt%), lignin (16‐33 wt%), and hemicelluloses (15‐30 wt%), along with extractive materials (1‐10 wt%) and inorganic ashes (0.5‐15 wt%), as illustrated in **Scheme** **1**.^[^ ^61^, ^62^ ^]^ The proportions of the components vary depending on the species, origin, and processing of the lignocellulosic biomass.^[^ ^61^, ^62^ ^]^ Lignin presents the maximum C content, ≈58.6%, which favors AC production.^[^ ^63^, ^64^ ^]^ Other substances such as tannins, proteins, waxes, and chlorophyll also contribute to the significant levels of C, O, and H.^[^ ^64^ ^]^

### 2.1 Biomass Composition

**Scheme 2:**
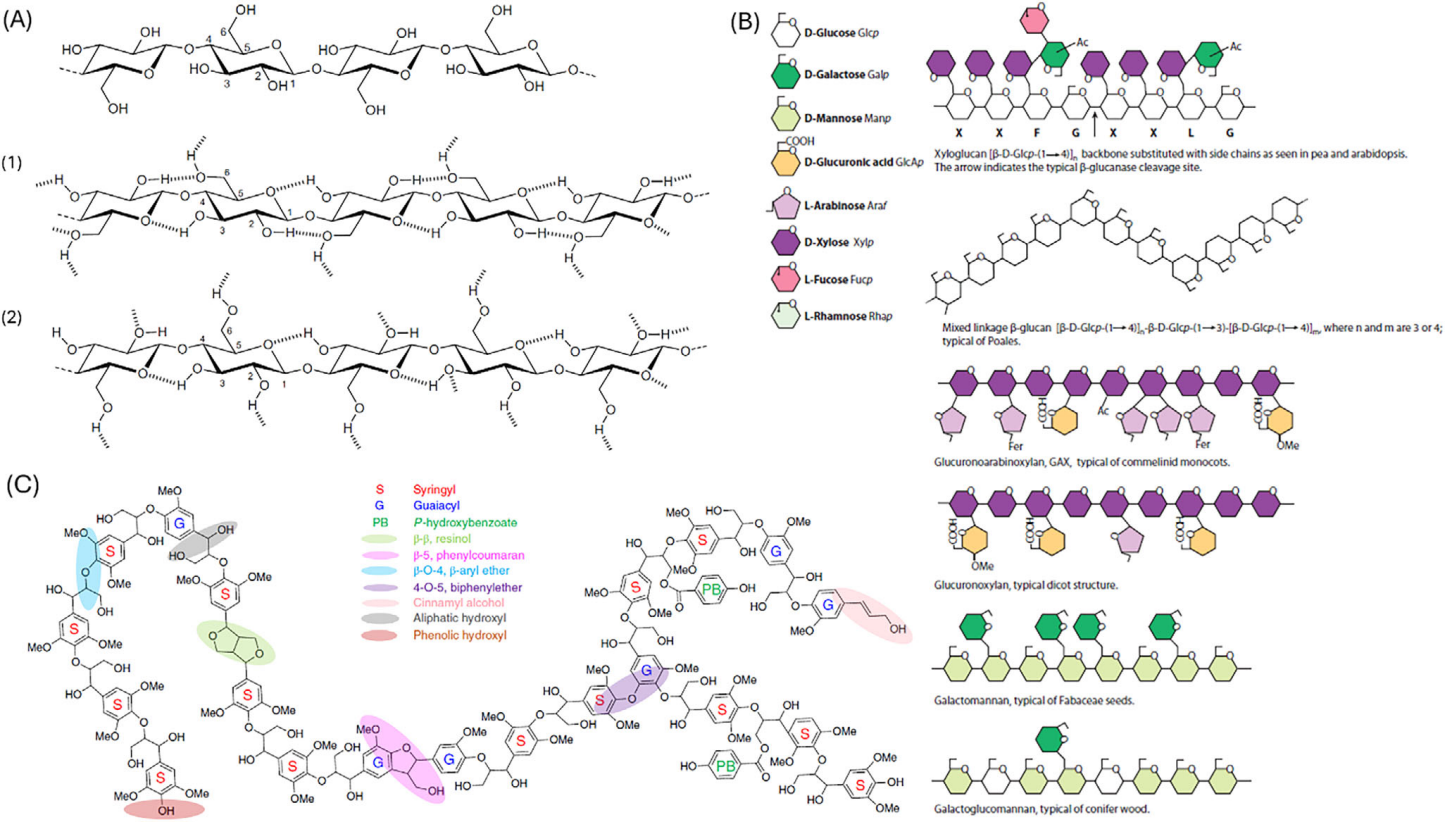
Representative chemical formula of A) cellulose including hydrogen bonding of 1) cellulose I and 2) cellulose II (reproduced with permission from Heinze et al.^[^ ^69^ ^]^ and Tashiro et al.,^[^ ^70^ ^]^ copyright 1991, with permission from Elsevier); B) hemicelluloses (reproduced with permission from Scheller et al.^[^ ^71^ ^]^); C) lignin (reproduced with permission from Meng et al.^[^ ^72^ ^]^ and Vanholme et al.^[^ ^73^ ^]^).

Cellulose is a polymer of D‐glucose units linked by *β*‐1,4 glycosidic bonds with the degree of cellulose polymerization ranges from 100 to 20 000 anhydroglucose units.^[^ ^65^ ^]^ The semicrystalline structures of cellulose offer more resistance to high temperature treatment with intramolecular and intermolecular hydrogen bonding, and the high thermal stability is especially obvious for cellulose of high molar mass.^[^ ^62^, ^63^, ^64^, ^66^ ^]^ In native cellulose I, parallel chains form two‐dimensional hydrogen‐bonded sheets that stack on top of each other via non‐polar (hydrophobic) interactions​. The representative chemical structures of cellulose, hemicelluloses, and lignin are shown in **Scheme** **2**. This combination of strong hydrogen bonding and regular chain stacking produces robust crystalline microfibrils (contributing to the strength of cellulosic fibers) and gives rise to a prominent X‐ray diffraction peak at 2*θ* ≈ 22.5°, corresponding to the characteristic (200) reflection of the ordered cellulose lattice.^[^ ^67^, ^68^ ^]^

Hemicelluloses are polysaccharides enriched in the secondary cell wall. The hemicelluloses in grasses and agricultural residues is mainly glucuronoarabinoxylan, which has a xylose backbone decorated with arabinose, glucuronic acid, and acetyl side‐groups.^[^ ^71^ ^]^ Softwood hemicellulose contains arabino‐4‐O‐methylglucurono‐xylans (up to 10 wt%) and O‐acetyl‐galactoglucomannans (14‐20 wt%).^[^ ^63^, ^74^ ^]^ Hardwood mainly contains O‐acetyl‐4‐O‐methylglucuronoxylan (20‐35 wt%) and trace amounts of glucomannan (2‐4 wt%).^[^ ^71^, ^74^ ^]^ Due to its amorphous structure and lower molecular weight compared to cellulose and lignin, hemicellulose can be easily hydrolyzed into oligosaccharides and monosaccharides.^[^ ^75^ ^]^ As a result, the yields of hemicelluloses after carbonization are relatively low compared with cellulose and lignin.^[^ ^64^ ^]^

Lignin provides structural support in plants and is produced globally at a rate of 40–60 million tons per year. It is an amorphous polyphenol structure composed of three canonical monolignols: p‐coumaryl alcohol, coniferyl alcohol, and sinapyl alcohol, depending on the number of ortho methoxy groups.^[^ ^63^ ^]^ These monolignols form the hydroxyphenyl (H), guaiacyl (G), and syringyl (S) subunits, respectively.^[^ ^76^ ^]^ In perennial grasses and agricultural residues, all three subunits are present, whereas in softwood, the main subunit is coniferyl alcohol, and in hardwood, both coniferyl and sinapyl alcohol are predominant.^[^ ^77^ ^]^ Some minor monomers, such as caffeyl alcohol, tricin, and piceatannol, also contribute to biomass lignification.^[^ ^78^ ^]^

Notably, lignin plays a crucial role in adsorption activities, making it a key component in the production of ACs.^[^ ^79^ ^]^ However, its concentration in biomass varies significantly. For example, flax bast fibers contain only about 2.5–5% lignin,^[^ ^80^ ^]^ whereas flax hurds (woody core) contain significantly more lignin, typically around 20–30%.^[^ ^81^ ^]^ In contrast, coconut coir contains nearly 60% lignin.^[^ ^82^ ^]^ Lignocellulosic materials are highly valued as precursors for AC production due to their high carbon content. However, their composition is influenced by several factors such as plant type, environmental conditions, plant age, and harvest season before being converted into ACs.^[^ ^83^ ^]^

Derived from concepts of biofuel generations utilizing varying biomasses, several types of feedstocks for bio‐based AC production are summarized as follows. The first one is edible crops, municipal solid waste, and biowaste, such as some waste mixtures from coffee grounds food, and sewage sludge,^[^ ^84^, ^85^, ^86^ ^]^ bamboo waste,^[^ ^87^ ^]^ biomass tar,^[^ ^88^ ^]^ and waste wood.^[^ ^89^ ^]^ According to the European Commission, biowaste encompasses waste generated in gardens and parks, kitchen waste, household food processing waste, and food residues.^[^ ^90^ ^]^ Additional waste comes from agricultural and agro‐industrial waste, by‐products from paper and food processing, meat waste, cattle manure, domestic and sewage waste, and residues from forest operations.^[^ ^91^ ^]^ Like biomass, biowaste contains cellulose and also includes starch, protein, collagen, lipids, and chitin.^[^ ^83^ ^]^ These materials can be used as feedstock for manufacturing bio‐based adsorbents.

The second type is non‐edible crops and agricultural residues including rice husk,^[^ ^29^ ^]^ rice straw,^[^ ^92^ ^]^ walnut shell,^[^ ^93^ ^]^ flawn grass,^[^ ^94^ ^]^ microalgae,^[^ ^95^ ^]^ waste tobacco stem,^[^ ^96^ ^]^ pine nutshell and sawdust,^[^ ^97^, ^98^, ^99^, ^100^ ^]^ sugar beet leaves,^[^ ^101^ ^]^ date palm leaf,^[^ ^102^ ^]^ olive stones,^[^ ^103^ ^]^ and corncobs.^[^ ^104^ ^]^ The third type is lignocellulosic materials such as biomass and their extracted compositions, including birch,^[^ ^105^ ^]^ Eucalyptus wood,^[^ ^106^ ^]^ Subabul wood,^[^ ^107^ ^]^ sugarcane,^[^ ^108^ ^]^ lignins,^[^ ^92^ ^]^ glucose,^[^ ^109^, ^110^, ^111^, ^112^ ^]^ and cellulose.^[^ ^64^, ^113^, ^114^ ^]^ Some other non‐lignocellulosic biomaterials are also studied for bio‐based AC production, such as shrimp shell,^[^ ^115^, ^116^ ^]^ jellyfish‐based biomass,^[^ ^117^ ^]^ casein,^[^ ^118^ ^]^ and spider silk.^[^ ^119^ ^]^

### 2.2 Biomass Thermal Conversion to AC

During the pyrolysis or carbonization at elevated temperatures, the three main components of biomass—hemicellulose, cellulose, and lignin—undergo varying degradation and carbonization processes to produce flue gas, condensable (bio‐oil), and solid carbon (biochar). The degradation of hemicellulose and cellulose occurs at relatively low temperatures, with the weight loss of hemicellulose mainly at 220–315 °C, and that of cellulose at 315–400 °C. However, lignin is more difficult to generate volatile degradation components, as its weight loss occurs in a broad temperature range (160–900 °C) with high carbon yield (40 wt%). Importantly, carbonization initially begins as an endothermic process, but at higher temperatures, it transitions to an exothermic stage as the further decomposition of components, particularly hemicellulose and lignin, releases heat.^[^ ^120^, ^121^ ^]^

The flue gas produced during carbonization at 350–650 °C mainly consists of CO, CO_2_, CH_4_, and H_2_ for each biomass component, including cellulose, hemicellulose, and lignin.^[^ ^122^, ^123^ ^]^ Studies show that hemicellulose yields a higher amount of CO_2_, cellulose produces more CO, and lignin generates higher yields of H_2_ and CH_4_.^[^ ^120^ ^]^

The typical bio‐oil formed after pyrolysis includes acids, ketones, aldehydes, esters, benzenes, alcohols, alkenes, phenols, alkanes, carbohydrates, and other compounds. Cellulose serves as the primary source of carbohydrates, while phenols are derived from lignin. The bio‐oil from xylan mainly consists of acids, ketones, aldehydes, and phenols. The bio‐oil yields for cellulose, xylan, and lignin are 65 wt%, 53 wt%, and 40 wt% at 400, 450, and 500 °C, respectively.^[^ ^122^ ^]^

The carbon yields for the three components decrease with increasing temperature, while the gas and bio‐oil yields increase with rising temperature, reaching a maximum before declining afterward.^[^ ^122^ ^]^ Lignin is also considered the main contributor to ACs.^[^ ^64^ ^]^ The mixture of cellulose, hemicellulose, and lignin displays a three‐dimensional porous structure, including macropores, mesopores, and micropores, when less than 50 wt% of lignin is present during carbonization.^[^ ^123^ ^]^ Additionally, the evolution of the mean pore size as a function of the specific pore volume indicates that each component contributes to the porosity of ACs, regardless of its weight fraction.^[^ ^64^ ^]^ However, some reports have indicated that cellulose and hemicellulose generate micropores at 400–900 °C, while lignin remains relatively stable and forms nonporous carbon. When salt, such as KHCO_3_, is introduced, the carbonization mechanisms vary for each component. Dehydration dominates in cellulose and hemicellulose, producing microporosity, while in lignin, β‐O‐4 bonds undergo homolysis, increasing benzene‐containing units and forming carbon nanosheets.^[^ ^123^ ^]^

AC is produced from *Pinus radiata* sawdust using KOH and ZnCl_2_ as activation agents through dehydrogenation and intermolecular dehydration.^[^ ^98^ ^]^ Activation with KOH at high temperatures (850 °C) creates high porosity by disrupting the atomic arrangement of the biomass, regardless of the KOH ratio. At lower temperatures (600 °C), however, the hydrogen content of carbon increases. Similar trends are observed for ZnCl_2_ activation, though with less pronounced effects across different temperatures.^[^ ^124^ ^]^ A high oxygen content is present in AC produced by KOH activation, likely due to gasification and the formation of oxygen‐containing groups at lower temperatures. At higher temperatures, increased decomposition reactions lead to a reduction in oxygen and hydrogen content.^[^ ^125^ ^]^ The decrease in oxygen content during ZnCl_2_ activation, however, results from catalytic dehydration.^[^ ^126^ ^]^

For a binary salt treatment system, synergistic effects are reported. For example, the one‐step carbonization of chitin using ZnCl_2_‐CaCl_2_ (Zn‐Ca) molten salt produces hierarchically tunable micro/mesoporous carbon. Among these salts, ZnCl_2_ primarily generates micropores, while CaCl_2_ contributes to mesopore formation.^[^ ^127^ ^]^

### 2.3 Bio‐Based AC Advantages and Challenges

The CO_2_ uptake capacity of AC is often lower than that of MOFs and zeolites, particularly under conditions of low CO_2_ partial pressure and low density. Nonetheless, ACs offer significant advantages, such as excellent regeneration capabilities and stable capture performance across multiple adsorption/desorption cycles.^[^ ^14^ ^]^

ACs can be tailored with micro‐ and mesoporosity depending on the choice of raw material. They may also incorporate heteroatoms such as nitrogen, hydrogen, sulfur, and phosphorus, which originate from the biomass precursor or the activation process. These heteroatoms contribute to surface functional groups that play important roles in enhancing CO_2_ adsorption capacity and selectivity.

When selecting the optimal bio‐derived precursor for AC production, it is important to consider several key factors: precursor availability, economic feasibility, and eco‐friendly, non‐hazardous properties. Lignocellulosic biomass stands out among available sources due to its numerous advantages, including abundant, renewable, and diverse precursors; high reactivity, which simplifies the synthesis process; low processing costs, thanks to reduced waste disposal expenses and significantly lower environmental impact.

Two critical characteristics of biomass‐derived ACs are their high carbon content and low ash content.^[^ ^83^, ^128^ ^]^ Plant‐based biomass offers numerous parts for AC production, such as shells, cores, stems, flowers, seeds, peels, stones, leaves, fruits, and husks. Additionally, aquatic biomass like grass, starch, cakes, and fibers, as well as other unconventional sources, have also been recognized as viable precursors for AC production.^[^ ^83^ ^]^

While biomass precursors offer various properties that can produce AC with effective characteristics, the diversity in the chemical composition of lignocellulosic biomass presents a challenge for scaling up AC production. Therefore, the preparation methods and the chemical composition of the biomass precursor play a critical role in determining the final properties of AC, such as pore size, surface area, total pore volume, production yield, and overall adsorption performance.^[^ ^129^, ^130^, ^131^ ^]^ For instance, research indicates that biomass precursors with a higher carbon content, produce AC with a superior surface area.^[^ ^132^ ^]^

**Figure 7:**
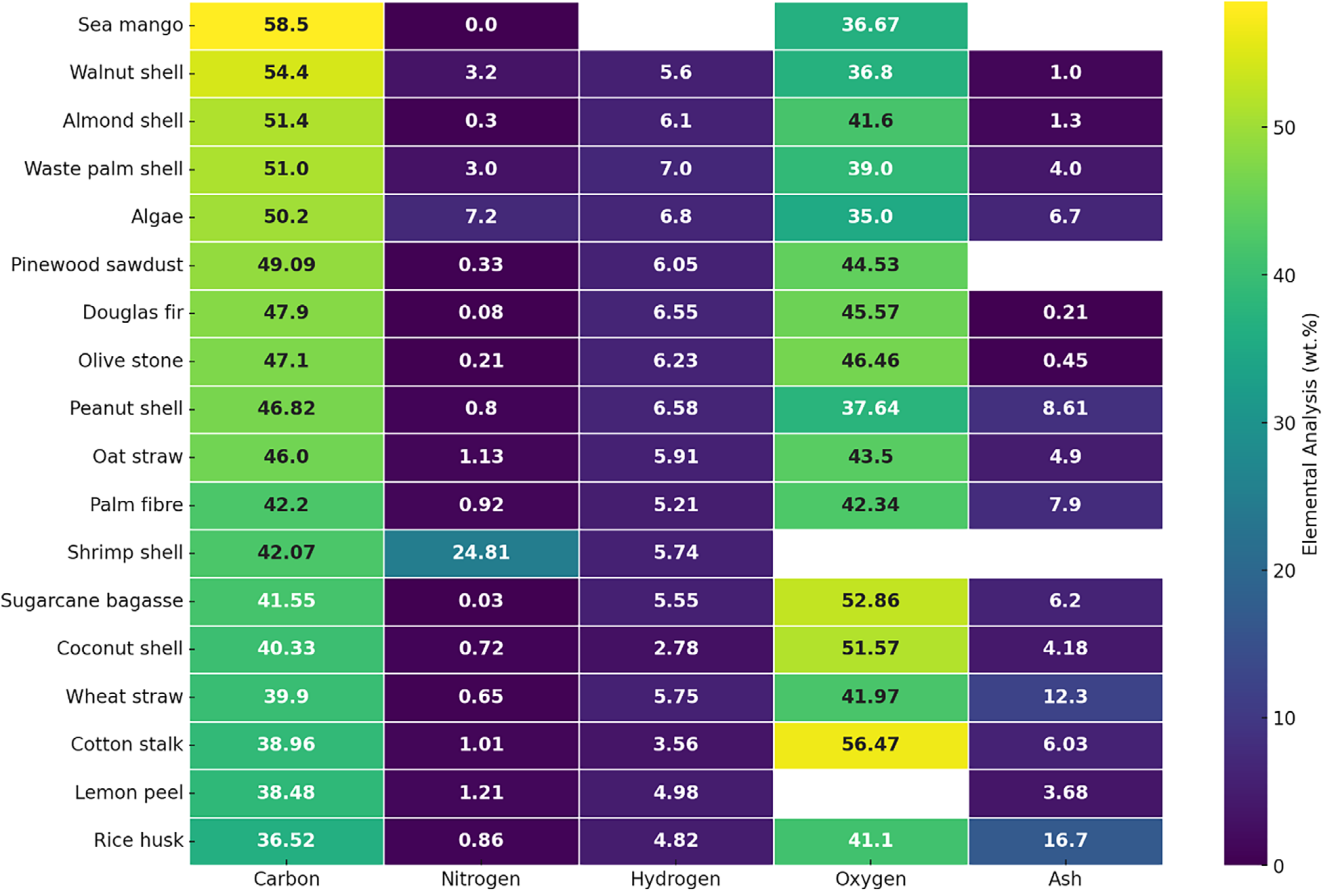
Heat map for elemental analysis of various biomass for AC production. The raw materials analyzed are coconut shell,^[^ ^136^ ^]^ rice husk,^[^ ^137^ ^]^ sugarcane bagasse,^[^ ^137^ ^]^ pinewood sawdust,^[^ ^138^ ^]^ wheat straw,^[^ ^139^ ^]^ olive stone,^[^ ^140^ ^]^ algae,^[^ ^141^ ^]^ shrimp shell,^[^ ^116^ ^]^ almond shell,^[^ ^31^ ^]^ walnut shell,^[^ ^143^ ^]^ peanut shell,^[^ ^142^ ^]^ lemon peel,^[^ ^143^ ^]^ palm fiber,^[^ ^134^ ^]^ cotton stalk,^[^ ^144^ ^]^ oat straw,^[^ ^145^ ^]^ sea mango,^[^ ^146^ ^]^ waste palm shell,^[^ ^147^ ^]^ and Douglas fir.^[^ ^148^ ^]^

**Figure** **7** shows the heatmap of the elemental analysis of various biomass used for AC production. According to the literature, coconut shells are frequently investigated as a precursor material owing to their strong mechanical properties and their relative high carbon content (40.33 wt.%).^[^ ^50^ ^]^ As illustrated in the figure below, Sea mango, walnut shell, almond shell, waste palm shell, algae, and Douglas fir exhibit the highest carbon content, leading to a higher yield of AC.^[^ ^133^, ^134^, ^135^ ^]^ Additionally, the high carbon content in these biomass materials contributes to the formation of microporous structures.

In terms of nitrogen content in biomass, according to Figure 7, shrimp shell and algae show the highest nitrogen content, with 24.81 wt.% and 7.20 wt.%, respectively. Studies have shown that a high nitrogen content in biomass can also improve CO_2_ adsorption capacity, regardless of carbon composition.^[^ ^115^, ^149^ ^]^ Although it was previously mentioned that high carbon content in biomass correlates with a high surface area of AC, recent studies have revealed that the presence of heteroatom‐rich materials during carbonization and activation can enhance both surface area and carbon yield. For example, the addition of melamine, sodium thiosulfate, and KOH has been reported to double the AC yield due to enhanced thermal stability, which will be explored in the next sections.^[^ ^28^ ^]^

Another important factor to consider when selecting a suitable biomass precursor is low ash content. As shown in Figure 7, Douglas fir and olive stone have ash contents below 1 wt.%. The significance of low ash content in AC production lies not just in porosity development, but in creating purer and more inert adsorption sites. Lower ash content indicates fewer inorganic impurities, which facilitates the formation of a more robust and extensive network of micropores and mesopores.^[^ ^150^ ^]^

Based on the discussion above, identifying the carbon content and composition of biomass precursors is crucial, as these factors significantly influence the properties of AC. Fixed carbon and volatile matter contents are commonly used to assess the carbonization effects and the porosity development. During carbonization, pore formation is promoted by increased material mobility and a reduction in volatile matter content.^[^ ^50^ ^]^ Additionally, the pore structure of AC plays a key role in CO_2_ capture. Section 4.1 explores the relationship between pore size distribution and adsorption performance, detailing the roles of micropores, mesopores, and macropores.

The carbon content in biomass results from the photosynthetic fixation of CO_2_ and varies across different types of biomass precursors. For instance, corn stover contains 51.2% cellulose, 30.7% hemicellulose, and 14.4% lignin, resulting in a reported carbon content of 49.3%.^[^ ^151^ ^]^ In contrast, jatropha wood has a carbon content of 42.2%, with a composition of 48.03% cellulose, 24.7% hemicellulose, and 24.28% lignin.^[^ ^152^ ^]^ Furthermore, it is observed that biomass with higher lignin content tends to form spherical porous structures during AC production, while those with higher cellulose content typically develop cylindrical pore structures.^[^ ^141^, ^144^, ^153^, ^154^ ^]^ Similar findings highlight the major role of precursor type in the development of AC texture.^[^ ^155^ ^]^

The literature indicates that coconut shells, rice husk, palm kernel shell, and bagasse are among the most used agricultural biowaste for AC production. This prevalence is due to their abundance, high carbon content, and relatively low ash content.^[^ ^156^, ^157^, ^158^ ^]^ Given the variability in compositional properties of waste materials from different sources, producing optimal AC for CO_2_ adsorption remains a complex challenge. Therefore, it is essential to conduct thorough studies to understand the carbon content and composition of biomass before scaling up production, as these factors directly influence the properties of the resulting AC.

**Table** **2** summarizes recent advancements in the use of biomass‐derived AC sorbents for CO_2_ capture from 2023 to 2024. A crucial factor for the high efficiency of ACs in CO_2_ uptake is their surface area and pore volume, with larger surface areas and pore volumes typically correlating with higher gas adsorption capacities. Among the studies listed, AC from d(+)‐glucose exhibited the highest surface area of 3572 m^2^ g^−1^.^[^ ^109^ ^]^

**Table 2:** Summary of the key characteristics of selected biomass‐derived AC reported in the literature from 2023–2024.

Carbon precursors	Activation method	Surface area [m^2^ g^−1^]	Total pore volume [cm^3^ g^−1^]	CO_2_ capacity [mmol g^−1^, 1 bar]	CO_2_/N_2_ Selectivity (IAST)	Refs.
Bamboo charcoal	Physical (H_2_O, CO_2_) and chemical (ZnCl_2_, KOH, H_3_PO_4_)	91‐526	0.03‐0.46	3.35‐3.81 at 25 °C	4‐10	Zhang et al.^[^ ^159^ ^]^
Bamboo shoot shells	Chemical (K_2_CO_3_)	1439	0.58	3.44 at 25 °C	21.0	Wu et al.^[^ ^160^ ^]^
Bamboo shoot shells	Chemical (K_2_CO_3_)	1985	0.81	3.60 at 25 °C		Wu et al.^[^ ^161^ ^]^
Brazilian nutshell waste	Hydrothermal	1097	0.46	5.2 at 0 °C		Cruz Jr et al.^[^ ^162^ ^]^
Brazil nut shells	Chemical (KOH)	2730	1.40	3.8 at 25 °C		de Souza et al.^[^ ^163^ ^]^
Coconut shell	Physical (H_2_O, CO_2_) and chemical (ZnCl_2_, KOH, H_3_PO_4_)	983‐1352	0.34‐0.67	3.44‐3.74 at 25 °C	4‐12	Zhang et al.^[^ ^159^ ^]^
Corncob	Chemical (KOH)	2697	1.25	5.12 at 25 °C	20	Bai et al.^[^ ^164^ ^]^
Corncob powder	Chemical (K_2_CO_3_ and urea)	1016	0.56	5.69 at 0 °C	38	Li et al.^[^ ^165^ ^]^
Cotton fibers	Chemical activation (KOH)	1371	0.61	4.43 at 25 °C		Xing et al.^[^ ^166^ ^]^
Cupuassu shell	Chemical (KOH)	2486	1.02	4.4 at 25 °C		Cruz Jr et al.^[^ ^167^ ^]^
D(+)‐glucose	Chemical (KOH)	3572	1.71	5.9 at 0 °C		Geng et al.^[^ ^109^ ^]^
Fern Leaves	Chemical (KOH) and Physical (CO_2_)	1016	0.611	3.58 at 25 °C	–	Serafin et al.^[^ ^168^ ^]^
Grass‐dairy co‐digestate	Chemical (H_3_PO_4_)	168	0.10	0.55 at 25 °C		Quan et al.^[^ ^169^ ^]^
Gum arabic tree seed shell	Chemical (KOH)	1472	0.77	3.42 at 25 °C		Goskula et al.^[^ ^170^ ^]^
Jellyfish‐based biomass	Physical (H_2_O, CO_2_) and chemical (ZnCl_2_, KOH, H_3_PO_4_) and Chemical (NaOH)	2092	1.29	5.18 at 25 °C		Ha et al.^[^ ^117^ ^]^
Lemon peel	Hydrothermal and Chemical (KOH)	1113	0.56	4.5 at 0 °C	19.9	Weldekidan et al.^[^ ^171^ ^]^
Marine biomass	Microwave (MgO and N‐doping)	300	0.29	4.79 at 100 °C	80	Luo et al.^[^ ^172^ ^]^
Microcrystalline cellulose	Hydrothermal and Chemical (KOH)	2296	0.66	7.07 at 25 °C		Taher et al.^[^ ^113^ ^]^
Olive stones	Chemical (KOH)	969	0.54	4.3 at 30 °C	28.4	Serafin et al.^[^ ^173^ ^]^
Pecan nutshell	Microwave and Chemical (H_3_PO_4_)	1145	0.59	2.7 at 25 °C	> 38	Duran et al.^[^ ^174^ ^]^
Pine needles	Chemical (KOH)	1556	0.56	4.05 at 25 °C		Lim et al.^[^ ^175^ ^]^
Pine sawdust	Physicochemical (KOH and CO_2_)	2216	1.11	6.35 at 0 °C	12	Patel et al.^[^ ^97^ ^]^
Pine sawdust	Chemical (KOH and ZnCl_2_)	2864	1.46	5.79 at 0 °C		Pimentel et al.^[^ ^98^ ^]^
Rice husk	Chemical (KOH)	755	0.40	1.55 at 50 °C	30	Nandi et al.^[^ ^176^ ^]^
Soluble starch	Chemical (melamine, NaHCO3)	2477	1.93	2.85 at 25 °C	12	Zhang et al.^[^ ^177^ ^]^
Spider silk	Chemical (KOH)	2730	1.56	23.6 at 0 °C	13	Muhammad et al.^[^ ^119^ ^]^
Subabul wood biomass	Physical and Chemical (H_3_PO_4_)	823	0.26	4.52 at 25 °C		Mallesh et al.^[^ ^107^ ^]^
Sugarcane	Hydrothermal and pyrolysis	938	0.47	2.8 at 25 °C	58	Wang et al.^[^ ^108^ ^]^
Sugarcane pith	Physical (N_2_) and Chemical (H_2_SO_4_)	725	0.34	2.4 at 25 °C	20	Koli et al.^[^ ^107^ ^]^
Sugarcane pith	Physical (N_2_)	726	0.34	2.35 at 25 °C	20	Koli et al.^[^ ^178^ ^]^
Tannic acid	Chemical (KOH)	2553	1.17	4.29 at 25 °C	21	Zhang et al.^[^ ^179^ ^]^
Terminalia catappa shell	Physical (N_2_)	616	0.26	2.73 at 15 °C	42	Koli et al.^[^ ^180^ ^]^
Walnut shell	Chemical (KOH and N, S doping)	1412	0.77	5.51 at 25 °C	26	Cao et al.^[^ ^181^ ^]^
Walnut shell	Chemical (KOH)	1868	1.06	5.1 at 25 °C	43.6	Serafin et al.^[^ ^182^ ^]^
Waste distiller's grains	Chemical (KOH and N, S‐doping)	2537	0.98	7.02 at 0 °C	32	Luo et al.,^[^ ^183^ ^]^
Waste oleaster seed	Chemical (KOH)	564	0.53	2.78 at 25 °C		Athari et al.^[^ ^184^ ^]^
wood pellet	Physical (H_2_O, CO_2_) and chemical (ZnCl_2_, KOH, H_3_PO_4_) Chemical (NaOH)	3‐438	0.001‐0.24	3.83‐4.10 at 25 °C	4‐9	Zhang et al.^[^ ^159^ ^]^

The activation method plays a vital role in creating pores and defining the properties of the AC product. According to the table, chemical activation using KOH remains highly favorable for producing high‐performance AC. Among the various biomass sources, spider silk activated by KOH showed the highest CO_2_ uptake capacity of 23.6 mmol g^−1^ at 0 °C and 1 bar, indicating outstanding low‐temperature carbon capture.^[^ ^119^ ^]^ Following this, microcrystalline cellulose and waste distiller's grains demonstrated significant uptakes of 7.07 mmol g^−1^ at 25 °C and 7.02 mmol g^−1^ at 0 °C, respectively.^[^ ^113^, ^183^ ^]^ However, it is important to note that these biomass sources generally result in low carbon yields, which may limit large‐scale application. This trade‐off between high CO_2_ uptake and carbon yield should be considered when selecting suitable precursors for AC production.

Heteroatom doping with nitrogen and sulfur enhances both uptake and gas selectivity. This is exemplified by marine biomass, which exhibits a CO_2_/N_2_ selectivity of 80 at 100 °C, indicating great potential for high‐temperature CO_2_ capture applications.^[^ ^172^ ^]^ The second‐highest selectivity observed is for sugarcane‐derived porous carbon from sugarcane pieces, with a CO_2_/N_2_ selectivity of 58 at 25 °C. The doping method will be discussed in the upcoming sections.^[^ ^108^ ^]^

Combining different activation techniques, such as physical and chemical or chemical and hydrothermal methods, can yield superior AC products with improved textural properties and consequently higher CO_2_ uptake. Table 2 illustrates the diverse biomass sources utilized to produce AC, ranging from agricultural waste (e.g., rice husk, sugarcane pith) to unconventional materials (e.g., spider silk, jellyfish‐based biomass). This diversity underscores the versatility of this approach, while the use of region‐specific waste materials highlights its potential to address sustainability and waste management challenges. Utilizing diverse biomass, from agricultural waste (e.g., rice husk, sugarcane pith) to unconventional sources (e.g., spider silk, jellyfish‐based biomass), not only addresses waste management issues but also provides region‐specific solutions. Additionally, biobased materials have shown great promise in the synthesis of value‐added products from CO_2_.^[^ ^185^, ^186^, ^187^, ^188^ ^]^ The activation techniques and chemical modification of ACs will be thoroughly discussed in Sections 3 and 4.

## 3 Activation Techniques for Bio‐Based ACs

The CO_2_ adsorption capacity of biomass‐derived carbon materials is influenced by both the physical structure and surface chemistry of the materials.^[^ ^189^, ^190^, ^191^ ^]^ The physical structure—particularly pore size, morphology, and specific surface area—dictates physisorption of CO_2_ via physical trapping. Literature shows that ultramicropores between 0.3 and 0.8 nm are favorable for CO_2_ adsorption, with pore sizes below 0.5 nm being the most effective due to CO_2_’s molecular diameter of 0.33 nm.^[^ ^100^ ^]^ Surface chemistry, mainly the polarity, acidity, and basicity of the carbon materials, influences the chemisorption of CO_2_ via the interactions, including hydrogen bonding, van der Waals forces, and Lewis acid–base interactions. Additionally, surface chemistry is key to selectively adsorbing CO_2_ in mixed gas environments such as under varying moisture conditions.

To tailor biomass‐derived carbon materials with a high specific surface area and a desired porous structure for efficient CO_2_ adsorption, various activation methods have been established. Activation techniques are generally divided into two categories: physical and chemical activation. As reflected by the name, physical activation creates pores without the use of chemicals, while chemical activation relies on chemicals to create a porous structure. Each method has distinct advantages and limitations regarding simplicity, energy efficiency, cost, environmental impact, as well as the resulting porosity, surface chemistry, and carbon capture performance. It needs to be noted that although activations also alter the surface properties to some extent especially for chemical activation, the main purpose is for pore creation. Surface properties of AC can be modified with specific treatments such as N‐doping and/or metal‐doping that are discussed in more detail in Section 4.

### 3.1 Physical Activation

Physical activation is a widely adopted method for producing AC due to its simplicity and the absence of chemical reagents. It typically involves two steps: carbonization and activation. Carbonization can be carried out by pyrolysis of biomass, where it undergoes a thermal treatment (400 to 850 °C) in an inert gas atmosphere (e.g., N_2_ or He_2_) to eliminate elements such as hydrogen, oxygen, nitrogen, sulfur, etc., leaving behind carbon‐rich material, known as biochar. However, pristine biochar has limited porosity, surface area, and surface properties required for efficient CO_2_ adsorption.^[^ ^189^ ^]^ Hence, a second step, activation, is performed to develop a highly porous structure by gasifying a portion of the carbon at temperatures above 700 °C. This process significantly increases the material's surface area and is typically carried out using gases such as CO_2_ or steam.

#### 3.1.1 CO_2_ Activation

CO_2_ activation is the most adopted physical activation method for biochar activation. Because CO_2_ activation creates relatively uniform micropores with the sizes that are beneficial for carbon capture, the role of pore size in adsorption performance is further discussed in Section 4.1. CO_2_ activation is achieved via Boudouard reaction, and the reaction mechanism is known as oxygen–carbon exchange proposed by Ergun.^[^ ^192^ ^]^ During this process, CO_2_ first adsorbs onto free carbon active sites (C_(f)_) on the surface of biochar through chemisorption, forming a reactive carbon–oxygen complex (C(O)) and releasing carbon monoxide, as shown in Equation (1). Subsequently, the C(O) species undergoes a transformation in which a carbon atom is removed, transitioning from the solid to the gas phase and leaving behind a new vacant carbon active site. This regenerated site is then available for the next dissociative chemisorption event of CO_2_, as illustrated in Equation (2).

(1) $$C_{f\;}+\;CO_{2}\to C\left(O\right)+\mathrm{CO}$$

(2) $$C\left(O\right)+\;C\to \mathrm{CO}+C_{f}$$

As this reaction is thermodynamically unfavorable (enthalpy positive) and endothermic, hence high temperature (>700 °C) is required to shift the equilibrium to a forward direction.^[^ ^193^, ^194^ ^]^ To achieve the desired pore size, morphology, and specific surface area in AC, parameters like activation temperature, holding time, and CO_2_ flow rate must be carefully adjusted. Numerous studies have shown that a higher activation temperature could facilitate the reaction rate, and hence enhance the micropore volume.^[^ ^195^ ^]^ However, too high activation temperature could widen micropores to mesopores, which is unfavorable for carbon capture. For example, it is reported that the CO_2_ adsorption capacity of CO_2_‐activated biochar sourced from soybean straws increased with the increasing activation temperature from 500 to 800 °C, but then dropped at 900 °C ascribing to reduced micropores although the total surface area further increased.^[^ ^195^ ^]^ Besides, activation temperature also affects the surface functional groups of the biochar. Higher temperature could eliminate the polar functional groups such as hydroxyl, carboxyl or carbonyl groups, resulting in more hydrophobicity of the AC.^[^ ^196^ ^]^ Higher hydrophobicity is beneficial for the selective adsorption of CO_2_ over H_2_O in a moisture‐containing flue gas, since CO_2_ is more hydrophobic than H_2_O. Nevertheless, high hydrophobicity reduces the CO_2_‐AC interactions such as van der Waals interactions or hydrogen bonding, might lead to reduced CO_2_ adsorption capacity. Additionally, CO_2_ activation is affected by CO_2_ flow rate. Higher CO_2_ flow rate enhances diffusion of CO_2_ into biochar pores due to higher concentration gradient.^[^ ^197^ ^]^ However, too high CO_2_ flow rate shortens CO_2_ contact time to biochar, leading to a reduced reaction rate.^[^ ^194^ ^]^ As a result, a moderate CO_2_ low rate from 100 to 150 ml/min with a relatively high activation temperature (> 800 °C) is advocated for CO_2_ activation.^[^ ^191^ ^]^

#### 3.1.2 Steam Activation

Steam activation of biochar is conducted using superheated steam at temperatures ranging from 700 to 900 °C.^[^ ^191^, ^197^ ^]^ Like CO_2_ activation, the primary reaction in steam activation is endothermic, although the overall reaction process is more complex.^[^ ^191^ ^]^ Initially, the reaction begins with the chemisorption of water molecules onto free carbon active sites (Cf), creating a carbon‐oxygen complex (C(O)) and releasing hydrogen gas (H2) (Equation 3). Next, this carbon‐oxygen complex (C(O)) converts into gaseous carbon monoxide (CO) (Equation 4). Following this primary reaction, CO participates in the exothermic water‐gas shift reaction with water, producing CO_2_ and H_2_ (Equation 5). Subsequently, both CO_2_ and H_2_ further react with free carbons, generating CO and methane (CH_4_), respectively (Equations 6 and 7).

(3) $$C_{f}+H_{2}O\to C\left(O\right)+H_{2}$$

(4) $$C\left(O\right)+\;C\to \mathrm{CO}+C_{f}$$

(5) $$\mathrm{CO}+H_{2}O\to CO_{2}+H_{2}$$

(6) $$C{O_{2}}_{+}\;C\to 2\mathrm{CO}$$

(7) $$2H_{2}+\;C\to CH_{4}$$

Similar to CO_2_ activation, higher temperatures in steam activation result in an increased gasification rate due to the endothermic nature of the reaction.^[^ ^198^, ^199^, ^200^ ^]^ However, steam activation is a more aggressive gasification process compared to CO_2_, as it is thermodynamically more favorable, and the smaller size of water molecules allows them to penetrate biochar pores more easily.^[^ ^191^, ^197^, ^201^ ^]^ Consequently, steam activation typically requires either a lower activation temperature or a shorter holding time than CO_2_ activation, making it a more energy‐efficient process. However, the activation temperature and holding time must be adjusted with greater precision; otherwise, there is a risk that micropores may transition into meso‐ and macropores, potentially leading to collapsed pore structures.^[^ ^199^, ^200^ ^]^ Additionally, steam activation normally results in more O‐containing functional groups on AC than CO_2_ activation, thus making the AC more hydrophilic with a stronger interaction with CO_2_.^[^ ^197^, ^199^, ^200^ ^]^

### 3.2 Chemical Activation

Chemical activation is a widely adopted method for producing AC and is more frequently reported in the literature than physical activation.^[^ ^191^, ^202^ ^]^ One advantage of chemical activation is the wider variety of activating agents available, including bases, acids, and salts. Additionally, chemical activation typically yields AC with a higher degree of porosity, which enhances its CO_2_ adsorption capabilities. The process of chemical activation begins with the impregnation of the precursor material (either untreated or carbonized biomass) with a chosen chemical agent, followed by thermal treatment in an inert atmosphere. Throughout literature, potassium hydroxide (KOH) is the most commonly used activating agent across all chemical types for producing AC.^[^ ^191^, ^202^ ^]^ Other activation agents, such as phosphoric acid (H_3_PO_4_), zinc chloride (ZnCl_2_), and potassium carbonate (K_2_CO_3_), are also reported but with moderate frequency.

#### 3.2.1 KOH Activation

KOH has been the most employed chemical used for chemical activation for carbon capture in the literature. One major reason is that KOH normally results in high surface area and high ratio of micropores that are beneficial for CO_2_ adsorption.^[^ ^159^, ^202^ ^]^ KOH activates AC via a series of complex reactions.^[^ ^128^, ^191^, ^197^ ^]^ It is decomposed to K_2_O and H_2_O at the temperature around 400 °C (Equation 8). H_2_O initiates steam activation occurring at the temperature above 500 °C (Equation 9), while K_2_O undergoes oxygen‐carbon exchange reaction with the free carbon of biochar, forming CO and K (Equation 10). K vaporizes at the temperature above its boiling point of 760 °C, which further expands the pores. Meanwhile, KOH can react directly with the free carbon, forming K_2_O, K_2_CO_3_, and H_2_ (Equation 11). K_2_CO_3_ is decomposed into K_2_O and CO_2_ at the temperature above 700 °C (Equation 12). Hence, at the activation temperature above 800 °C, pore creation using KOH is realized due to a series of various reactions, as well as the physical expansion by the vaporized K into the carbon structure.

(8) $$2\mathrm{KOH}\to K_{2}O+H_{2}O$$

(9) $$C+H_{2}O\to \mathrm{CO}+H_{2}$$

(10) $$C+K_{2}O\to \mathrm{CO}+2K$$

(11) $$C+4\mathrm{KOH}\to K_{2}O+K_{2}CO_{3}+2H_{2}$$

(12) $$K_{2}CO_{3}\to K_{2}O+CO_{2}$$

Previous studies have shown that both the amount of KOH and the activation temperature affect the porosity and surface properties of AC. In general, higher KOH concentration and/or higher activation temperature enhance pore creation, resulting in higher surface area and CO_2_ adsorption capacity.^[^ ^164^, ^203^ ^]^ However, too high activation temperature could lead to collapsed pore structure and reduced CO_2_ adsorption capacity. For example, Serafin et al. reported that the surface area and CO_2_ adsorption capacity increased with the increasing activation temperature from 500 to 800 °C, then decreased at the activation temperature of 900 °C due to enlarged and collapsed pore structure.^[^ ^203^ ^]^ In addition, higher KOH amount and/or higher activation temperature led to lower oxygen content and thus lower selective adsorption of CO_2_ over N_2_ probably due to weaker AC‐CO_2_ interactions.^[^ ^164^ ^]^

#### 3.2.2 Other Activation Chemicals

Phosphoric acid (H_3_PO_4_) and salts such as zinc chloride (ZnCl_2_) have been moderately used as chemical activation agents for producing AC from biomass. However, these chemicals are generally less effective at generating micropores compared to potassium hydroxide (KOH). For instance, Zhang et al. activated biochar derived from bamboo, wood pellets, and coconut shells using KOH, H_3_PO_4_, and ZnCl_2_.^[^ ^159^ ^]^ They found that KOH produced the highest micropore volume and total surface area across all three biochars, resulting in the greatest CO_2_ adsorption capacity.

### 3.3 Comparison of Physical Activation and Chemical Activation

The advantages and disadvantages of physical and chemical activation methods have been well discussed across several review papers.^[^ ^191^, ^202^ ^]^ Chemical activation offers several notable advantages in the production of AC. It typically requires lower activation temperatures and shorter holding times, making it more energy‐efficient than physical activation methods. This approach also produces AC with a higher degree of porosity and a larger surface area, both of which are critical for enhancing CO_2_ adsorption capacity. Another benefit is that chemical activation can often be performed in a single step. Once the raw biomass is impregnated with activating agents, both carbonization and activation occur simultaneously, streamlining the process.

However, chemical activation has environmental drawbacks. The chemical impregnation step requires subsequent neutralization and extensive washing to remove residual chemicals from the final product. These post‐treatment steps generate a substantial amount of chemical waste and require large quantities of water, adding to the environmental burden. This high consumption of chemicals and water, along with the handling and disposal of chemical waste, can pose challenges in terms of both sustainability and regulatory compliance.

In comparison, physical activation, which typically uses high temperatures and an activating gas (such as steam or CO_2_), is more environmentally friendly. Although it may require higher temperatures and longer processing times, physical activation avoids the need for chemical reagents and minimizes waste generation, making it a greener option for AC production.

## 4 Structural and Surface Properties Affecting CO_2_ Adsorption

**Figure 8:**
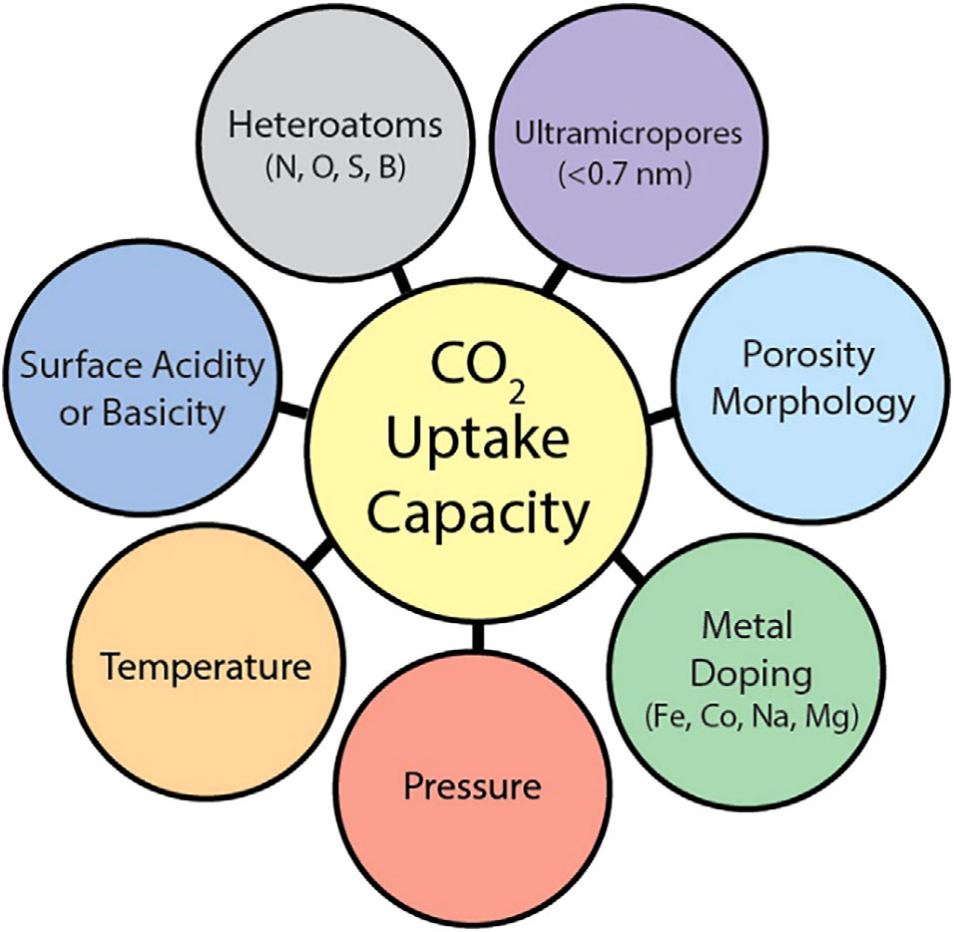
Overview of the factors influencing CO_2_ adsorption on biomass‐derived porous carbons.

The interaction between CO_2_ molecules and the adsorbent material is influenced by a range of physicochemical properties, such as porosity, surface area, and specific functional groups.^[^ ^204^ ^]^ These factors can affect adsorption capacity either directly or indirectly. For instance, synthesis conditions like activation temperature, pyrolysis temperature, acid treatment, gas flow rates during pyrolysis, and activation time can influence the specific surface area, which in turn impacts gas adsorption properties—an indirect effect.^[^ ^205^, ^206^, ^207^, ^208^ ^]^ In contrast, intrinsic factors such as micropore and ultramicropore volume, pore structure, presence of heteroatoms, O/N ratio, nitrogen types, functional groups, and surface acidity/basicity have a direct impact on adsorption. Additionally, external factors like temperature, gas pressure, moisture content, and gas composition also play a crucial role in determining the final gas adsorption capacity of the adsorbents.^[^ ^209^, ^210^, ^211^, ^212^, ^213^ ^]^ **Figure** **8** provides an overview of the parameters affecting CO_2_ uptake capacity, which will be discussed in detail in the following sections.

### 4.1 Role of Pore Size and Structure in Enhancing Carbon Capture Performance

As discussed earlier, a variety of physically and chemically ACs are employed for CO_2_ capture. Selective CO_2_ capture can be achieved through i) the molecular sieve effect, ii) interactions between CO_2_ molecules and the adsorbent surface, and iii) the combination of both effects. Consequently, the kinetic diameter and critical properties of the gas molecules are major factors in the adsorption process of CO_2_ from a gas mixture.^[^ ^214^ ^]^ **Table** **3** summarizes the critical and physical properties of gases commonly present in CO_2_‐containing mixtures.^[^ ^215^ ^]^ Given the critical role of molecular size and adsorption mechanisms in selective CO_2_ capture, the pore size and structure of AC play a fundamental role in determining adsorption efficiency, as explored in the following sections.

**Table 3:** Physical and critical properties of key gases in mixtures for carbon capture.^[^ ^215^ ^]^

Gas	Molecule diameter [nm]	Critical pressure [bar]	Critical temperature [K]
CO_2_	0.33	73.97	304.2
CH_4_	0.38	45.99	190.6
O_2_	0.346	50.36	154
N_2_	0.364	33.94	126.2
H_2_O	0.265	220.58	647.1

#### 4.1.1 Micropores

Micropores (pores with diameters less than 2 nm) are particularly effective in adsorbing CO_2_ due to their comparable size to actual CO_2_ molecules, which are ≈0.33 nm.^[^ ^216^, ^217^, ^218^ ^]^ This similarity in scale allows for strong interactions between the adsorbent surface and CO_2_ molecules, resulting in high adsorption capacities. The large surface area generated by the presence of micropores is crucial for capturing significant volumes of CO_2_.

Numerous studies have demonstrated that high CO_2_ adsorption capacity at 1 atm is closely linked to pores smaller than 0.8 nm in diameter, with pore sizes less than 0.5 nm being especially effective for CO_2_ capture at low partial pressures. A sharp increase in CO_2_ uptake at low relative pressures (P/Po < 0.1) is indicative of the material's predominantly microporous nature. The high CO_2_ capacity in these narrow micropores is due to the overlapping potential fields from adjacent pore walls. Additionally, a linear relationship is often observed between equilibrium adsorption capacity and micropore volume, indicating that the presence of narrow micropores significantly enhances CO_2_ adsorption^[^ ^219^, ^220^, ^221^ ^]^

These findings are supported by Grand Canonical Monte Carlo (GCMC) simulations.^[^ ^222^, ^223^, ^224^ ^]^ For example, Rehman and Park^[^ ^225^ ^]^ prepared a series of chitosan‐based microporous carbons featuring micropores of less than 1 nm. The sample, with an ultramicropore volume of 0.62 cm^3^ g^−1^, demonstrated a CO_2_ adsorption capacity of up to 6.36 mmol g^−1^ at 273 K. Similar findings by other researchers have reinforced the critical role of ultra‐micropores in enhancing CO_2_ adsorption capacity.^[^ ^226^, ^227^ ^]^ Interestingly, this trend does not apply to heteroatom‐doped carbons, such as nitrogen and sulfur‐doped carbons, where adsorption capacity depends on the content of heteroatoms like nitrogen in the form of amine, pyridinic, or pyrrolic functionalities, rather than on micropore volume and surface area.^[^ ^228^ ^]^

Controlling the size and geometry of micropore structures is essential yet challenging for preparing high‐performance carbon adsorbents for CO_2_ capture. Both physical and chemical activation processes play a critical role in developing microporous structures and enhancing the specific surface area necessary for effective CO_2_ adsorption. In particular, the treatment temperature is a crucial factor in achieving suitable pore sizes for CO_2_ adsorption.^[^ ^229^ ^]^ However, optimizing activation conditions is vital as higher temperatures increase porosity but reduce carbon yields due to violent volatilization.

Beyond conventional methods, innovative approaches like carbide‐derived carbons (CDCs) involve the selective etching of metal carbides with chlorine gas to control pore sizes precisely. By adjusting the chlorination temperature, a range of average pore sizes from less than 1 nm to greater than 10 nm can be achieved. For instance, Micro‐TiC‐CDC with a mean pore size of 0.66 nm exhibited a CO_2_ adsorption capacity of up to 7.1 mmol g^−1^ at 273 K and 1 bar.^[^ ^223^ ^]^

#### 4.1.2 Mesopores

Microporous carbon materials often suffer from high mass‐transfer resistance, resulting in a limited adsorption rate. To achieve rapid adsorption kinetics, it is essential to eliminate internal and external diffusion barriers. Creating a mesoporous structure (pores with diameters ranging from 2 to 50 nm) or constructing a hierarchical pore structure is crucial for promoting gas diffusion and mass transfer effectively.^[^ ^230^ ^]^ Additionally, mesopores significantly contribute to the overall pore volume of the adsorbent, enhancing its storage capacity. Template method is used for controlling the mesoporous structure of AC with specific structures. Based on the nature of the template used, this method can be categorized into hard‐template and soft‐template techniques.

The hard template method includes using a variety of nanomaterials such as salts,^[^ ^231^, ^232^ ^]^ geopolymers,^[^ ^233^ ^]^ SiO_2_,^[^ ^234^ ^]^ carbon species,^[^ ^235^ ^]^ and metal‐based^[^ ^236^ ^]^ species as templates due to their ease of preparation, large‐scale production, and high uniformity. The process involves three main steps: preparation of hard templates with specific pore structures, deposition of target precursors onto the template, and removal of the inner template.^[^ ^237^ ^]^ The soft template method is a versatile approach for creating mesopores, using templates such as macro‐ and microemulsions,^[^ ^238^ ^]^ micelles,^[^ ^239^ ^]^ vesicles,^[^ ^238^ ^]^ polymers,^[^ ^240^ ^]^ and biomolecular assemblies. Unlike solid templates, soft templates are typically in a liquid or gas form and exhibit high deformability, which eliminates the need for the complex and tedious template removal process.^[^ ^241^ ^]^

Triblock copolymers are among the most widely used soft templates for preparing ordered mesoporous carbons. For instance, a novel self‐assembly strategy based on benzoxazine chemistry was developed using a triblock copolymer composed of poly(ethylene oxide)–poly(propylene oxide)–poly(ethylene oxide) as the template. This method produced monolithic porous carbon with a hierarchical pore structure, demonstrating CO_2_ adsorption capacities of 4.9 mmol g^−1^ at 273 K and a CO_2_/N_2_ selectivity of 28 at room temperature.^[^ ^242^ ^]^ Different from block copolymers, liquid foam stabilized by solid particles has also been employed as a template to synthesize monolithic carbons, resulting in robust materials with interconnected macroporous and microporous networks. The materials demonstrated a CO_2_ absorption capacity of 2.62 mmol g^−1^ under static conditions of 1 bar and 298 K.^[^ ^243^ ^]^

#### 4.1.3 Macropores

Macropores (pores with diameters >50 nm) do not directly contribute to CO_2_ adsorption due to their large size. However, they improve the structural stability of the adsorbent, providing mechanical strength that helps maintain the integrity of the material during adsorption and regeneration cycles. Macropores also facilitate the transport of CO_2_ molecules to the smaller mesopores and micropores, ensuring efficient utilization of the adsorbent material.

A well‐connected pore structure ensures efficient transport pathways for CO_2_ molecules, reducing resistance to gas flow and enhancing the overall adsorption rate. This interconnected network of pores facilitates the smooth movement of CO_2_, minimizing diffusion limitations and allowing for faster adsorption and desorption cycles. In large‐scale packed bed columns, macropores also play a crucial role in reducing pressure drop across the bed by providing low‐resistance channels for gas flow. A well‐developed macroporous network improves permeability, preventing excessive pressure buildup, which is critical for maintaining energy‐efficient operation in industrial CO_2_ capture systems.^[^ ^244^, ^245^ ^]^ High pore connectivity ensures that CO_2_ molecules can quickly reach and interact with the active adsorption sites within the material, thereby optimizing the adsorbent's capacity and efficiency. **Table** **4** compares the physical properties of porous bio‐based carbonaceous adsorbents for CO_2_ capture.

**Table 4:** Comparison of physical properties of porous bio‐based carbonaceous adsorbents for CO_2_ capture.

Carbon precursor	Activating agent	S_BET_ [m^2^ g^−1^]	V_tot_ [cm^3^ g^−1^]	V_micro_ [cm^3^ g^−1^]	CO_2_ uptake [mmol g^−1^]	Adsorption P [bar] and T [°C]	Refs.
Sugar beet leaves	KOH	2777	1.86	1.41	19.70	20 bar and 25 °C	Park et al.^[^ ^101^ ^]^
Coffee grounds	KOH	548	0.25	0.20	8.50	1 bar and 25 °C	Serafin et al.^[^ ^246^ ^]^
Citrus aurantium leaves	ZnCl_2_ + CO_2_	937	0.57	0.30	8.40	1 bar and 0 °C	Balou et al.^[^ ^247^ ^]^
Grape marc	KOH	2473	1.09	0.72	6.20	1 bar and 0 °C	Ismail et al.^[^ ^248^ ^]^
Vine shoots	KOH	1439	0.67	0.50	6.10	1 bar and 0 °C	Manyà et al.^[^ ^249^ ^]^
Rice Husk	KOH + Chitosan	1495	0.786	0.447	5.83	1 bar and 0 °C	He et al.^[^ ^29^ ^]^
Walnut Shell	KOH	3225	1.99	0.21	5.40	1 bar and 25 °C	Rouzitalab et al.^[^ ^93^ ^]^
Date palm leaf	Pyrolysis	920	0.45	0.35	5.20	1 bar and 25 °C	Salem et al.^[^ ^250^ ^]^
Pine nutshell	KOH	1486	1.23	0.64	5.00	1 bar and 25 °C	Deng et al.^[^ ^99^ ^]^
Tobacco Stem	KOH	1922	0.92	0.79	4.84	1 bar and 25 °C	Ma et al.^[^ ^96^ ^]^
Coconut shell	Urea, KOH	1535	0.60	0.56	4.80	1 bar and 25 °C	Chen et al.^[^ ^251^ ^]^
Pomeloes	K_2_C_2_O_4_	1371	0.62	0.54	4.67	1 bar and 25 °C	Joshi et al.^[^ ^252^ ^]^
Microalgae	KOH	1940	0.82	0.76	4.50	1 bar and 25 °C	Sevilla et al.^[^ ^95^ ^]^
Shrimp shells	KOH	1405	0.72	0.60	4.20	1 bar and 25 °C	Yang et al.^[^ ^115^ ^]^
Coconut shell	KOH	1177	0.52	–	4.15	1 bar and 25 °C	Bai et al.^[^ ^164^ ^]^
Jujun grass	KOH	3144	1.56	1.23	4.10	1 bar and 25 °C	Coromina et al.^[^ ^253^ ^]^
Birch	Steam	688	0.49	0.35	4.06	1 bar and 0 °C	Kishibayev et al.^[^ ^105^ ^]^
Eucalyptus sawdust	KOH	2610	1.15	0.74	4.00	1 bar and 25 °C	Hirst et al.^[^ ^254^ ^]^
Coffee grounds	KOH	1525	0.77	0.54	3.82	1 bar and 25 °C	Wang et al.^[^ ^255^ ^]^
Terminalia arjuna seeds	H_3_PO_4_	1123	0.65	0.52	3.82	1 bar and 25 °C	Mallesh et al.^[^ ^256^ ^]^
Cellulose fibers	Steam	863	0.34	0.33	3.80	1 bar and 25 °C	Heo et al.^[^ ^114^ ^]^
Amazonian waste biomass	KOH	1624	0.90	0.38	3.67	1 bar and 25 °C	Serafin et al.^[^ ^257^ ^]^
Fern leaves	KOH	1016	0.611	0.539	3.58	1 bar and 25 °C	Serafin et al.^[^ ^168^ ^]^
Wood and food waste	KOH	841.	0.36	–	3.20	1 bar and 25 °C	Igalavithana et al.^[^ ^85^ ^]^
Persian Ironwood biomass	H_3_PO_4_	1802	1.11	0.83	3.02	1 bar and 30 °C	Nowruzi et al.^[^ ^258^ ^]^
Corncob	NH_3_	1154	0.57	–	2.81	1 bar and 25 °C	Geng et al.^[^ ^104^ ^]^
Camellia japonica	KOH	3537	1.85	1.21	2.80	1 bar and 25 °C	Coromina et al.^[^ ^253^ ^]^
Coffee Grounds	KOH	992	0.61	0.37	2.22	1 bar and 35 °C	Liu et al.^[^ ^84^ ^]^
Pine Sawdus	Steam	581	0.25	–	1.80	1 bar and 25 °C	Igalavithana et al.^[^ ^100^ ^]^
Olive Stones	H_3_PO_4_	1178	0.49	0.45	0.72	1 bar and 50 °C	Peredo‐Mancilla et al.^[^ ^103^ ^]^
Flawn grass	CO_2_	208	0.04	–	0.12	1 bar and 25 °C	Aslam et al.^[^ ^94^ ^]^

In addition to pore size and pore volume, the distribution of pore sizes within an adsorbent material is a critical factor influencing its effectiveness in capturing and storing CO_2_. When the adsorbent material has a uniform pore size distribution, it ensures consistent adsorption performance throughout the entire material. Additionally, since CO_2_ adsorption primarily occurs on the surfaces within the pores, a consistent pore size maximizes the surface area exposed to CO_2_ molecules, thereby enhancing the overall adsorption capacity of the material.

**Figure 9:**
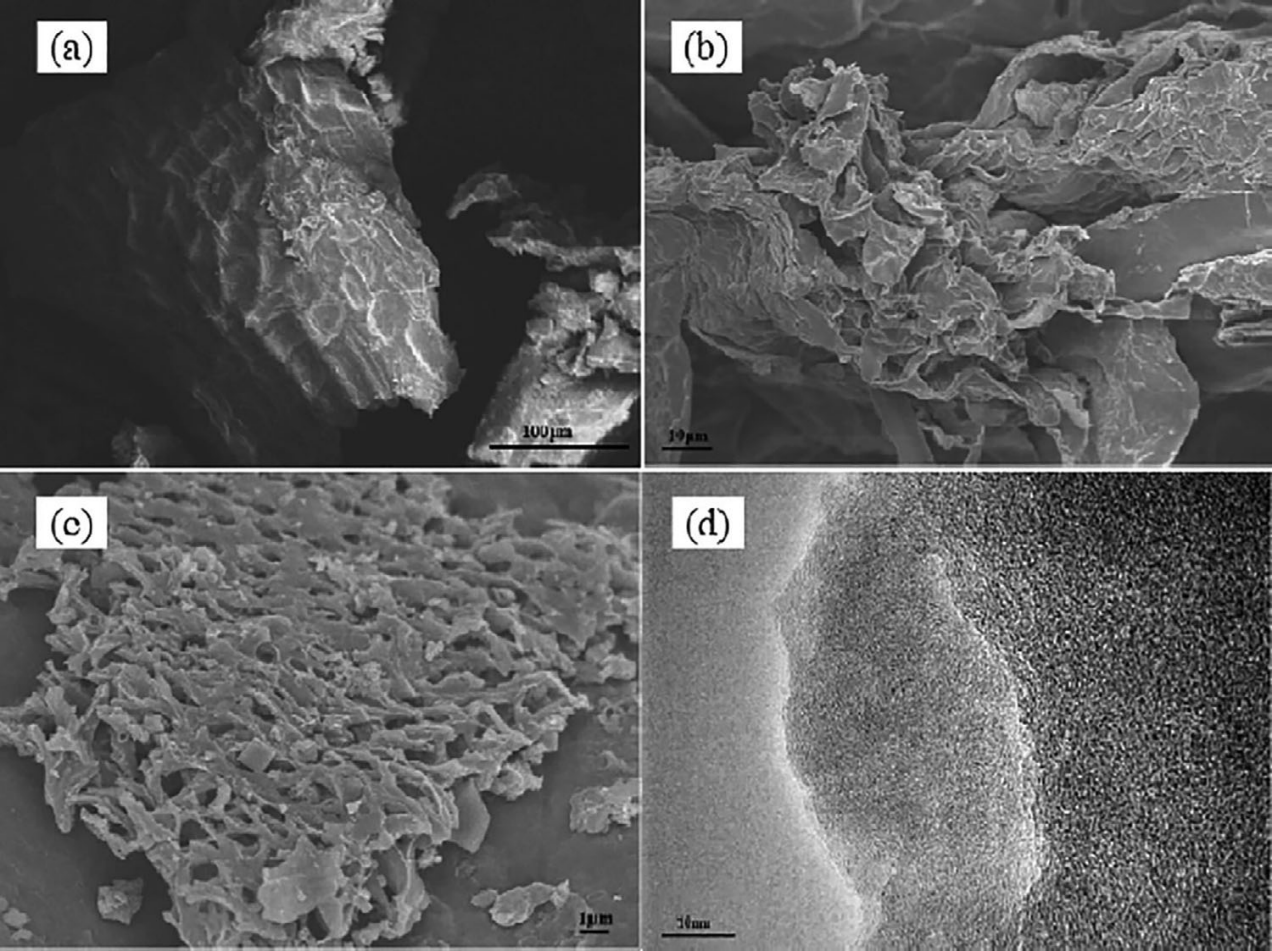
SEM images of a) shrimp shell, b) after carbonization without activating agent at 400 °C, c) after further activation using KOH at 700 °C. d) TEM image of the sample after activation with KOH at 700 °C. Reproduced with permissions from ACS publications.^[^ ^115^ ^]^

For example, fresh shrimp shells exhibit lattices with a relatively smooth surface (**Figure** **9**a). After carbonization without an activating agent, the surface develops many wrinkles, though distinct porous structures are not observed (Figure 9b). Further activation transforms the material, creating a carbon skeleton network characterized by irregular macropores (Figure 9c). This structure significantly enhances the specific surface area, facilitating CO_2_ penetration and providing numerous bonding sites. Figure 9d reveals that the activated sample, features a uniform distribution of amorphous worm‐like microparticles throughout the carbon framework, further contributing to its high surface area and adsorption capacity.^[^ ^115^ ^]^

### 4.2 Surface Chemistry and Functional Groups

Chemical modification of pristine AC aims to create more effective sorbents for CO_2_ capture. The functional groups, such as hydroxyl,^[^ ^259^ ^]^ carboxyl,^[^ ^260^ ^]^ and amine groups,^[^ ^261^ ^]^ play a crucial role in boosting the adsorption capacity through mechanisms like hydrogen bonding, electrostatic interactions, and chemical reactions. The surface chemistry of carbonaceous materials is influenced by the presence of heteroatoms such as hydrogen, oxygen, nitrogen, phosphorous, and sulfur.^[^ ^262^ ^]^ Understanding the importance of surface chemistry and functional groups is essential for optimizing the design and synthesis of bio‐based carbonaceous adsorbents, enabling more efficient and sustainable carbon capture solutions.

#### 4.2.1 Surface Acidity

The surface acidity of carbonaceous materials improves their affinity for alkaline gases, such as ammonia, through reactions with the acidic functional groups on the carbon's surface.^[^ ^263^ ^]^ This surface acidity is largely determined by oxygen‐containing functional groups that are either initially present in the precursors or introduced during the activation process. Bio‐based precursors used to produce AC contain oxygen atoms, which transform into oxygen functionalities during carbonization. Additionally, during the activation step, common activating agents such as KOH, NaOH, CO_2_, steam (H_2_O), and K_2_CO_3_ typically contain oxygen.^[^ ^264^ ^]^ Since activation occurs at high temperatures, the resulting carbon always retains some oxygen.^[^ ^221^ ^]^

Various oxidation techniques are employed to introduce oxygen‐containing functional groups, such as wet oxidation and dry oxidation techniques.^[^ ^265^ ^]^ Wet oxidation techniques using acids such as nitric acid,^[^ ^266^ ^]^ or oxidants such as hydrogen peroxide,^[^ ^267^ ^]^ and ammonium persulfate,^[^ ^268^ ^]^ are extensively used to introduce oxygen‐containing functional groups and typically occur at lower temperatures than dry oxidation.^[^ ^269^, ^270^ ^]^ For instance, Song et al.^[^ ^271^ ^]^ found that the CO_2_ adsorption capacity of commercial coconut shell AC modified with acetic acid increased by 30% compared to the unmodified sample. Dry oxidation involves exposing AC to oxidizing gases such as ozone,^[^ ^272^ ^]^ oxygen,^[^ ^273^ ^]^ and carbon dioxide^[^ ^274^ ^]^ and steam^[^ ^133^, ^275^ ^]^ at high temperatures above 970 K.

Although oxidation modification is a conventional method used to modify carbonaceous materials by introducing oxygen‐containing groups such as carboxyl, hydroxyl, phenolic, lactone, and carbonyl groups,^[^ ^276^, ^277^ ^]^ this method can have varying effects on the surface area and pore size of AC. Some studies indicated an increase in surface area due to additional porosity,^[^ ^278^, ^279^ ^]^ while others report a decrease in surface area and pore volume due to pore blockage by newly introduced groups.^[^ ^277^, ^280^, ^281^, ^282^, ^283^ ^]^ This variation depends on the oxidation conditions, such as oxidant concentration, temperature, time, as well as the nature of the AC used.^[^ ^265^ ^]^

**Figure 10:**
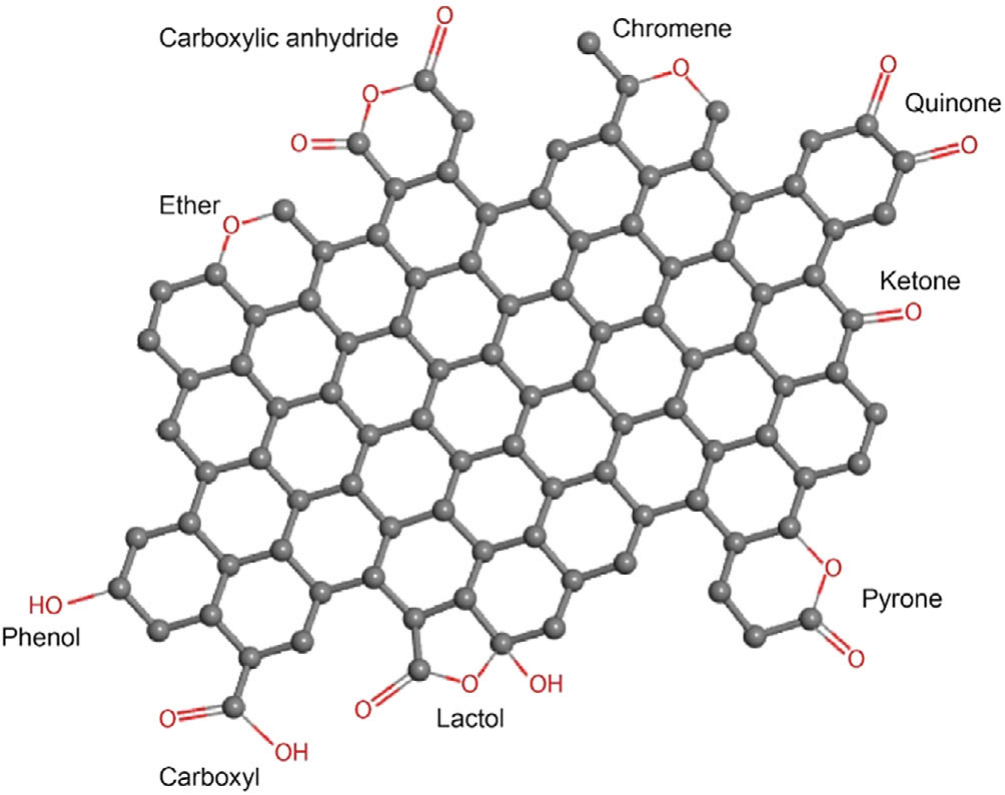
Oxygen‐based functional groups on the surface of oxidized AC.

The oxygen‐containing functional groups on carbon surfaces can be classified into two categories: acidic groups (including lactone, phenol, carboxyl, and lactol) and basic groups (such as pyrone, chromene, quinine, and diketone).^[^ ^284^ ^]^ **Figure** **10** illustrates the structure of several key oxygen‐containing groups present on oxidized carbon surfaces. While surfaces with acidic groups are not ideal for capturing acidic molecules like CO_2_, those with basic groups are suitable.^[^ ^285^ ^]^ Among the basic groups, pyrone‐based structures are the most studied, with their basicity attributed to the stability of their protonated form due to pi‐electron conjugation through the sp^2^ carbon plane.^[^ ^286^ ^]^ The stability of the carbon basal plane is also related to the presence of both sp^2^ and sp^3^ hybridized oxygen atoms. Tricyclic structures with pyrone‐like groups exhibit higher basicity compared to bicyclic ones, as indicated by their pKa values. Although chromene and quinine groups also contribute to basicity, detailed studies on these structures are lacking.^[^ ^205^ ^]^ Overall, the oxygen‐containing acidic groups introduced during modification are vital to the performance of AC, as they enhance the surface polarity and wettability, thereby improving the adsorption efficiency for polar organic compounds.^[^ ^287^, ^288^, ^289^ ^]^

#### 4.2.2 Surface Basicity

The basicity of AC is pivotal in determining the CO_2_ adsorption and CO_2_/N_2_ selectivity. Since CO_2_ is acidic, the presence of strong basic functional groups, heteroatoms such as nitrogen‐containing groups, or basic metal oxides, can offer lone pairs of electrons, thereby improving the chemisorption of CO_2_.^[^ ^290^ ^]^ The commonly used reducing agents include nitrogen, hydrogen, sodium hydroxide, potassium hydroxide, ammonia, and steam.^[^ ^124^, ^291^, ^292^ ^]^ The surface reduction of AC, particularly through the introduction of nitrogen functional groups and other reducing agents, can significantly improve the CO_2_ adsorption capacity of AC.^[^ ^54^, ^293^ ^]^

While these modifications enhance CO_2_ adsorption by increasing Lewis base activity and heteroatom content, they come with trade‐offs. The process typically requires high temperatures and substantial energy input to effectively incorporate functional groups. Additionally, it generates chemical by‐products and waste, posing environmental concerns. Therefore, optimizing modification techniques involves balancing adsorption efficiency with energy demands and sustainability considerations.

Li et al.^[^ ^294^ ^]^ investigated NaOH treatment of coconut shell‐derived carbon, and they observed increased pore volumes and specific surface areas of the particles, decreased the total oxygen functional groups, and improved adsorption capacity for hydrophobic gases such as o‐xylene. Although the study focused on o‐xylene, the principles of increased surface area and tailored surface chemistry can similarly enhance CO_2_ adsorption, even though CO_2_ may interact differently with surface functionalities compared to hydrophobic gases. In a related study, surface modification of AC using KOH and K_2_CO_3_ at temperatures ranging from 660 to 850 °C resulted in significantly improved textural properties, with a total pore volume reaching 1.53 cm^3^ g^−1^. CO_2_ adsorption measurements revealed that the modified carbons exhibited a higher capacity for CO_2_ capture, particularly within micropores sized between 0.3 and 0.8 nm, which were identified as the most effective for adsorption. The highest recorded CO_2_ adsorption capacity was 5.88 mmol g^−1^ at 0 °C and 1 bar for the carbon modified with KOH at 850 °C. Consequently, surface reduction modification, especially with KOH, markedly enhances the physical characteristics of AC, making it more effective for CO_2_ capture.^[^ ^295^ ^]^

#### 4.2.3 Metal Doping

Another important method to improve basicity and CO_2_ capacity is the introduction of basic metal oxide onto the carbon surface.^[^ ^296^ ^]^ This method involves introducing metal ions into the AC pore channels. These materials are reduced to simple substances or low‐valence ions, which improve the adsorption properties of AC. Commonly used metal ions include copper (Cu^2+^),^[^ ^297^ ^]^ cobalt (CO_2_^+^),^[^ ^298^ ^]^ nickel (Ni^2+^),^[^ ^299^ ^]^ magnesium  (Mg^2+^),^[^ ^300^ ^]^ calcium (Ca^2+^),^[^ ^301^ ^]^ iron (Fe^3+^),^[^ ^302^ ^]^ aluminum (Al^3+^),^[^ ^303^ ^]^ and silver (Ag^+^).^[^ ^304^ ^]^ These metal oxides are typically introduced by wet impregnation, wherein the carbon precursor is mixed with metal salt solutions and then subjected to carbonization or calcination at elevated temperatures. In particular, Fe^3^⁺ doping increases the surface basicity through the formation of iron oxides (e.g., Fe_2_O_3_, Fe_3_O_4_), which introduce electron‐rich surface sites. These oxides enhance Lewis basicity by donating electron density to acidic CO_2_ molecules, promoting stronger electrostatic and acid‐base interactions during adsorption. Additionally, Fe^3^⁺ species can facilitate redox interactions with CO_2_, further improving uptake capacity. This synergistic mechanism of increased basic functional groups and redox‐active centers explains the enhanced CO_2_ capture performance of Fe‐doped ACs.^[^ ^302^ ^]^

For example, pyrolyzing chromated copper arsenate‐treated wood at 700 °C yielded an AC with a CO_2_ adsorption capacity of 1.88 mmol g^−1^. The effect of metal oxide incorporation on the structure and CO_2_ adsorption capacity of AC is highly dependent on the metal loading.^[^ ^305^ ^]^ Finding the optimal loading is essential, as insufficient metal loading may not enhance the capacity, while excessive loading can block pores, leading to a reduction in surface area and pore volume. For instance, Hidayu et al.^[^ ^157^ ^]^ investigated AC derived from kernel shell and coconut shell, impregnated with various metal oxides such as barium oxide (BaO), magnesium oxide (MgO), copper oxide (CuO), titanium oxide (TiO_2_), and cerium oxide (CeO_2_). They found that BaO‐impregnated AC demonstrated the highest CO_2_ adsorption capacity, whereas MgO‐impregnated AC showed the lowest. The decrease in adsorption capacity for the MgO‐loaded AC was due to pore blockage caused by the metal oxide. In contrast, BaO impregnation enhanced the basicity of the AC, which strengthened the electrostatic interaction between CO_2_ and BaO, which facilitated the chemisorption of CO_2_.

In another study, various metals were doped onto walnut shell‐derived carbons through wet impregnation using metal nitrates. When comparing the CO_2_ adsorption efficiency of the impregnated metals at the same mass loading, their effectiveness can be ranked as follows: Mg > Al > Fe > Ni > Ca > unmodified biochar > Na.^[^ ^306^ ^]^ Similarly, cottonwood was doped with chlorides of magnesium (Mg), aluminum (Al), and iron (Fe). Among these samples, at comparable doping levels, the CO_2_ adsorption efficiency follows the order: Al > Fe > Mg.^[^ ^307^ ^]^ Overall, it has been shown that different metals have varying impacts on CO_2_ adsorption. Doping with metals such as Ca, Mg, Al, Fe, Zn, Cu, and Ni significantly enhances CO_2_ adsorption capacity, whereas metals like Pt, Au, Na, and Pd typically result in a reduced adsorption effect.^[^ ^308^ ^]^

At the optimal concentration, the chemisorption of CO_2_ on cationic metals such as Cu^2^⁺ and Zn^2^⁺ requires less adsorption energy because of the shorter distances between these cations and the CO_2_ molecules.^[^ ^309^ ^]^ Similarly, finding the optimal concentration of BaO in the wet impregnation method for incorporating BaO onto chitosan‐derived AC through thermal treatment under a nitrogen atmosphere is crucial. A 10% BaO loading was found to enhance surface properties and CO_2_ adsorption capacity but increasing the BaO concentration beyond this point led to a decrease in CO_2_ adsorption efficiency.^[^ ^310^ ^]^ Studies have demonstrated the effectiveness of loading potassium permanganate onto AC using an impregnation method. The modified AC showed a coral‐like structure with a significant increase in specific surface area, micropore volume, and adsorption capacity for gases such as CO_2_ and formaldehyde compared to unmodified AC.^[^ ^311^ ^]^

**Figure 11:**
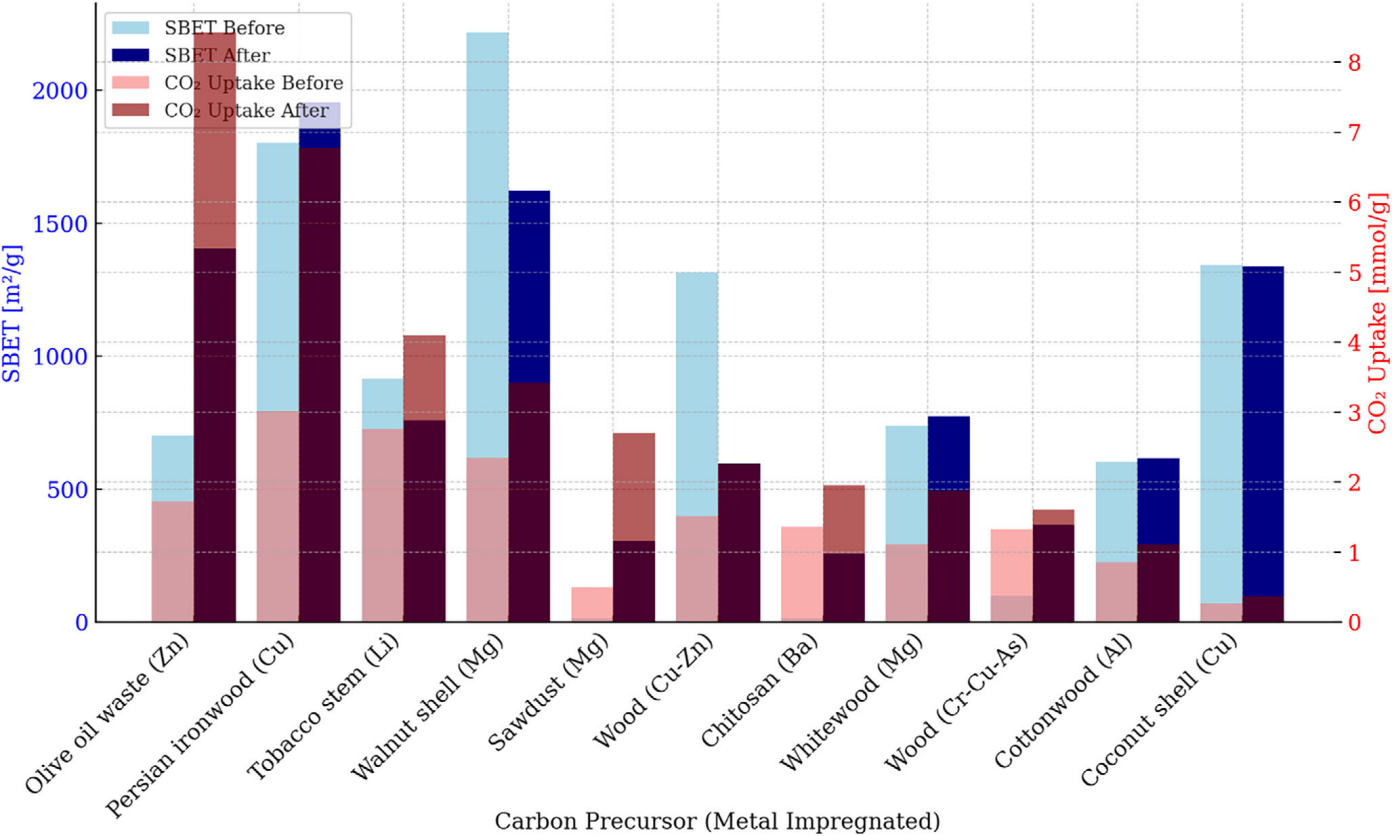
Changes in specific surface area (S_BET_) and CO_2_ uptake before and after metal impregnation for AC derived from olive oil waste (Zn),^[^ ^317^ ^]^ Persian ironwood (Cu),^[^ ^258^ ^]^ tobacco stem (Li),^[^ ^318^ ^]^ walnut Shell (Mg),^[^ ^319^ ^]^ sawdust (Mg),^[^ ^320^ ^]^ wood (Cu‐Zn),^[^ ^309^ ^]^ chitosan (Ba),^[^ ^310^ ^]^ whitewood (Mg),^[^ ^321^ ^]^ wood (Cr‐Cu‐As),^[^ ^305^ ^]^ cottonwood (Al)^[^ ^307^ ^]^ and, coconut Shell (Cu).^[^ ^322^ ^]^

Studies on the effect of metal loading on the carbon surface before and after activation revealed that introducing transition metal oxides like CoO, CrO_3_, and Co_3_O_4_ to AC prior to activation can catalyze the degasification process, resulting in the formation of both mesopores and micropores.^[^ ^312^, ^313^ ^]^ The development of wider mesopores allows for better dispersion of metal ions, while the formation of micropores facilitates the deposition of these ions on the surface, thereby enhancing the performance of the AC.^[^ ^314^, ^315^ ^]^ Conversely, loading metal ions onto carbon surface after the activation can adversely affect surface properties and CO_2_ adsorption capacity, mainly due to the pore blockage caused by metal ions impregnation.^[^ ^316^ ^]^ **Figure** **11** compares the structural and CO_2_ adsorption properties of bio‐based ACs doped with various metal ions.

#### 4.2.4 Heteroatom Doping

Another key strategy for enhancing the basicity and CO_2_ capacity is heteroatom doping,^[^ ^323^ ^]^ which involves changing the surface chemistry of AC by incorporating non‐metal elements such as nitrogen,^[^ ^324^, ^325^, ^326^, ^327^ ^]^ sulfur,^[^ ^328^, ^329^, ^330^, ^331^, ^332^ ^]^ fluorine,^[^ ^333^, ^334^, ^335^ ^]^ and phosphorus^[^ ^336^, ^337^, ^338^, ^339^ ^]^ into the carbon structure. This process enhances the porous carbon's properties, tailoring it for specific applications like gas adsorption and catalysis. In the following section, each heteroatom doping method will be discussed briefly, highlighting its unique contributions to the performance of AC.

Nitrogen doping introduces functional groups such as pyridine, pyrrole, amine, and graphitic nitrogen, which enhance interactions with acidic gases and polar pollutants. Doping carbonaceous materials with nitrogen can be achieved by different methods:

1. The first method involves synthesizing porous carbon and then introducing nitrogen by reacting it with a nitrogen‐containing agent. Common nitrogen‐containing activating agents for this method include ammonia,^[^ ^340^ ^]^ ammonium hydroxide,^[^ ^341^ ^]^ sodium amide,^[^ ^342^ ^]^ and acetonitrile.^[^ ^343^ ^]^ These agents serve both as nitrogen donors and activating agents, enhancing the porosity of the carbon surface.
2. The second method includes impregnation of pristine nanoporous carbon with nitrogen‐containing functionalities.^[^ ^344^ ^]^
3. The third method involves synthesizing a nitrogen‐containing but nonporous compound by carbonizing a nitrogen containing precursor such as melamine,^[^ ^345^ ^]^ gelatin,^[^ ^346^ ^]^ and chitosan.^[^ ^347^ ^]^ This is followed by introducing porosity through activation using agents like KOH, CO_2_, K_2_CO_3_, and steam.^[^ ^348^, ^349^ ^]^ Another approach to creating porosity in nonporous compounds is in situ templating. This can be achieved through hard templating, which uses inorganic templates such as SBA‐15^[^ ^350^ ^]^ MCM‐41^[^ ^351^ ^]^ MOFs, and zeolites,^[^ ^352^ ^]^ or through soft templating, which employs amphiphilic surfactants like P123^[^ ^353^, ^354^ ^]^ pluronic F127 (a triblock copolymer with a central block of polypropylene glycol flanked by polyethylene glycol blocks),^[^ ^355^, ^356^ ^]^ or cetrimonium bromide,^[^ ^357^, ^358^ ^]^ where the template does not need to be removed.

**Figure 12:**
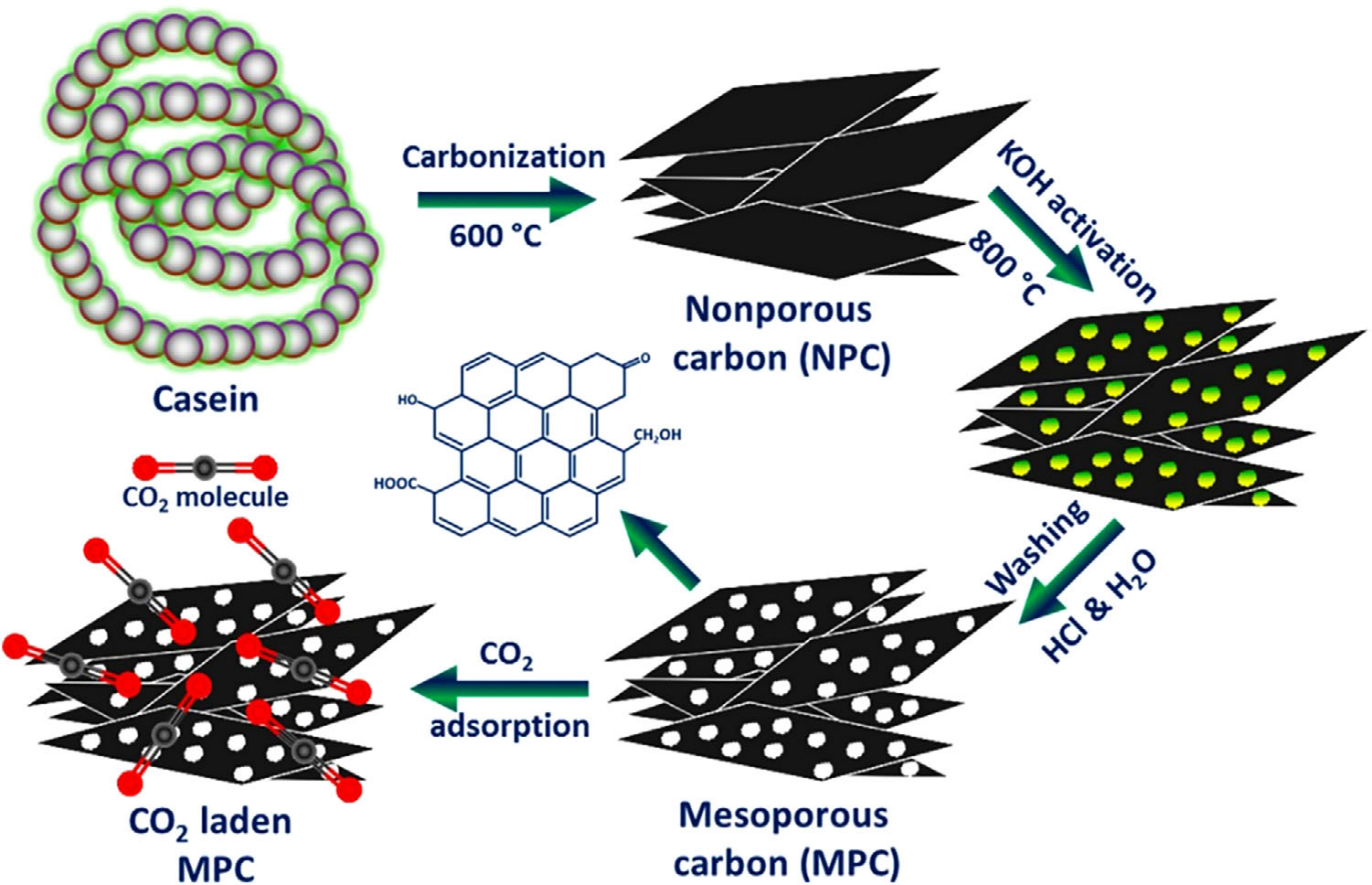
Schematic representation of the synthesis of mesoporous carbons from casein as a source of nitrogen and oxygen for heteroatom doing and their application in CO_2_ capture. Adopted with permission from the American Chemical Society.^[^ ^118^ ^]^

**Figure** **12** illustrates the synthesis of nitrogen and oxygen‐containing porous carbons by carbonizing a casein precursor and introducing porosity through KOH activation.^[^ ^118^ ^]^

Chitosan is a biomass that acts as both a carbon and nitrogen source. Fujiki et al.^[^ ^359^ ^]^ synthesized N‐doped AC using chitosan as the carbon and nitrogen precursor. The chitosan was combined with various alkali carbonates for activation and then carbonized. The AC produced with Rb_2_CO_3_ as the activating agent at a temperature of 600 °C achieved a high surface area of 1496 m^2^ g^−1^, a pore volume of 0.63 cm^3^ g^−1^, a nitrogen content of 3.15%, and a CO_2_ capture capacity of 4.88 mmol g^−1^. A recent study prepared AC from rice husk using carbonization and KOH activation, exploring the effects of impregnation ratios and activation temperatures. Optimally, the AC showed a BET surface area of 1495.52 m^2^ g^−1^, total pore volume of 0.786 cm^3^ g^−1^, and micropore volume of 0.447 cm^3^ g^−1^. Nitrogen doping with chitosan during activation incorporated N‐species, enhancing CO_2_ adsorption to 5.83 mmol g^−1^ at 273 K and 1 bar. The study established linear correlations between CO_2_ uptake, micropore volume, and N‐content. The isosteric heat of CO_2_ adsorption for modified AC averaged 30.21 kJ mol^−1^, significantly higher than the unmodified AC at 14.48 kJ mol^−1^. The adsorption behavior fit the Freundlich model and exhibited high selectivity for CO_2_ over N_2_, involving both physisorption and chemisorption.^[^ ^29^ ^]^

Nitrogen doping has been achieved through a multi‐step process involving the pyrolysis of microalgae powders, followed by urea treatment and subsequent microwave activation. This nitrogen doping process significantly enhanced the CO_2_ adsorption capacity of the ACs, with the nitrogen‐doped samples produced by microwave/KOH activation demonstrating higher CO_2_ uptake (4.21 mmol g^−1^) compared to those produced by thermal/KOH activation (3.44 mmol g^−1^). The increased nitrogen content, along with the developed pore structure from the microwave activation, contributed to the improved CO_2_ adsorption performance, highlighting the effectiveness of this doping and activation approach for CO_2_ separation applications.^[^ ^360^ ^]^ **Table** **5** provides a summary of the structural and adsorption characteristics of biomass‐derived nitrogen‐doped porous carbon materials.

**Table 5:** Overview of the structural and adsorption characteristics of biomass‐derived nitrogen‐doped porous carbon materials.

Carbon precursor	N source	N [%]	S_BET_ [m^2^ g^−1^]	V_tot_ [cm^3^ g^−1^]	CO_2_ uptake [mmol g^−1^]	Adsorption T [K], P [bar]	Refs.
Citrus aurantium leaves	Microalgae	12.51	937	0.57	8.43	273, 1	Balou et al.^[^ ^247^ ^]^
Black locust	Ammonia	7.21	2511	1.35	5.05	298, 1	Zhang et al.^[^ ^361^ ^]^
Glucose	Ethylenediamine	8.02	1793	0.87	5.01	298, 1	Ma et al.^[^ ^110^ ^]^
Glucose	Melamine	2.07	3247	3.09	4.95	298, 1	Rehman et al.^[^ ^111^ ^]^
Coconut shell	Urea	1.23	1535	0.96	4.80	298, 1	Chen et al.^[^ ^251^ ^]^
Sugarcane Bagasse	Urea	1.98	1113	0.51	4.80	298, 1	Han et al.^[^ ^362^ ^]^
Chitosan	Chitosan	3.49	1506	0.64	4.40	298, 1	Shao et al.^[^ ^363^ ^]^
Date sheets	Date sheets	0.26	2367	1.15	4.36	298, 1	Li et al.^[^ ^364^ ^]^
Rice husk	Chitosan	–	1496	0.78	4.20	298, 1	He et al.^[^ ^29^ ^]^
Shrimp shells	Shrimp Shells	2.16	1405	0.72	4.20	298, 1	Yang et al.^[^ ^115^ ^]^
Biomass tar	Biomass tar	3.03	1076	0.44	4.11	298, 1	Li et al.^[^ ^88^ ^]^
Poplar catkins	Poplar catkins	2.89	1455	0.68	4.05	298, 1	Chang et al.^[^ ^365^ ^]^
Hazelnut Shell	Sodium amide	1.98	1343	0.55	3.94	298, 1	Liu et al.^[^ ^366^ ^]^
Adenine and glucose	Adenine	1.34	3273	1.68	3.60	298, 1	Jiang et al.^[^ ^367^ ^]^
Eucalyptus wood	Ammonia	7.76	2079	1.29	3.22	303,1	Heidari et al.^[^ ^106^ ^]^
Oil‐tea seed shell	Sodium amide	1.48	2137	0.95	3.17	298, 1	Zhang et al.^[^ ^368^ ^]^
Waste wood	Cyanoguanidine	4.6	2541	1.52	2.86	298, 1	Shi et al.^[^ ^89^ ^]^
Tea seed	Melamine	3.41	1188	0.52	2.75	298, 1	Quan et al.^[^ ^369^ ^]^
carboxymethylcellulose	Potassium nitrate	5.73	2582	–	2.70	298, 1	Zhang et al.^[^ ^370^ ^]^
Bamboo waste	Urea	3.87	532	–	2.63	298, 1	Dilokekunakul et al.^[^ ^87^ ^]^
Casein	Casein	–	3617	1.79	2.30	298, 1	Sing et al.^[^ ^118^ ^]^

Apart from dry and wet techniques, plasma treatment has been used to enhance the surface properties of AC and to introduce functional groups.^[^ ^371^, ^372^, ^373^, ^374^, ^375^, ^376^, ^377^ ^]^ Plasma is an ionized gas composed of positive ions, negative ions, electrons and neutral particles. This method is advantageous due to its mild reaction conditions, safety, and low cost.^[^ ^287^ ^]^ Nitrogen plasma treatment can effectively enhance the CO_2_ adsorption capacity and selectivity of AC by simultaneously etching the surface to create ultramicropores and introducing nitrogen functional groups. This method achieves significant improvements with low energy consumption and short processing time.^[^ ^378^, ^379^ ^]^ However, plasma treatment mainly modifies the external surface and it does not affect the bulk material's performance.^[^ ^380^ ^]^

**Figure 13:**
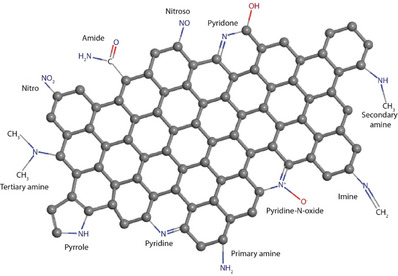
Nitrogen‐based functional groups on nitrogen doped carbon surface.

The effect of nitrogen content on the heat of CO_2_ adsorption have been widely examined in literature.^[^ ^381^, ^382^, ^383^, ^384^ ^]^ For nitrogen and sulfur co‐doped nanoporous carbon from algae, the heat of CO_2_ adsorption increased substantially with a lower nitrogen content. However, at higher nitrogen content, the heat of CO_2_ adsorption remains unchanged. This flattening of the curve suggests an energetically homogeneous nature of the adsorbent surface.^[^ ^228^ ^]^ A similar pattern is observed for carbons derived from lignin and subsequently activated with NH_3_.^[^ ^385^ ^]^ Different nitrogen‐containing functional groups present on the carbon surface are illustrated in **Figure** **13**.

Sulfur doping has been investigated for its potential to introduce functional groups such as thiols,^[^ ^386^ ^]^ sulfonic acids,^[^ ^387^ ^]^ sulfides,^[^ ^388^ ^]^ and thiophenes,^[^ ^389^ ^]^ which significantly improve CO_2_ capture performance. These groups increase the basicity of the carbon material, thereby improving surface interactions with acidic gases such as CO_2_ by strengthening dipole‐dipole interactions.^[^ ^390^ ^]^ The main effects of sulfur atom doping can be discussed in six key areas:^[^ ^391^ ^]^

- Increased adsorption sites: Doping with sulfur atoms increases the number of adsorption sites and improves the likelihood of CO_2_ molecule adsorption. Sulfur atoms’ chemical properties allow them to interact with CO_2_ molecules, thereby increasing adsorption capacity.
- Surface chemistry modification: Sulfur doping can alter the surface chemistry of porous carbon materials, introducing sulfur‐containing functional groups that introduce acidophilic sites, which enhance CO_2_ interactions with the material's surface.
- Improved charge transport: The electrical structure of carbonaceous materials can be modified by sulfur atom doping, contributing to improved charge transport capabilities that are beneficial for CO_2_ capture processes.
- Optimization of pore structure: Doping with sulfur can refine the pore structure of porous carbon materials, resulting in optimized pore volume and size, which can improve the selective adsorption of CO_2_.
- Enhanced chemical reactivity: Sulfur atom doping may enhance the surface activity of porous carbon materials, potentially enabling them to catalyze the conversion of CO_2_ into valuable compounds.
- Increased stability: Sulfur doping contributes to increased material stability, performance across multiple adsorption/desorption cycles.

For example, a study investigated sulfur‐doped ACs from polythiophene using a chemical activation method with potassium hydroxide. The resulting sulfur‐doped ACs showed high sulfur content and large specific surface areas, significantly enhancing adsorption capacity. The introduction of sulfur functional groups, particularly oxidized sulfur groups, was found to improve adsorption at low pressures due to increased binding energy between acetone molecules and the carbon surface.^[^ ^392^ ^]^ Recently, Bai et al.^[^ ^393^ ^]^ prepared sulfur‐enriched porous carbon using coconut shell and potassium thiosulfate as the carbon precursor and sulfur source, respectively. By optimizing pyrolysis temperatures, the resulting materials achieved an impressive surface area of up to 1924 m^2^ g^−1^ and a pore volume of 0.45 cm^3^ g^−1^. The optimal adsorbent demonstrated exceptional CO_2_ uptake capacities of 5.31 mmol g^−1^ at 0 °C and 3.59 mmol g^−1^ at 25 °C. Key factors influencing this performance included high sulfur content, narrow microporosity, and optimal pore size distribution. Additionally, the S‐doped carbons showed excellent CO_2_/N_2_ selectivity, strong cycling stability, and fast adsorption kinetics, marking them as promising candidates for efficient CO_2_ capture.

Beyond N and S‐doping, nitrogen and sulfur doping agents have broader applications that extend beyond merely enhancing heteroatom doping and CO_2_ adsorption. These agents can serve as greener alternatives to traditional activating agents like corrosive KOH. The corrosive nature of KOH can cause equipment corrosion, posing challenges for scale‐up applications. As a result, greener chemical agents such as sodium thiosulfate (Na_2_S_2_O_3_) and sodium amide (NaNH_2_) have gained attention due to their non‐corrosive properties, sustainability, cost‐effectiveness, and dual functionality as both heteroatom dopants and pore‐forming activating agents.^[^ ^368^, ^394^, ^395^, ^396^, ^397^, ^398^, ^399^, ^400^ ^]^ For example, Dai et al.^[^ ^401^ ^]^ demonstrated that sodium amide can introduce nitrogen species into oxygen‐containing carbon materials at low temperatures due to its strong nucleophilicity and basicity. This successfully improved the microporosity of mesoporous carbon through sodium amide activation, leading to enhanced CO_2_ adsorption performance. Additionally, employing solutions of sodium thiosulfate using a vacuum wet impregnation technique yielded S‐enriched carbon monoliths with outstanding dynamic CO_2_ adsorption capacities beyond 1 mmol g^−1^.^[^ ^400^ ^]^ Recently, researchers have investigated N, S co‐doped microporous carbon adsorbents using sodium thiosulfate and sodium amide. The synergistic effect of N and S co‐doping leads to a high CO_2_ adsorption capacity of 3.73 mmol g^−1^ and a maximum selectivity value of 7.80 for separating binary CO_2_/CH_4_ mixtures (10/90) under ambient conditions.^[^ ^402^ ^]^

While nitrogen and sulfur co‐doping have proven effective in enhancing CO_2_ adsorption and selectivity, another promising approach is fluorination. Fluorine, an element with higher electronegativity than boron and nitrogen, has an excess of valence electrons. The strong electronegativity of fluorine atoms on the carbon surface creates electronic vacancies, which in turn can improve CO_2_ adsorption performance.^[^ ^403^, ^404^ ^]^ The direct fluorination method via a gas‐phase reaction has gained significant attention due to its ability to achieve uniform modification, its short reaction time, high efficiency and low cost.^[^ ^405^ ^]^ This effectiveness is primarily due to the high reactivity of fluorine gas with carbon at room temperature.^[^ ^406^ ^]^ Fluorination introduces defects, changes surface properties, and enhances the total number of basic sites with different basicity levels on carbon materials’ surfaces.^[^ ^407^, ^408^, ^409^ ^]^ Additionally, direct fluorination can modify the nature of surface carbons, whether they are hydrophilic or hydrophobic.^[^ ^405^ ^]^ Therefore, fluorination can be used to control the properties of the carbon surface.^[^ ^405^ ^]^

For example, fluorinating carbon molecular sieves at atmospheric pressure and room temperature increased the fluorine content to 22.5%, which enhanced the CO_2_ adsorption capacity from 1.61 to 2.04 mmol g^−1^ at 25 °C. This modification also increased the pore volume, particularly for pores with an 8 Å diameter. The increased fluorine content affected the Lewis acid‐base interactions between CO_2_ and the fluorinated surface, and improved the CH_4_/CO_2_ gas separation behavior, resulting in a delayed breakthrough in the adsorption process.^[^ ^410^ ^]^ CO_2_ adsorption studies revealed that the extent of fluorination significantly impacted the activation energy barriers for CO_2_ diffusion, which decreased with higher fluorination levels.^[^ ^411^ ^]^

NF_3_ plasma‐treatment of AC is another effective way for F‐doping. Lim et al.^[^ ^412^ ^]^ used plasma discharge to induce micropore formation and increase the specific surface area, while also introducing fluorine functional groups. The synergistic effects of nitrogen and sulfur groups inherent in the carbon precursor, fluorine groups from NF_3_ treatment, and micropores generated by plasma etching significantly enhanced the CO_2_ adsorption capacity. Only after 60 seconds of NF_3_ plasma treatment, the yielded AC exhibited a 13% surface fluorine content, 14.5% increase in specific surface area, and a stable CO_2_ adsorption capacity of 4.55 mmol g^−1^ at 298 K and 1 bar over multiple cycles. Another novel method for achieving fluorine‐doped carbon involves the carbonization of a fluorine‐containing MOF, followed by chemical activation using KOH. This fluorination process results in the formation of numerous micropores and active sites on the carbon surface, leading to a large specific surface area of 1611 m^2^ g^−1^.^[^ ^413^ ^]^ A recent study explored an innovative method for creating fluorine‐doped porous carbon by using polytetrafluoroethylene (PTFE) as both a porogen and a fluorine source during carbonization, along with ball milling treatment. This approach resulted in an F‐doped porous carbon with a significant CO_2_ adsorption capacity of 4.1 mmol g^−1^ at 273 K and 1 bar. Additionally, the CO_2_ capture capacity remained stable after eight adsorption cycles, indicating no significant loss of performance and demonstrating the material's durability for CO_2_ capture applications.^[^ ^414^ ^]^

For phosphorus doping, groups such as phosphates, phosphonates and phosphites are added which improve surface acidity, thermal stability and binding affinity of AC. A study demonstrated that doping AC with phosphoric acid significantly enhanced the surface area to 1500 m^2^ g^−1^ and achieved a mesopore volume of 0.6 cm^3^ g^−1^. Increasing the impregnation ratio further enhanced the development of porosity, especially mesopores, due to the incorporation of phosphorus within the carbon structure. This process also improved the CO_2_ adsorption capacity, increasing it from 0.039 cm^3^ g^−1^ to 0.311 cm^3^ g^−1^.^[^ ^415^ ^]^ Wu et al.,^[^ ^338^ ^]^ synthesized a biomass‐derived phosphorus‐doped microporous carbon material using a hard template method, which involved preparing a precursor by dissolving sucrose in ultrapure water and mixing it with phosphoric acid and a microporous silica template. The mixture was then carbonized at 900 °C and treated with hydrofluoric acid to remove the template, yielding phosphorus‐doped carbon materials with different phosphorus content. This method allowed for the precise control of phosphorus incorporation, which significantly influenced the structural properties and CO_2_ adsorption performance of the materials. The phosphorus‐doped carbon with a phosphorus/carbon molar ratio of 0.075 showed the highest specific surface area of 802 m^2^ g^−1^ and the highest CO_2_ adsorption capacity of 63 mg g^−1^ at 30 °C and 50 mL min^−1^ CO_2_ flow rate. This enhanced performance is attributed to the improved microporosity and the presence of phosphorus‐containing functional groups that improve CO_2_ interaction with the carbon surface. Moreover, the adsorbent showed excellent stability, maintaining its CO_2_ adsorption capacity over 10 adsorption‐desorption cycles, highlighting its potential for practical applications in CO_2_ capture and storage.

Li et al.^[^ ^416^ ^]^ conducted a similar comprehensive study on the effects of doping carbon with nitrogen, sulfur, and phosphorus for the adsorption of CO_2_, CH_4_, and H_2_ gases. For phosphorus doping, resorcinol‐formaldehyde resin was used as the carbon precursor, with phosphoric acid serving as both the catalyst and the phosphorus source during the polymerization reaction. The resulting polymer was carbonized at 900 °C, and after the removal of the hard template by hydrofluoric acid, phosphorus‐doped carbon was obtained. This phosphorus‐doped carbon exhibited a CO_2_ adsorption capacity of 2.56 mmol g^−1^ at room temperature and 100 kPa, which was not significantly higher than that of the non‐doped carbon. The study attributed this to the acidic nature of the phosphorus groups, which are less favorable for CO_2_ capture compared to the basic nitrogen sites.

A novel approach to fabricating multifunctional carbon materials involves synthesizing a multifunctional polymer precursor, followed by activation to create porous carbon. For instance, a highly cross‐linked triazine polymer can be synthesized using dicyandiamide, triglycidyl isocyanurate, and phytic acid as nitrogen and phosphorus sources. After polymerization, the resulting material undergoes pre‐carbonization at 400 °C and subsequent activation with KOH at 700 °C. This activation process facilitates the incorporation of both phosphorus and nitrogen into the carbon structure, resulting in a high specific surface area of 1332 m^2^ g^−1^. The CO_2_ adsorption capacity is significantly enhanced, reaching 5.68 mmol g^−1^ at 5 bar and 298 K, due to the synergistic effect of nitrogen and phosphorus co‐doping. Phosphorus plays a crucial role in improving the structural properties, increasing overall porosity and surface area, which, combined with active nitrogen sites, contributes to the enhanced CO_2_ adsorption performance.^[^ ^417^ ^]^

Recently, quaternary‐doped porous carbons, incorporating nitrogen, sulfur, oxygen, and phosphorus, have garnered significant attention due to their enhanced properties.^[^ ^418^ ^]^ Wang et al.^[^ ^419^ ^]^ studied quaternary‐doped porous carbon microspheres, incorporating nitrogen, sulfur, oxygen, and phosphorus by one‐step pyrolysis of polyphosphazene at various temperatures from 750 °C to 950 °C. The sample pyrolyzed at 900° showed the highest CO_2_ uptake of 4.3 mmol g^−1^ at 0 °C and 760 mmHg, demonstrating the synergistic effect of ultramicroporosity and heteroatom doping. The samples exhibited a high concentration of ultramicropores (<0.7 nm), a subset of micropores (<2 nm), which fall within the nanopore size range but are distinguished by their particularly small dimensions. These ultramicropores are highly effective for CO_2_ capture, as their size closely matches the kinetic diameter of CO_2_ molecules (0.33 nm). The high CO_2_ adsorption capacity was closely dependent on both the microporosity and the content of heteroatoms in the carbon matrix.

Overall, heteroatom doping has demonstrated significant potential for enhancing CO_2_ capture performance in carbon‐based materials. Nitrogen doping consistently provides the most substantial improvements in CO_2_ adsorption by creating basic sites that interact favorably with CO_2_ molecules. Sulfur doping introduces sulfur‐containing groups that can interact with CO_2_, though its effectiveness is generally lower than that of nitrogen. However, sulfur also enhances the chemical stability and selectivity of carbon materials in the presence of moisture, which will be discussed in subsequent sections. Fluorine doping, due to its high electronegativity, tends to increase adsorption energy, but it can also reduce surface area and pore volume. As a result, optimizing the fluorine content is crucial to balancing its overall impact on CO_2_ capture. Phosphorus doping, particularly when combined with nitrogen, enhances CO_2_ uptake by improving the structural properties of the carbon material. The most effective doping strategies often involve co‐doping with multiple heteroatoms to harness synergistic effects on porosity and surface chemistry. Therefore, future research should focus on fine‐tuning doping levels and exploring co‐doping approaches to maximize the CO_2_ capture performance and selectivity of carbon‐based adsorbents.

### 4.3 Selectivity Improvement in Humid Conditions

The gas adsorption capacity of AC can be limited by several factors, such as the competition between water and the target adsorbate, the physicochemical properties of the gases, and a slow mass transfer rate during the adsorption process.^[^ ^420^, ^421^ ^]^ A critical factor in evaluating gas adsorption performance is selectivity in a gas mixture. Selectivity is defined as the mole ratio of CO_2_ adsorbed on the adsorbent to the moles in the feed stream that remain unadsorbed at breakthrough time. The removal of CO_2_ from gas mixtures is immensely important in various industries, including CO_2_/N_2_, CO_2_/H_2_O, CO_2_/O_2_, and CO_2_/H_2_ in pre‐combustion, post‐combustion, air purification, and biogas upgrading processes.

Generally, when the micropore size of an adsorbent is close to the kinetic diameter of the adsorbate, the van der Waals potential fields generated by the pore walls overlap, resulting in increased adsorption energy.^[^ ^422^ ^]^ Therefore, the diameter of the target adsorbate molecules should be similar to the micropore diameter of AC to enhance adsorption performance, particularly for small molecules. In the case of CO_2_ adsorption under atmospheric pressure and temperature, micropores narrower than 1 nm are ideal because they help retain CO_2_ molecules within the pore walls.^[^ ^95^, ^423^, ^424^ ^]^ These smaller pores are highly selective for CO_2_ in N_2_/CO_2_ mixtures, as they prevent N_2_ from entering the pores.^[^ ^425^ ^]^ Conversely, pore sizes greater than 1 nm are not suitable for CO_2_ capture at low pressures because they do not facilitate the dense packing of CO_2_ molecules.^[^ ^426^ ^]^ When it comes to CO_2_/H_2_O selectivity, it is challenging to prevent water adsorption by tuning micropores, as the size of water molecules is smaller compared to CO_2_.

A recent study investigated the adsorption characteristics of CO_2_/H_2_O and N_2_/H_2_O on AC, activated alumina, and zeolites 3A and 13X. The results showed that the breakthrough time for both CO_2_/H_2_O and N_2_/H_2_O decreases with increasing flow rates and temperatures, but the breakthrough time for N_2_ is typically shorter than for CO_2_ due to weaker adsorption interactions with AC. Notably, increasing the steam mole fraction from 1% to 2% initially extends the adsorption saturation time for CO_2_ in AC. This is because a water film forms, enhancing CO_2_ capture through stronger hydrogen bonding interactions. However, at higher steam concentrations, water competes with CO_2_ for adsorption sites, thereby reducing the overall capacity. Initially, AC demonstrates higher selectivity for CO_2_ over H_2_O and lower selectivity for N_2_ compared to CO_2_, and the presence of water vapor further reduces N_2_ adsorption due to competitive adsorption. Adsorption in AC primarily relies on its non‐polar nature and van der Waals forces, which are weaker than the ionic or electrostatic interactions found in zeolites. Consequently, AC is less effective at adsorbing water compared to zeolites. Therefore, AC's resilience to moisture and cost‐effectiveness makes it a viable option for CO_2_ capture applications. However, further research is needed to modify AC to improve its hydrophilicity or to amine impregnation, which could potentially enhance its performance for CO_2_ capture in humid conditions.^[^ ^427^ ^]^

**Figure 14:**
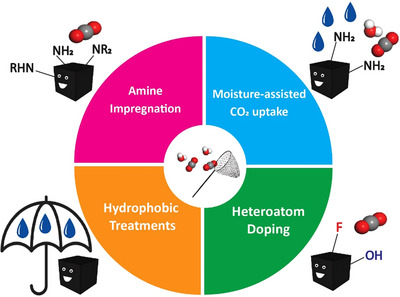
Several techniques are used to improve CO_2_ capture in humid environments, including: a) amine impregnation to boost CO_2_ chemisorption, b) moisture‐assisted CO_2_ uptake where water presence increases material's CO_2_ adsorption capacity such as using amines, c) heteroatom doping to enhance CO_2_ uptake capacity especially in presence of moisture, d) hydrophobic modifications to prevent sorbent deactivation.

Overall, the CO_2_ adsorption capacity of carbon materials decreases in the presence of humidity, which is commonly found in flue gas. This reduction occurs because water molecules compete with CO_2_ for adsorption sites due to the presence of hydrophilic polar oxygen or nitrogen groups on carbon surfaces. These groups can form hydrogen bonds with water vapor, increasing the surface's affinity for moisture.^[^ ^205^ ^]^ To overcome these limitations and enhance selectivity, especially in the presence of water, it is necessary to modify AC using various physical and chemical techniques, including surface oxidation and reduction, surface loading of materials, and low‐temperature plasma treatments. Given that water is an unavoidable by‐product in many industrial processes, adsorbents need to be highly hydrophobic to effectively remove moisture before targeting other impurities. The most crucial aspect is to consider the properties of the target compound and select the most appropriate modifications to optimize the selectivity of AC for the intended application.^[^ ^287^ ^]^ To tackle these challenges, a range of innovative material designs and novel techniques have been utilized, resulting in remarkable outcomes. Key approaches include i) incorporating heteroatoms to boost CO_2_ capacity and increase the material's moisture stability, ii) amine impregnation to enhance CO_2_ uptake in humid environments, iii) leveraging water to significantly improve CO_2_ chemisorption, and iv) adjusting the hydrophilicity of the adsorbent and its water wettability.^[^ ^11^ ^]^ **Figure** **14** illustrates various techniques for enhancing CO_2_ capture in humid conditions.

In terms of chemical properties, the surface of AC can be modified with various functional groups, many of which contain oxygen and increase its acidic nature, thereby enhancing its hydrophilicity. This increased hydrophilicity results in the adsorption of hydrophilic molecules, including water vapor. For example, a study has shown the impact of nitrogen doping on water adsorption, revealing that nitrogen‐doped carbon exhibited greater water adsorption capacities compared to non‐doped carbons.^[^ ^428^ ^]^ Numerous studies have proposed various theories and models for water vapor adsorption. One such model explains vapor adsorption on AC through the growth of three‐dimensional water clusters and networks. Hydrophilic functional groups, such as amino groups and many oxygen‐containing groups, play a fatal role in vapor adsorption at very low vapor pressures. As water clusters form and expand within the pore structure, the amount of vapor adsorbed increases significantly.^[^ ^429^, ^430^, ^431^, ^432^ ^]^

Conversely, if the AC surface is hydrophobic, it more effectively adsorbs hydrophobic gases. There are several methods to make the surface of AC hydrophobic, including grafting hydrophobic polymers, such as impregnation with polydimethylsiloxane (PDMS). For instance, modifying walnut‐derived AC with PDMS introduced hydrophobic Si–O–Si groups onto its surface. This modification significantly improved the carbon‐based gas adsorption under humid conditions compared to unmodified AC. As a result, the breakthrough time and saturated adsorption capacity under 90% humidity improved by 65% and 65.25%, respectively, demonstrating improved hydrophobicity.^[^ ^433^ ^]^

A recent study showed the development of hydrophobic AC through impregnation in PDMS, achieving a contact angle of 134° with water droplets, indicative of significant hydrophobicity. The hydrophobic AC demonstrated superior performance under humid conditions, with only a 0.3% weight increase when exposed to wet air, compared to 12% for commercial AC. Additionally, CO_2_ adsorption capacity of the hydrophobic AC was 0.05 mmol g^−1^ at 200 °C under steam at 1 atm, while the commercial AC's capacity decreased from 0.091 to 0.009 mmol g^−1^.^[^ ^434^ ^]^

While previous studies have shown that the presence of water negatively impacts CO_2_ adsorption performance in both MOFs and zeolites, the effect of moisture on CO_2_ adsorption in ACs is still a topic of debate. Some research indicates that CO_2_ uptake can increase in the presence of moisture due to several mechanisms: i) the formation of CO_2_ clathrates within the carbon pores at high pressure,^[^ ^435^, ^436^, ^437^ ^]^ ii) the formation of carbamates on the adsorbent surface,^[^ ^438^, ^439^ ^]^ and iii) the potential dissolution of CO_2_ in moisture, although there is no experimental evidence supporting the latter. Additionally, when the carbon surface contains amine groups, CO_2_ adsorption is significantly affected by humidity. In fact, for most amine‐impregnated ACs, the presence of water can enhance CO_2_ adsorption.^[^ ^214^ ^]^ Impregnation is typically performed using low molecular weight amines, such as diethanolamine (DEA), methyl diethanolamine (MDEA), and tetraethylene pentamine (TEPA).^[^ ^440^ ^]^ The choice of impregnant depends on various factors, including the size and molecular weight of the amine, the dimensions of the adsorbate, the pore size distribution of the adsorbent, and regeneration conditions.^[^ ^441^, ^442^ ^]^

For example, in AC impregnated with TEPA, the presence of 10 wt.% water vapor promotes CO_2_ uptake because bicarbonate forms instead of carbamates, which occur under dry conditions.^[^ ^443^, ^444^ ^]^ Another study found that modifying AC with sterically hindered amines, such as 2‐amino‐2‐methyl‐1‐propanol, significantly improves CO_2_ uptake in dry conditions, even though it reduces surface area due to pore blockage. Additionally, the presence of moisture aids in forming bicarbonate instead of carbamate, which further enhances CO_2_ uptake in humid conditions.^[^ ^445^ ^]^ Similarly, it was found that introducing 1 vol.% of water can improve the CO_2_ adsorption capacity by 56%, although the rate of CO_2_ uptake declines as the water content exceeds 1 vol.%.^[^ ^446^ ^]^ These findings were also observed in other studies, but without additional explanation.^[^ ^447^ ^]^

Conversely, there are instances where moisture decreases CO_2_ uptake capacity but increases breakthrough and saturated capacities in amine‐modified ACs. This is due to the formation of a water film on the adsorbent surface when the water concentration exceeds 5 vol.%, which hinders interactions between CO_2_ molecules and amine sites, thereby constraining the rates of adsorption.^[^ ^448^ ^]^ Xu et al.^[^ ^449^ ^]^ found that CO_2_ uptake improves significantly as the water vapor concentration in the feed stream rises, provided it remains lower than the CO_2_ concentration. However, when the water vapor concentration exceeds that of CO_2_, the excess water inhibits CO_2_ adsorption.

**Figure 15:**
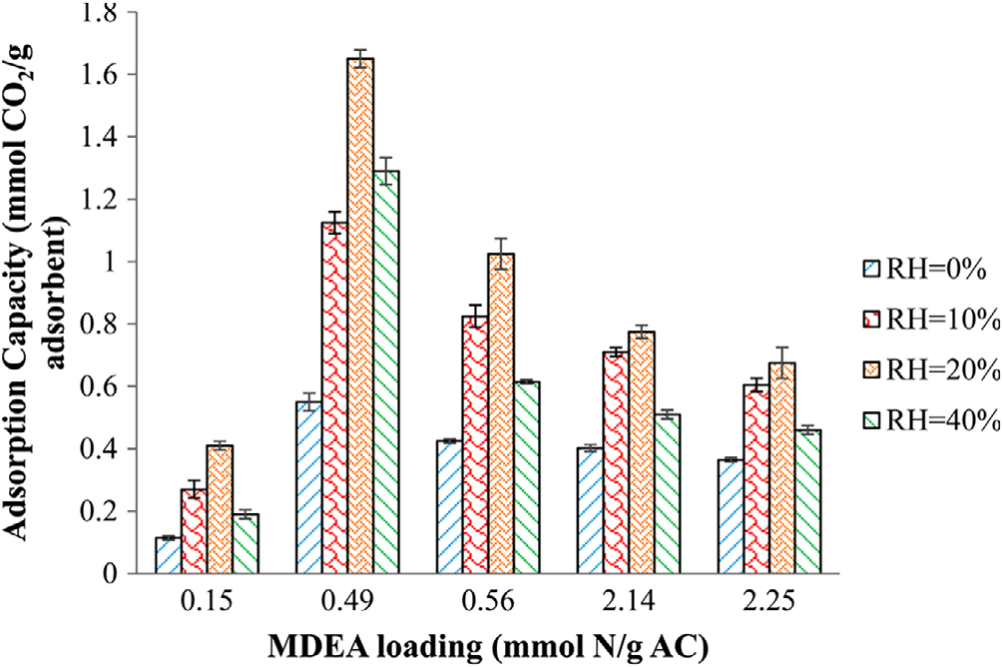
Moisture effect on CO_2_ adsorption capacity of AC impregnated with MDEA. Adsorption tests were conducted with 20% CO_2_ in N_2_ at 50 °C and atmospheric pressure. Error bars show the standard deviation of the mean from two data sets. Reproduced with permissions from the American Chemical Society.^[^ ^442^ ^]^

**Figure** **15** shows the effect of relative humidity (RH) on the CO_2_ adsorption capacity of MDEA‐impregnated AC. The CO_2_ adsorption capacity initially improves as RH increases to 20%, but it decreases beyond this point due to the relative ratios of water vapor and CO_2_ in the feed. At low RH, the CO_2_ capacity increases because water catalyzes the interaction between CO_2_ and amine groups and stabilizes carbonate‐like species through hydrogen bonding. However, when the feed is saturated, excess water vapor competes with CO_2_ for active sites, reducing the adsorption capacity.^[^ ^442^ ^]^ These findings make the effects of moisture on CO_2_ adsorption in ACs a subject of ongoing debate. Moreover, while many studies have examined the impact of water on adsorption capacity and offered possible explanations, a comprehensive assessment of the adsorption process in the presence of water vapor is still needed. **Table** **6** provides a summary of selected studies highlighting the diverse effects of moisture on the performance of bio‐based AC sorbents, emphasizing the need for further systematic investigation.

**Table 6:** Summary of selected studies on the performance of bio‐based AC sorbents for CO_2_ capture under humid conditions.

Modification strategy	Sorbent type	Humidity conditions	CO_2_ adsorption effect	Observations	Refs.
Amine impregnation (MDEA)	Bio‐based AC	0–40% RH	↑ up to 20% RH, then ↓	Water promotes CO_2_‐amine interaction at low RH; competitive adsorption at higher RH	Gholidoust et al.^[^ ^442^ ^]^
Amine impregnation (TEPA)	Biomass‐derived AC	10 wt% H_2_O	↑ CO_2_ capacity	Forms bicarbonates instead of carbamates; moisture enhances chemisorption	Wang et al.^[^ ^443^ ^]^
N‐doping	N‐doped AC	∼60% RH	↑ H_2_O adsorption	Enhances hydrophilicity and water uptake, affecting CO_2_ selectivity	Horikawa et al.^[^ ^428^ ^]^
PDMS treatment	Walnut‐derived AC	90% RH	↑ Breakthrough time (↑65%)	Surface modified with Si–O–Si hydrophobic groups resists water uptake	Li et al.^[^ ^433^ ^]^
Hydrophobic polymer grafting	Commercial AC	Steam atmosphere (1 atm)	↓ CO_2_ loss under steam	Hydrophobic AC adsorbs less water; retains capacity at 200 °C	Hao et al.^[^ ^434^ ^]^

## 5 Adsorption Isotherms and Kinetics: Mechanisms, Models, and Thermodynamic Insights for Bio‐Based AC

Adsorption isotherms describe the adsorption of a substance to a solid surface from an aqueous or gas phase under isothermal conditions, which represents the equilibrium state when the rate of adsorption is equal to the rate of desorption. These isotherms provide the understanding of thermodynamic insight into the adsorption mechanism, surface properties, and the degree of affinity of the adsorbents.

Among all the isotherm models, the Langmuir isotherm was the widely applied one and first proposed in 1916 to represent the gas–solid adsorption onto AC.^[^ ^450^ ^]^ This model assumes ideal monolayer chemisorption of molecules on a smooth surface with a finite number of localized sites, which are identical and have no lateral interaction or molecule rearrangement. ^455–457^The adsorbed molecules are regarded as rigid particles with a fixed volume.^[^ ^451^ ^]^ Langmuir isotherm could be understood as a homogeneous adsorption process, and each molecule represents constant enthalpies and adsorption activation energy.^[^ ^452^ ^]^

The Langmuir isotherm is derived from the equilibrium adsorption reaction of gas molecules to the substrate surface as Equation 13:^[^ ^450^, ^453^ ^]^

(13) $$C_{e}+S_{e}\overset{k_{-1}}{\underset{k_{1}}{\to }}C_{e}S_{e}$$

where C_e_ is the equilibrium concentration of molecules (mg L^−1^) in the gas phase, S_e_ is the equilibrium empty site concentration (mg g^−1^) on the surface of the substrate, and C_e_S_e_ is the equilibrium concentration of adsorbed molecules (mg g^−1^) on substrate surface.

At equilibrium:

(14) $$k_{1}\left[C_{e}\right]\left[S_{e}\right]=k_{-1}\left[C_{e}S_{e}\right]$$

Defining substrate surface coverage as θ_s_ (Equation 15), S_e_ could be expressed as Equation (16).

(15) $$\theta_{s}=\frac{\left[C_{e}S_{e}\right]}{\left[S_{e}\right]+\left[C_{e}S_{e}\right]}$$

(16) $$\left[S_{e}\right]=\left(1-\theta_{s}\right)\left(\left[S_{e}\right]+\left[C_{e}S_{e}\right]\right)$$

Equation (14) can then be rearranged as Equation (17) with surface coverage:

(17) $$k_{1}\left[C_{e}\right]\left(1-\theta_{s}\right)=k_{-1}\theta_{s}$$

The equilibrium constant *K*_e_ is defined as:

(18) $$K_{e}=\frac{k_{1}}{k_{-1}}=\frac{\left[C_{e}S_{e}\right]}{\left[C_{e}\right]\left[S_{e}\right]}$$

Equation (17) could be further rearranged (Equation 19) and solved for θ_s_ as Equation (20):

(19) $$K_{e}C_{e}=\left(1+K_{e}C_{e}\right)\theta_{s}$$

(20) $$\theta_{s}=\frac{K_{e}C_{e}}{1+K_{e}C_{e}}=\frac{q_{e}}{Q_{max}}$$

where *q*_e_ (mg g^−1^ o.d. fiber) is the concentration of molecules adsorbed at equilibrium and *Q*_max_ (mg·g^−1^ o.d. fiber) is the maximum adsorption capacity. The equation can be further linearized as:

(21) $$\frac{C_{e}}{q_{e}}=\frac{1}{K_{e}Q_{\max}}+\frac{C_{e}}{Q_{\max}}$$

Plotting *C*_e_ */q*_e_ versus *C*_e_ enables calculation of maximum adsorption capacity, *Q*_max_, from the slope and equilibrium constant *K*_e_ from the intercept. From Equation 20, when *C*_e_ is large, *θ*_s_ is approximately equal to 1, representing full coverage of substrate; while, when *C*_e_ is small, *θ*_s_ approaches zero, suggesting limited adsorption and surface coverage.^[^ ^453^ ^]^

Another widely two‐parameter model, Freundlich model, was developed to represent multi‐layer adsorption process at the equilibrium state on a non‐ideal or multi‐site (heterogeneous) surface with adsorption energies having a heterogeneous distribution.^[^ ^454^ ^]^ But compared with Langmuir model, which is a model with theoretical parameters of physical meanings, Freundlich model is developed more as an empirical mathematical model with insufficient physical representations.^[^ ^452^, ^455^ ^]^

The rate equation for Freundlich isotherm is defined as:

(22) $$q_{e}=kC_{e}^{\frac{1}{n}}$$

where *k* is rate constant (g mg^−1^ min^−1^), *q*_e_ is the concentration of molecules (mg g^−1^) adsorbed at equilibrium state, *n* is the Freundlich constant, and *C*_e_ is the concentration of molecules (mg·L^−1^) in the aqueous or gas phase.

Taking the logarithm of both sides allows the equation to be linearized:

(23) $$\log q_{e}=\log k+\frac{1}{n}\log C_{e}$$

The plot of *logq*_e_ as a function of *logC*_e_ yields slope of 1/*n* and intercept of *logk*. The Freundlich constant, the “*n*” value, calculated by the slope, represents the degree of adsorption. When “*n*” is within the range of 1–10, it suggests that the adsorption is physical, multilayer, and favorable.^[^ ^161^, ^179^, ^456^ ^]^

Both Langmuir and Freundlich models are two‐parameter model, which means two parameters needs to be determined for the model fitting. However, in some cases, the isotherm fittings are similar with difficulties to determine a specific adsorption mechanism. Some three‐parameter models were proposed for a better fitting of adsorption data, such as Redlich–Peterson and Tóth models,^[^ ^457^, ^458^ ^]^ however, notes should be taken that more parameters usually lead to better fittings with less physical meaning and require larger quantities of data. Some commonly used isotherm models are summarized in **Table** **7**.

**Table 7:** Summary of the commonly used adsorption isotherm models, their linear form (non‐linear regression in Langmuir–Freundlich model), and physical understanding. Note: *q*_e_ is the concentration of molecules (mg g^−1^) adsorbed at equilibrium state, *C*_e_ is the equilibrium concentration of molecules (mg L^−1^) in the aqueous or gas phase, *q*_e_ (mg g^−1^ o.d. fiber) is the concentration of molecules adsorbed at equilibrium.

Isotherm	Linear forms	Parameters	Physical understanding	Refs.
Two‐parameter isotherms				
Freundlich	$\log q_{e}=\log k+\frac{1}{n}\log C_{e}$	*k*: rate constant (g mL^−1^ min^−1^) *n*: Freundlich constant	Multi‐layer adsorption on a non‐ideal or multi‐site (heterogeneous) surface.	Freundlich,^[^ ^459^ ^]^ 1906
Langmuir	$\frac{C_{e}}{q_{e}}=\frac{1}{K_{e}Q_{max}}+\frac{C_{e}}{Q_{max}}$	*Q_max_*: maximum adsorption capacity (mg.g^−1^) *K_e_*: equilibrium constant.	Monolayer chemisorption on an ideal or homogeneous surface with a finite number of localized sites.	Langmuir,^[^ ^450^ ^]^ 1916
Three‐Parameter Isotherms				
Langmuir‐Freundlich	$q_{e}=\frac{q_{MLF}{\left(K_{LF}C_{e}\right)}^{MLF}}{1+{\left(K_{LF}C_{e}\right)}^{MLF}}$	*q_MLF_*: maximum adsorption capacity (mg.g^−1^) *K_LF_*: equilibrium constant for heterogeneous solid *MLF*: heterogeneity parameter between 0 and 1	Reduces to Freundlich isotherm at low *q_e_* ​ and to Langmuir isotherm at high *q_e_* ​.	Koble and Corrigan,^[^ ^460^ ^]^ 1952
Redlich‐Peterson	$\ln\frac{C_{e}}{q_{e}}=\beta \ln C_{e}-\ln A$	*A*: Redlich–Peterson constant (L·g^−1^) β: exponent between 0 and 1	Combines Langmuir and Freundlich models; reduces to Langmuir isotherm when β = 1.	Redlich and Peterson,^[^ ^457^ ^]^ 1959
Tóth	$\ln\frac{q_{e}^{n}}{q_{m}^{n}-q_{e}^{n}}=n\ln K+n\ln C_{e}$	*K_T_*: Tóth constant (mg g^−1^) *n*: Tóth isotherm constant (mg g^−1^)	Reduces to Langmuir isotherm when *n* = 1; describes multilayer adsorption for non‐unity *n*.	Tóth,^[^ ^461^ ^]^ 2002

Thermodynamic parameters (the Gibbs energy change (Δ*G*), the enthalpy change (Δ*H*), and the entropy change (Δ*S*)) could be further determined by the Van't Hoff equation from the adsorption isotherm constants at equilibrium state and offer physical understanding of the thermodynamic properties of the CO_2_ adsorption (physical or chemical adsorption), and the affinity of CO_2_ to the carbon surface.^[^ ^161^, ^462^ ^]^

Enthalpy change (Δ*H*) is the internal energy change between reactants and products in a chemical reaction under constant pressure. The entropy change (Δ*S*) represents the adsorbate formation at the solid/liquid interface with high randomness (ΔS > 0, a dissociative mechanism) or low randomness (ΔS < 0, an associative mechanism).^[^ ^462^ ^]^ The Gibbs energy change (Δ*G*) is applied to determine the degree of spontaneity, and usually a negative value of ΔG indicates a favorable and spontaneous adsorption.^[^ ^455^ ^]^ The Gibbs energy change is determined from Equation (24), and the equilibrium constant *K*_C_ is the constant of various adsorption isotherms or the partition coefficient.^[^ ^462^ ^]^ The enthalpy change and the entropy change are calculated from the slope and intercept of a linear regression of *lnK*_C_ against *1/T* (Equation 26).

By the thermodynamic laws:

(24) $$\Delta G^{0}=-RTlnK_{C}$$

According to the relationship of Δ*G* ^0^ to Δ*H* ^0^ and Δ*S* ^0^:

(25) $$\Delta G^{0}=\Delta H^{0}-T\Delta S^{0}$$

Substituting Equation (24) into Equation (25) to obtain the van't Hoff equation:

(26) $$lnK_{C}=\frac{-\Delta H^{0}}{RT}+\frac{\Delta S^{0}}{R}$$

where *R* is the ideal gas constant (8.314 J mol^−1^ K^−1^), *T* is the absolute temperature (K).

The isosteric heat (*Q*_st_) is a measure of the surface affinity to CO_2_ reflecting the interaction between the adsorbate and the surface of the adsorbent by using the Clausius–Clapeyron equation.^[^ ^98^, ^179^ ^]^ The heat of adsorption is calculated by a linear plot of *ln P* versus *1/T* with the slope of *Q*_st_ (Equations 27 and 28).^[^ ^179^, ^463^ ^]^

(27) $$Q_{st}=-\Delta H$$

(28) $$\ln\left(P\right)=-\frac{Q_{st}}{R}\frac{1}{T}+C$$

where *Q*_st_ is the isosteric heat (kJ mol^−1^), Δ*H* is the adsorption enthalpy (kJ mol^−1^), *P* is the pressure (kPa), *R* is the ideal gas constant (8.314 J mol^−1^ K^−1^), and *T* is the absolute temperature (K).

Adsorption kinetics describe the variation of the amount adsorbed as a function of residence time, where adsorption rates of molecules are related to the collision rate. Pseudo‐first‐order and pseudo‐second‐order are the most commonly used kinetic models to describe adsorption of molecules or chemicals to substrate surface.^[^ ^464^, ^465^, ^466^, ^467^, ^468^ ^]^ The derivation of equations follow.

Pseudo‐first‐order adsorption kinetics is described by:

(29) $$\frac{dq_{t}}{dt}=k\left(q_{e}-q_{t}\right)$$

where *k* is the rate constant (g mg^−1^ min^−1^), *q*_e_ is the concentration of molecules (mg g^−1^) adsorbed at the equilibrium, same as in Equation (32*), and *q*_t_ is the concentration of molecules (mg g^−1^) adsorbed at any time, *t* (min).

Separating variables yields:

(30) $$\frac{dq_{t}}{q_{e}-q_{t}}=kdt$$

Integrating using the boundary conditions:

(31) $$t=0\;q_{t}=0$$

(32) $$t=t\;q_{t}=q_{t}$$

(33) $$\ln\left(q_{e}-q_{t}\right)=-kt+\ln q_{e}$$

Plotting ln*(q*_e_ *− q*_t_) as a function of t will yield a line with slope of *k* and intercept of *lnq*_e_.

The rate equation for pseudo‐second‐order adsorption kinetics:

(34) $$\frac{dq_{t}}{dt}=k{\left(q_{e}-q_{t}\right)}^{2}$$

where *k* is the rate constant (g mg^−1^ min^−1^), *q*_e_ is the concentration of molecules (mg g^−1^) adsorbed at equilibrium state, same as in Equation (32*), and *q*_t_ is the concentration of molecules adsorbed (mg g^−1^) at any time, *t* (min).

Separating variables:

(35) $$\frac{dq_{t}}{{\left(q_{e}-q_{t}\right)}^{2}}=kdt$$

Integrating using boundary conditions:

(36) $$t=0\;q_{t}=0$$

(37) $$t=t\;q_{t}=q_{t}$$

(38) $$\frac{1}{q_{e}-q_{t}}-\frac{1}{q_{e}}=kt$$

Equation (38) can be re‐arranged as:

(39) $$\frac{t}{q_{t}}=\frac{1}{k{\left(q_{e}\right)}^{2}}+\frac{t}{q_{e}}$$

Plotting *t/q*_t_ as a function of *t* will yield a line with slope *1/q*_e_ and intercept of *1/k(q*_e_).^[^ ^2^ ^]^

Though with rapid development of artificial intelligence (AI) and machine learning (ML) models in recent years, the classic adsorption kinetics and isotherms are still widely used in CO_2_ capture to gain insight into the physical mechanisms and compare AC performance as gold standards.

The CO_2_ adsorption behavior on bio‐based AC is predominantly governed by physical adsorption mechanisms. Several adsorption isotherm models have been applied to evaluate CO_2_ uptake characteristics, as summarized in **Table** **8**. The Freundlich model was the most fitted one, indicating a multi‐layer adsorption process for CO_2_.^[^ ^29^, ^161^, ^179^, ^258^ ^]^ The constant *n* of the Freundlich model is usually larger than 1, suggesting a heterogeneous surface condition.^[^ ^161^ ^]^ The k parameter in the Freundlich model is higher, and more favorable for CO_2_ desorption at higher temperature with nitrogen doping.^[^ ^161^ ^]^ A similar Freundlich adsorption isotherms were reported by He et al.^[^ ^29^ ^]^ (2021) with an adsorption capacity of 5.83 mmol g^−1^ utilizing rice husk for carbonization with KOH activation. The total surface area is 1495.52 m^2^ g^−1^ and the total pore volume is 0.786 cm^3^ g^−1^.^[^ ^29^ ^]^ Another Freundlich adsorption isotherm was reported by Nowrouzi et al.^[^ ^258^ ^]^ with Persian ironwood carbonization with single and binary mixed‐metal oxides and H_3_PO_4_ activations, and a high 6.78 mmol g^−1^ CO_2_ adsorption capacity was achieved. Peredo‐Mancilla et al.^[^ ^103^ ^]^ reported the Langmuir models, including the two‐parameter model and modified Langmuir model for CO_2_ adsorption, indicating a monolayer adsorption with limited adsorption sites on the surface of the carbon. The authors compared the adsorption competition between CO_2_ and CH_4_ and reported a favored adsorption capacity for CO_2_ on the porous carbon material derived from olive stones and activated by H_3_PO_4_, water, and CO_2_.^[^ ^103^ ^]^ The chemically‐activated AC showed a higher adsorption capacity, and a linear relationship between the micropore volume and the adsorption capacity of both CH_4_ and CO_2_ has been reported.^[^ ^103^ ^]^

**Table 8:** Summary of CO_2_ capture capacity, adsorption isotherms, and kinetic models by the biomass‐derived AC.

Carbon precursor	Activation method	CO_2_ capacity [mmol g^−1^] at 1 bar	Isotherms	Kinetic model	Refs.
Bamboo shoot shells	Chemical (urea and K_2_CO_3_)	7.52	Freundlich		Wenjun Wu et al.^[^ ^161^ ^]^
Tannic acid	Chemical (KOH)	7.03	Redlich–Peterson, Langmuir, Freundlich, and Tóth		Yujia Zhang et al.^[^ ^179^ ^]^
Persian ironwood	Chemical (H_3_PO_4_)	6.78	Freundlich		Mohsen Nowrouzi et al.^[^ ^258^ ^]^
Olive stones	Chemical (H_3_PO_4_) and Physical (H_2_O and CO_2_)	6.52	Modified Langmuir		Deneb Peredo‐Mancilla et al.^[^ ^103^ ^]^
Rice husk	Chemical (KOH)	5.83	Freundlich		He Song et al.^[^ ^29^ ^]^
Pine sawdust	Chemical (KOH and ZnCl_2_)	5.79	Toth		Catarina Helena Pimentel et al.^[^ ^98^ ^]^
Subabul wood	Chemical (H_3_PO_4_)	4.51		Pseudo‐first‐order	Dosali Mallesh et al.^[^ ^107^ ^]^
Pecan nutshell	Chemical (H_3_PO_4_)	4.1	Freundlich		Gabriela Dur´an‐Jim´enez et al.^[^ ^174^ ^]^
Sewage sludge and leucaena wood		1.2	Intraparticle diffusion	Pseudo‐second‐order	Yu‐Fong Huang et al.^[^ ^86^ ^]^

Some three‐parameter isotherm models were investigated for the CO_2_ capture of bio‐based AC, such as the Redlich–Peterson isotherm and Tóth isotherm at both high and low pressures, indicating a multilayered adsorption to heterogeneous surfaces.^[^ ^179^ ^]^ The “n” value in Tóth model ranges from 0.3 to 2.6, describing the lateral interactions among molecules and the adsorbent heterogeneity. All CO_2_ adsorption was well fitted for the two‐parameter isotherm models (Freundlich and Langmuir) and three‐parameter isotherm models (Redlich–Peterson and Tóth).^[^ ^179^ ^]^

Pimentel et al.^[^ ^98^ ^]^ studied the biochar and ACs from Pinus radiata sawdust with chemical activation (KOH or ZnCl_2_). The heat of adsorption is relatively constant and low (14–27 kJ mol^−1^) as a function of the amount of CO_2_ absorbed, indicating a low surface heterogeneity. The low heat of adsorption also represents a physical adsorption with a low regeneration energy requirement. The increase in isosteric heat as a function of CO_2_ adsorption capacity could be related to the reinforced intermolecular interaction between the CO_2_ and ACs, suggesting a multilayer adsorption of CO_2_. However, the decreasing trend of the isosteric heat indicates the heterogeneous surface and strong interactions of CO_2_ with the AC surface, with negligible intermolecular interaction among CO2.^[^ ^469^ ^]^

Wu et al.^[^ ^161^ ^]^ studied the adsorption of CO_2_ by bamboo shoot shell‐based AC, from the low value of Δ*H* (←16 kJ mol^−1^), the adsorption is regarded as an exothermic physisorption with a strong interaction between the adsorbent and the adsorbate.^[^ ^470^ ^]^ The negative value of ΔS suggests the multi‐layer adsorption or interactions between CO_2_ molecules and adsorbents. The negative value of ΔG indicates that the CO_2_ capture is spontaneous without external energy supply.^[^ ^161^ ^]^

Both pseudo‐first order and pseudo‐second‐order kinetic models are studied for CO_2_ capture. Huang et al.^[^ ^86^ ^]^ proposed a pseudo‐second‐order kinetic model with sewage sludge and leucaena wood carbonization by microwave heating, and a 53 mg g^−1^ of carbon adsorption capacity was demonstrated with intraparticle diffusion kinetic model fitting isotherms.^[^ ^86^ ^]^ Mallesh et al.^[^ ^107^ ^]^ reported the pseudo‐first order kinetics of CO_2_ adsorption for carbon adsorbent and demonstrated a desorption process with a stable recyclability. The activation energy is evaluated to be 260.0 cal mol^−1^ and the pre‐exponential factor is 24.5, suggesting the physical adsorption of CO_2_ with the relatively weak attraction forces.^[^ ^107^ ^]^

In summary, CO_2_ adsorption to biomass‐derived ACs is an exothermic, spontaneous, multi‐layer physisorption to heterogeneous surfaces with low external energy supply. Multiple isotherm models and kinetic models could fit the adsorption process depending on the trade‐off between interactions of CO_2_ with the AC surface and the intermolecular interaction among CO_2_ molecules.

### 5.1 Regeneration Techniques

The regeneration mechanism is a critical factor in selecting an appropriate adsorbent material, as it directly influences key performance indicators such as working capacity, CO_2_ purity, recovery efficiency, and energy consumption. The choice of regeneration method is primarily determined by the physical characteristics of the adsorbent and the nature of the adsorption process, which together affect the shape of the CO_2_ desorption isotherm and the associated thermal behavior. In line with these considerations, various regeneration techniques have been developed to suit different material properties and operating conditions. These include pressure swing adsorption (PSA),^[^ ^471^ ^]^ vacuum swing adsorption (VSA),^[^ ^472^ ^]^ temperature swing adsorption (TSA),^[^ ^473^ ^]^ electric swing adsorption (ESA),^[^ ^474^ ^]^ and hybrid approaches. Of these, PSA and VSA have achieved the most widespread commercial adoption, while methods such as TSA and ESA remain at various stages of research, development, and demonstration.^[^ ^18^ ^]^ The selection of an optimal regeneration strategy thus depends not only on the compatibility with the adsorbent material but also on factors such as scalability, energy efficiency, and the technological maturity of the system.

#### 5.1.1 PSA

The process involves I) increasing the bed pressure from an initial level (p_1_) to a target pressure (p_2_), II) performing isobaric adsorption until breakthrough while capturing less‐adsorbed components, (III) depressurizing the bed, typically to ambient conditions, and IV) desorbing strongly adsorbed species using a countercurrent flow of an inert gas. PSA is used in both pre‐combustion CO_2_ capture (such as hydrogen production from natural gas)^[^ ^475^, ^476^ ^]^ and post‐combustion applications.^[^ ^477^ ^]^ PSA can achieve CO_2_ purities of up to 99% with separation efficiencies around 90%.^[^ ^478^ ^]^ However, PSA systems typically require a larger physical footprint compared to absorption‐based methods, making them less efficient in terms of space and cost. Ongoing research aims to improve the energy efficiency of PSA, as the process involves complex valve networks and high‐cost compressors.^[^ ^479^ ^]^

#### 5.1.2 VSA

This process is a variant of PSA, where adsorbent regeneration is achieved by lowering the pressure to ≈0.05 atm to enable CO_2_ desorption. A notable advantage of VSA is its ability to produce CO_2_ with purity levels of up to 99%.^[^ ^480^ ^]^

#### 5.1.3 TSA

This process relies on heating the adsorbent bed to trigger desorption by breaking the adsorbent–adsorbate bonds. While this method enables the recovery of high‐purity CO_2_ and allows for the potential use of waste heat,^[^ ^481^ ^]^ it also has drawbacks. The need for significant thermal input increases energy costs, and the required cooling step after desorption extends cycle times, reducing overall system efficiency. Compared to PSA, TSA generally involves longer regeneration times due to the low thermal conductivity of packed beds.^[^ ^482^ ^]^ However, it benefits from simpler system design and lower electrical power requirements.^[^ ^483^ ^]^

#### 5.1.4 ESA

This technique regenerates the adsorbent by applying a low‐voltage electric current, which heats the bed via the Joule effect and enables rapid CO_2_ desorption.^[^ ^484^ ^]^ While ESA shares similarities with TSA, it differs significantly in terms of productivity, as electric heating is faster and does not rely on a diluent, making it more efficient at concentrating non‐condensable gases.^[^ ^485^ ^]^ One of ESA's main advantages is its short regeneration time and high energy efficiency, as heat is delivered directly to the adsorbent. However, ESA is less suitable for applications involving large gas volumes, where PSA may be more effective.^[^ ^479^ ^]^ Despite this, ESA is an emerging technology with strong potential to reduce CO_2_ capture costs from flue gas. AC is commonly used in ESA due to its favorable conductivity, which stems from its graphitic structure. Current research also explores composite materials designed to enhance CO_2_ adsorption performance.^[^ ^486^ ^]^

## 6 AI‐Driven Innovations in Carbon Capture

Over the past two decades, the application of computer‐aided numerical simulations across various fields has increased significantly. In particular, machine learning (ML techniques offer promising solutions for developing processes and materials for carbon capture applications and have become a focus of extensive research.^[^ ^487^, ^488^, ^489^ ^]^ In carbon capture, ML has been applied at both the RandD lab scale and the industrial scale, including in the use of solvent‐based post‐combustion capture,^[^ ^490^ ^]^ ionic liquids,^[^ ^491^ ^]^ membranes,^[^ ^492^ ^]^ and adsorbents.^[^ ^493^ ^]^

AI‐driven CO_2_ capture technology plays a vital role in optimizing and enhancing the efficiency of CO_2_ capture processes by fine‐tuning parameters such as pressure, temperature, flow rates, and chemical reactions.^[^ ^494^ ^]^ AI enables rapid prediction of the thermodynamic properties of CO_2_ in solutions^[^ ^487^, ^488^, ^495^ ^]^ and supports extensive monitoring systems for modeling atmospheric CO_2_ capture.^[^ ^496^ ^]^ ML offers highly accurate predictive models for CO_2_ capture, leveraging the vast amounts of operational data generated by large‐scale CO_2_ capture plants, such as TMC Mongstad in Norway and BD3 SaskPower in Canada. This data can be effectively used to enhance and optimize CO_2_ capture processes, leading to improved efficiency and performance.^[^ ^497^, ^498^, ^499^ ^]^ Specifically, AI has been utilized in carbon capture processes for the prediction and simulation of physical and chemical properties, such as partial pressure, solubility, compressibility, and mass transfer, as well as for modeling the entire CO_2_ capture process.

Several review papers have explored the applications of ML in pre‐combustion,^[^ ^500^ ^]^ oxy‐fuel combustion, post‐combustion (both adsorption and absorption),^[^ ^501^ ^]^ as well as CO_2_ transportation, storage, utilization,^[^ ^502^ ^]^ and conversion.^[^ ^503^ ^]^ These studies have highlighted a strong correlation between advanced ML models and experimental data, demonstrating the effectiveness of AI‐driven carbon capture.^[^ ^504^ ^]^ Recent research has also introduced algorithms designed to evaluate both the benefits and drawbacks of AI in carbon capture. These algorithms not only quantify the CO_2_ emissions generated during model training but also estimate the CO_2_ reductions achieved when the models are applied.^[^ ^505^ ^]^

**Figure 16:**
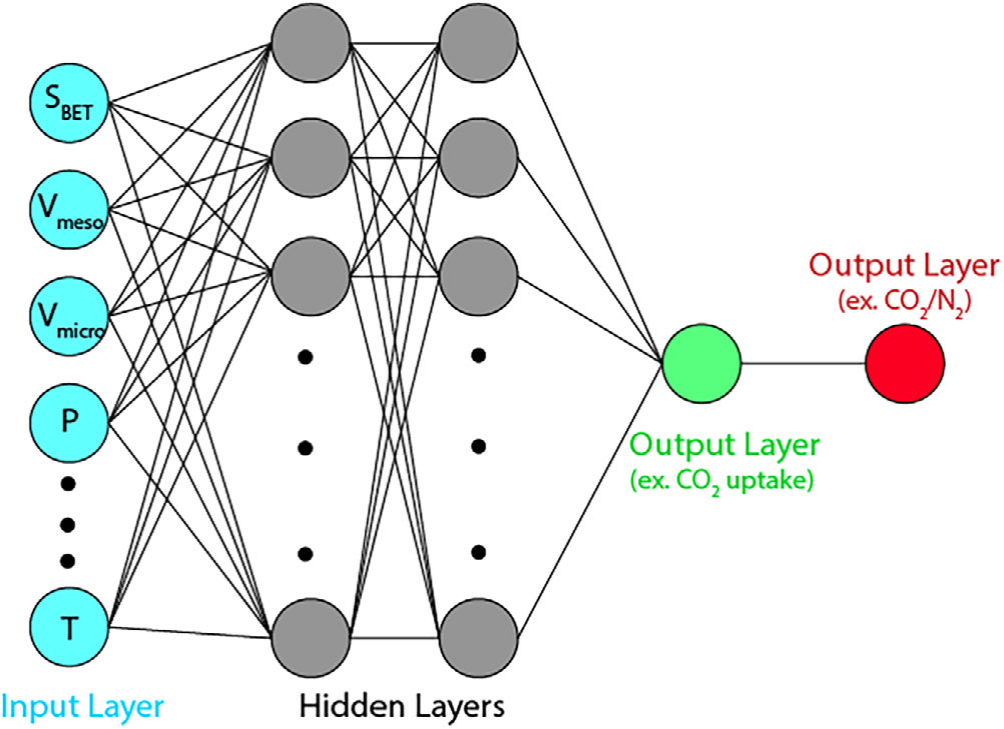
Neural network configuration and architecture for predicting CO_2_ uptake and selectivity in porous adsorbents.

Building on these advancements, Artificial Neural Networks (ANNs) have emerged as widely used machine learning tools for predicting properties and mass transfer in CO_2_ capture processes. Offering greater flexibility and often delivering more precise results than traditional numerical simulations, ANNs are instrumental in improving the efficiency and accuracy of carbon capture models.^[^ ^506^, ^507^, ^508^ ^]^ Briefly, in the case of porous adsorbents, the neural network configuration, as shown in **Figure** **16**, consists of an input layer, an output layer, and at least one hidden layer.^[^ ^509^ ^]^ The input layer comprises several parallel interconnected processing units, called neurons, where data related to CO_2_ uptake and selectivity in porous adsorbents is processed. In the hidden layer, each neuron is fully connected to neurons in neighboring layers, and the output of each neuron can be calculated using the following equation:^[^ ^510^, ^511^ ^]^

(40) $$y_{i}=f\left(\sum_{i=1}^{N}w_{ij}x_{i}+b_{j}\right)$$

where *w_ij_* represents the weight connecting the input and hidden layers, *j* is the hidden neuron, *x_i_* is the input, *b_j_* is the bias of the neuron in the hidden layer, and *f* is the activation function or transfer function of the neuron. Several activation or transfer functions are available, including the threshold function, sigmoid function, piecewise linear function, radial basis function, and Gaussian function.^[^ ^512^, ^513^, ^514^ ^]^ Among the different types of neural networks, the multilayer perceptron (MLP) is the most commonly used feedforward neural network, typically employing either sigmoid or hyperbolic activation functions in its hidden layers.^[^ ^515^, ^516^, ^517^ ^]^ Backpropagation neural networks (BPNN) and radial basis function neural networks (RBFNN) are commonly used ANN methods in CO_2_ capture processes. In backpropagation, as employed in PBNN, the algorithm iteratively minimizes the error between predicted and actual data by adjusting the weights and bias values, improving the model's accuracy over time.^[^ ^518^, ^519^, ^520^ ^]^

Although the ANN technique often produces strong correlations between inputs and outputs, it does not provide insights into the relationships between parameters. A promising alternative is the Adaptive Neuro‐Fuzzy Inference System (ANFIS), a hybrid system that combines the neural networks of ANN with the fuzzy logic of a Fuzzy Inference System. This integration aims to enhance the speed, fault tolerance, robustness, and adaptability of ANN, while maintaining the ability to learn from data and make accurate predictions.^[^ ^521^ ^]^ In this context, the ANFIS model has been employed to predict the CO_2_ diffusion coefficient.^[^ ^522^ ^]^ It has been demonstrated that the neuro‐fuzzy approach provides more accurate models, effectively supporting the correlation between variables in CO_2_ adsorption processes, particularly in amine‐based post‐combustion capture, which has been analyzed using statistical modeling. This approach has been tested on the CO_2_ adsorption capacity of aqueous sodium glycinates across various temperature and pressure ranges. The experimental data and ANFIS model exhibited excellent agreement, with an R‐squared value of 0.988 and an average relative error of 2.93%.^[^ ^523^ ^]^ ANFIS models have also been used to accurately estimate the CO_2_ loading capacity of amino acid saline solutions, despite the sensitivity of parameters like temperature uncertainty.^[^ ^524^ ^]^

Although the ANFIS method is well‐suited for modeling complex nonlinear relationships between parameters, formulating precise relationships between inputs and outputs, and generating fuzzy inference, it has certain drawbacks. A key limitation is that accurately defining the fuzzy membership functions requires expert knowledge of the problem domain. Additionally, the modeled correlations are not explicitly defined, which does not enhance the understanding of the underlying model. To address the limitations of ANN and ANFIS, the Piecewise Linear Artificial Neural Network (PWL‐ANN) approach presents a promising solution by modeling relationships within a carbon capture process system dataset. The PWL‐ANN algorithm combines the predictive power of ANN to create a reliable model while using a rule extraction method to provide explanations for the “black box” nature of traditional ANN models, offering better interpretability.^[^ ^510^ ^]^

While the PWL‐ANN approach addresses some of the limitations of traditional ANN models, the Support Vector Machine (SVM) algorithm offers another powerful solution for both classification and regression tasks. SVM goes beyond ANN by effectively managing challenges such as overfitting and poor generalization performance, providing a more robust model for complex datasets.^[^ ^525^, ^526^, ^527^, ^528^ ^]^ For regression, it works by finding the hyperplane that minimizes the distance between the actual and predicted values. In classification, the SVM identifies the hyperplane that maximally separates the two classes in the feature space, ensuring the greatest possible margin between them.^[^ ^529^, ^530^ ^]^ The SVM approach offers several advantages, including its ability to effectively avoid local optima and its capacity to handle challenges in selecting structural parameters.^[^ ^531^ ^]^ Commonly used kernel functions in SVM include linear, polynomial, radial basis function (RBF), and sigmoid.^[^ ^532^, ^533^ ^]^

Both the least squares version of support vector machine (LSSVM) and ANFIS models have demonstrated agreement in modeling the CO_2_ loading capacity of aqueous alkanolamine solutions as a function of system temperature, CO_2_ partial pressure, and amine concentration in the aqueous phase. However, ANFIS provided more accurate predictions without the need for complex thermodynamic principles and expressions.^[^ ^534^ ^]^

### 6.1 Leveraging AI for CO_2_ Capture Process Modeling

Several parameters significantly impact the efficiency of carbon capture processes, particularly the physical and chemical properties of adsorbents. Key properties include density, viscosity, diffusivity, heat capacity, reaction rate, and conductivity.^[^ ^535^, ^536^, ^537^ ^]^ Traditionally, these parameters are determined through laboratory experiments using costly instruments. Conducting such experiments requires expert knowledge, is time‐consuming, and often involves multiple iterations.^[^ ^511^, ^538^ ^]^ An alternative approach is to use numerical simulations developed based on empirical or semi‐empirical equations to estimate these properties.^[^ ^508^, ^539^, ^540^, ^541^ ^]^ However, this approach has several drawbacks, including: the inability to predict non‐linear relationships between parameters;^[^ ^542^ ^]^ the need for large datasets;^[^ ^543^ ^]^ time‐consuming computational processes; limited applicability of models to operational conditions beyond those they were developed for;^[^ ^544^ ^]^ the need for extensive function evaluations to validate models and simulations, and, complications arising from non‐ideal gas and liquid properties that can affect correlation calculations.^[^ ^545^ ^]^

Given these challenges, ML techniques have garnered significant attention for predicting the properties of CO_2_ capture processes, offering a more efficient and flexible solution.^[^ ^490^, ^546^, ^547^ ^]^

In case of CO_2_ absorption by ionic liquids, the input variables are pressure, temperature, and water/ionic liquid while the rate of CO_2_ uptake is the output. The widely used AAN models in this case are MLP and radial basis functions. It was found that the reformed MLP procedure using neurons align with increased transfer capability in the hidden layer outperforms the same radial basis function with the same number of neurons in the remove surfaces in terms of prediction efficiency.^[^ ^548^ ^]^ Although ANN techniques such as BPNN and RBFNN are effective in accurately estimating the physical properties of CO_2_ capture processes—such as density, thermal diffusivity, heat capacity, and viscosity of amine mixtures, with less than 1% deviation from actual results—the presence of impurities in amines must be considered, as they can negatively impact the accuracy of the predicted values.^[^ ^515^ ^]^ **Table** **9** summarizes the different algorithms used in AI‐assisted carbon capture research.

**Table 9:** Overview of algorithms applied in AI‐assisted carbon capture research.

AI algorithms	Operational parameters	Potential applications	Refs.
ML and DL	–	Reduction of carbon emissions in the healthcare industry	Das et al.^[^ ^549^ ^]^
ANN, CNN, DL, SVM, LSVM, RF, DT	–	Prediction of physical properties, monitoring CO_2_ plume migration and leakage, assessing mechanical stability, evaluation of carbon capture and storage success probability	Yao et al.^[^ ^550^ ^]^
ANFIS	Adsorption temperature, imidazole, Tetraethylenepentamine	Enhancing CO_2_ capture capacity	Olabi et al.^[^ ^551^ ^]^
ANN	CO_2_ uptake limits, CO_2_ production limits, carbon emission penalty coefficient, renewable power installed capacity	Analysis of the interactions between carbon, electricity and heat, minimizing process costs, reduction of required renewable power, study of the carbon emissions	Chen et al.^[^ ^552^ ^]^
ANN	Transport properties, operating temperature, mole fraction	Post‐combustion carbon capture	Nimmanterdwong et al.^[^ ^553^ ^]^
ANN	Liquid and gas flow rates, CO_2_ concentration in gas and liquid, nozzle diameter, amine concentration	Optimizing CO_2_ capture in spray columns	Di Caprio et al.^[^ ^554^ ^]^
HANFIS	Temperature, CO_2_ concentration, pH, nitrogen and phosphorous content	Prediction of CO_2_ fixation in microalgae	Kushwaha et al.^[^ ^555^ ^]^
ML	Hydrogen recovery factor, net present value	Optimizing CO_2_ storage	Kanaani et al.^[^ ^556^ ^]^
FL	Activation temperature and time, H_2_O/CO_2_ flow rate	Maximizing CO_2_ adsorption capacity	Nassef et al.^[^ ^557^ ^]^
ANN	Temperature difference between cell and absorption column, chlorine and copper shift	Energy optimization and performance for flue gas capture	Hasanzadeh et al.^[^ ^558^ ^]^
Machine Learning and Deep Learning: ML and DL Artificial Neural Network: ANN Convolutional Neural Network: CNN Deep Learning: DL Support Vector Machine: SVM Long Short‐Term Memory: LSTM		Random Forest: RF Decision Tree: DT Adaptive Neuro‐Fuzzy Inference System: ANFIS Hybrid Adaptive Neuro‐Fuzzy Inference System: HANFIS Fuzzy Logic: FL	

In addition to CO_2_ absorption processes, other AI‐driven areas of CO_2_ capture include gas adsorption,^[^ ^501^, ^559^ ^]^ energy management,^[^ ^560^ ^]^ and the analysis of the overall CO_2_ capture process.^[^ ^561^ ^]^ For instance, the pressure swing adsorption (PSA) method, widely used in industrial applications, has been studied for its role in predicting both adsorption and desorption processes.^[^ ^562^ ^]^ To construct an accurate model, several key parameters must be considered, including gas adsorption capacity, adsorbent selectivity, operating pressure, temperature, flow rate, and gas stream composition.^[^ ^563^ ^]^ For example, one study modeled PSA for CO_2_ capture, utilizing three different PSA cycles with two adsorbents simultaneously. The comparison between the model and experimental data demonstrated a good level of agreement between the partial differential algebraic equation and ANN models.^[^ ^564^ ^]^ ANN has also been successfully employed in the PSA purification process to predict the performance of separating gases such as H_2_ from gas mixtures like H_2_‐CO_2_‐CO.^[^ ^565^ ^]^

To develop ANNs for modeling the input and output parameters of amine‐based post‐combustion carbon capture, Sipocz et al.^[^ ^516^ ^]^ used training and validation data from rate‐based process simulations. The input parameters included inlet gas flow rate, inlet gas temperature, inlet gas composition, circulation rate, removal efficiency, and lean loading, while the output parameters were rich loading, mass flow rate of captured CO_2_, and heat duty of the process. The Scaled Conjugate Gradient (CG) and Levenberg–Marquardt (LM) algorithms were employed and compared for prediction accuracy. The results indicated that the LM algorithm produced more accurate predictions for the output parameters, making the predicted values suitable for pre‐designing a new power plant with CO_2_ capture capabilities. In another study, the efficiency of the technology was evaluated using a dynamic ANN‐based model to assess the recovery and utilization of CO_2_ from hydrogen tail gas. The results indicated that, instead of achieving 98.6% CO_2_ recovery, 91% could be used to offset costs, as predicted economically by the model.^[^ ^566^ ^]^

Numerous studies have been conducted on gas adsorption in porous structures like AC. One such study compared the accuracy of two‐dimensional cubic equations of state (EOS) with the ANN method for predicting pure and binary gas adsorption on AC. The findings revealed that the ANN model outperformed the EOS models, providing more accurate predictions, particularly for binary gas adsorption systems. This highlights the superior effectiveness of ANN in capturing and predicting complex adsorption phenomena.^[^ ^567^ ^]^

Recently, automated Deep Reinforcement Learning (DRL) has been applied to optimize processes for CO_2_ capture and storage. DRL has been used to optimize real‐time energy distribution and carbon capture processes in a Zero‐Carbon Multi‐Energy System (ZCMES). This system integrates Post‐Combustion Carbon Capture Systems (PCCS) with Direct‐Air Capture Systems (DACS) to effectively reduce CO_2_ emissions. The Soft Actor‐Critic (SAC) DRL agent has shown superior prediction capabilities, efficiently scheduling energy supply from renewable resources and conventional power systems while balancing energy consumption and CO_2_ capture efficiency. This real‐time scheduling approach outperforms traditional static optimization techniques by dynamically responding to fluctuations in energy demand, generation, and CO_2_ capture system performance.^[^ ^568^ ^]^

### 6.2 AI Applications in the Discovery of Biomass‐Derived AC Materials for CO_2_ Capture

Beyond process optimization, AI models using genetic algorithms can effectively search large databases of adsorbent materials, facilitating in silico discovery of high‐performing adsorbents with superior CO_2_ capacity and selectivity for pre‐combustion CO_2_ capture.^[^ ^569^ ^]^ Traditional development of biomass‐derived materials often relies on trial‐and‐error experiments, exploring various combinations of raw materials and conversion processes. Due to the complexity of this approach, most studies focus on a single or limited set of material design combinations.^[^ ^570^ ^]^ Tailoring biomass conversion for specific properties is challenging because of the complex relationships between feedstock, process, and resulting material properties, compounded by limited experimental data. To address these challenges, recent literature has employed ML in two key ways: i) predicting final material properties and product yield, and ii) predicting thermochemical conditions required to achieve targeted material performance, enabling reverse engineering to optimize production pathways for desired outcomes. In the case of biomass‐derived carbons, processing steps are relatively straightforward and do not require significant optimization.^[^ ^571^ ^]^ Thus, researchers have concentrated on synthesizing AC from biomass by considering parameters such as precursor type, pyrolysis temperature, and synthesis conditions.^[^ ^208^, ^572^ ^]^

Gas uptake prediction using ML is another emerging application for biomass‐derived carbons.^[^ ^573^, ^574^, ^575^ ^]^ ML models used to predict the adsorption capacity of bio‐based ACs incorporate key input variables, such as micropore volume, ultramicropore volume, mesopore volume, surface functional groups, specific surface area, and adsorption conditions. The objective is to identify enhanced CO_2_ adsorbents by utilizing material attributes like selectivity, efficiency, and heat of adsorption as performance indicators.^[^ ^470^ ^]^

Among ML models, Neural networks (NN), support vector machines (SVM), random forests (RF), and extreme gradient boosting (XGB) have been employed for this purpose. It is important to note that the performance of each model varies depending on the application. For instance, in some cases, random forests (RF) outperform support vector machines (SVM) and feedforward neural networks (FFNN),^[^ ^576^, ^577^, ^578^, ^579^, ^580^, ^581^ ^]^ while in others, SVM or neural networks (NN) deliver better results.^[^ ^581^, ^582^, ^583^, ^584^, ^585^ ^]^ Ensemble methods, such as RF and boosted regression trees (BRT), along with modified neural network models (such as ANFIS, Kriging, FCM‐ANN, and GRNN), have consistently shown superior performance on end‐use datasets.^[^ ^586^, ^587^ ^]^

However, despite the promising results, these models often face important limitations. As reflected in **Table** **10**, many studies rely on literature‐derived datasets that are heterogeneous in origin, which can introduce inconsistencies due to differing synthesis methods or measurement protocols. In addition, most models exclude essential parameters such as regeneration efficiency, adsorption kinetics, and long‐term material stability—limiting their practical applicability. Generalizability is also constrained, as many models are trained on narrow material classes (e.g., undoped porous carbons), and assumptions such as IAST or uptake‐ratio‐based selectivity can oversimplify real mixed‐gas behavior. Moreover, most models emphasize textural properties, with limited integration of chemical or electronic descriptors that may further explain performance.

**Table 10:** Summary of ML models used in porous carbon synthesis for CO_2_ capture

Dataset size	Key features	ML method	*R* ^2^	Key findings	Limitations	Refs.
527	S_BET_, V_tota_, V_micro_, N, O, H content, T, and P	GBDT, LGB, XGB	R^2^ = 0.84 (test), R^2^ = 0.98 (training)	GBDT model performed best for predicting CO_2_ adsorption with adsorption parameters (pressure and temperature) being the most important features.	Heterogeneous literature data may cause inconsistency; imputed values (TPV, MPV) add uncertainty. Model excludes kinetic/regeneration metrics due to data gaps.	Yuan et al.^[^ ^588^ ^]^
1774 (CH_4_), 1020 (CO_2_)	S_BET_, V_meso_, V_micro_, T, and P	RF, MLP	R^2^ = 0.99 (testing)	RF and MLP models accurately predicted CH_4_ uptake; S_BET_, V_micro_ were the most influential features. The MLP model was further extended to predict CO_2_/CH_4_ selectivity, highlighting the importance of mesopores in gas separation at high pressure.	Data sourced from heterogeneous literature may have inconsistencies. Limited to undoped porous carbons; may not generalize to other materials. IAST‐based selectivity assumes 50:50 gas mix. SSA imputation adds uncertainty.	Zhang et al.^[^ ^589^ ^]^
1000+	S_BET_, V_meso_, V_micro_, T, and P	DNN	R^2^ > 0.99	The DNN model accurately predicted CO_2_ adsorption across various porous carbons, demonstrating that micropores are crucial at low pressure, while mesopores play a larger role at higher pressures.	Relies solely on textural properties and excludes chemical functionalities (e.g., heteroatoms like N).	Zhang et al.^[^ ^575^ ^]^
6244	S_BET_, V_meso_, V_ultra‐micro_, T, and P	RF	R^2^ > 0.9	Textural properties had a more significant impact than chemical compositions on CO_2_ adsorption. At 1 bar, the ultra‐micropore volume showed a strong positive correlation with CO_2_ uptake (PCC = 0.715).	Data from heterogeneous sources may cause inconsistencies. Selectivity and kinetic/regeneration data were lacking. Some feature‐performance links remain weak due to limited molecular‐level descriptors.	Zhu et al.^[^ ^574^ ^]^
190	T, P, adsorption density	MLFNN	R^2^ > 0.99	MLFNN algorithm outperformed Sips and Langmuir isotherms in predicting CO_2_ adsorption on AC across various thermodynamic conditions.	Small literature‐derived dataset with potential experimental variability. Model is specific to AC and excludes kinetics, selectivity, regeneration, and uncertainty quantification.	Rostami et al.^[^ ^590^ ^]^
259	S_micropore_, V_micropore_, N/O functional groups	MLP‐NN, GA	R^2^ = 0.99	MLP‐NN model showed high accuracy in predicting the specific capacitance of N/O co‐doped AC electrodes in supercapacitors. S_micropore_ and pyrolytic‐N showed the highest impact on performance.	Study limited to 6 M KOH in a 3‐electrode system. Functional groups inferred from XPS data may vary in accuracy. Model lacks dynamic performance metrics like cycling stability and full‐cell scalability.	Rahimi et al.^[^ ^591^ ^]^
1288	Framework density, pore size, S_BET_, N content, atomic charge	RF, SVM, XGBoost, DNN	R^2^ > 0.97	The structural parameters, especially pore size and surface area, played a more significant role in CO_2_/CH_4_/N_2_ separation than nitrogen content. Nitrogen doping enhanced selectivity in ultra‐microporous structures.	Only microporous models were used; mesopores were excluded. Empirical potentials may miss atomistic details. Simulations at 298 K and 1 bar limit transferability. Results are based on simulated, not experimental, data.	Li et al.^[^ ^592^ ^]^
111 (N_2_), 679 (CO_2_), 291 (N_2_)	S_BET_, V_meso_, V_micro_	CNN	R^2^ > 0.99	CNN models predicted CO_2_ and N_2_ uptakes from 77 K N_2_ adsorption isotherms. Porous carbons with well‐separated micropores and mesopores showed the highest CO_2_/N_2_ selectivity up to 3000.	Only undoped carbons were modeled. Small dataset may limit generalizability. IAST assumes ideal behavior, which may not capture real mixed‐gas effects.	Wang et al.^[^ ^593^ ^]^
1138 (CO_2_), 314 (N_2_)	S_BET_, V_meso_, V_micro_, T, and P	DNN	R^2^ = 0.96	DNN accurately predicted CO_2_ and N_2_ uptake and CO_2_/N_2_ selectivity in porous carbons. The highest selectivity was achieved in regions where N_2_ uptake was disrupted by mesopores, enhancing CO_2_ selectivity.	Only non‐functionalized porous carbons were modeled; surface polarity and doping were excluded. Selectivity was based on uptake ratios, not IAST. Dataset lacked high‐selectivity cases, limiting generalizability.	Wang et al.^[^ ^594^ ^]^
421	Biomass precursors, activators, pyrolysis temperature, S_BET_, T, P	MLP, RBF	R^2^ > 0.99	MLP model showing superior performance in capturing the non‐linear relationships between biomass precursors and CO_2_ adsorption in AC.	Model is limited to ACs, assumes static conditions, excludes kinetics, long‐term performance, and uncertainty quantification.	Mashhadi et al.^[^ ^208^ ^]^
80	Activation temperature, S_BET_, V_meso_, V_micro_, current density	MLP, SVR	R^2^ = 0.98	MLP model provided the highest accuracy in predicting specific capacitance of mango seed husk‐derived AC for supercapacitor applications	Small dataset limits generalizability. Only one biomass type and 2 M NaOH electrolyte studied. Long‐term cycling, asymmetric setups, kinetics, and uncertainty quantification not addressed. Functional groups inferred, not fully quantified.	Wickram et al.^[^ ^595^ ^]^

RF: Random Forest; GBDT: Gradient Boosting Decision Trees; LGB: Light Gradient Boosting Machines; XGB: Extreme Gradient Boosting; MLP: Multi‐Layer Perceptron; MLFNN: Multi‐Layer Feed‐Forward Neural Network; SVM: Support Vector Machine; XGBoost: Extreme Gradient Boosting; GA: Genetic Algorithm; DNN: Deep Neural Network; CNN: Convolutional Neural Network; ANN: Artificial Neural Network; RBF: Radial Basis Function; SVR: Support Vector Regression; MLP‐NN: Multilayer Perceptron Neural Network

Unlike material and process design datasets, where support vector machines (SVM) with a radial basis function (RBF) kernel often excel, random forests (RF) can sometimes outperform SVM when applied to end‐use datasets. RF is particularly effective at predicting nonlinear relationships by utilizing multiple decision trees and averaging their predictions to reduce overfitting and minimize the influence of outliers. Additionally, RF can be combined with other ML techniques, such as ANN, to enhance its versatility. For instance, RF has shown strong performance in predicting the characterization of biochar and ACs derived from biomass.^[^ ^586^, ^587^ ^]^ Researchers have employed RF and gradient boosting regression (GBR) to predict biochar production, specific surface area, and total pore volume, while also optimizing parameters such as nitrogen content and production yield.^[^ ^596^, ^597^, ^598^ ^]^ Ma and colleagues applied RF to a CO_2_ adsorption dataset to study the influence of material properties on CO_2_ capture. Their findings showed that at low pressures (0–0.15 bar), nitrogen groups in porous carbon had the most significant effect on CO_2_ capture. However, as pressure increased to 0.15–1 bar, ultramicropores became the dominant factor influencing CO_2_ adsorption capacity. This underscores the pressure‐dependent roles of both chemical properties and pore structure in CO_2_ capture.^[^ ^599^ ^]^ Table 10 presents a summary of ML models used in porous carbon synthesis for CO_2_ capture.

Despite the advances in predicting biochar characteristics, most studies have focused more on biochar than AC due to the complexity of the AC activation process. However, with the increasing interest in applying ML to predict AC properties for industrial applications such as air purification and carbon capture, ML techniques, including ensemble methods, offer valuable solutions. Ensemble methods, known for their robustness to noise, tend to perform better on smaller datasets, making them more generalizable.^[^ ^600^, ^601^ ^]^ Similarly, neural networks have also shown improved performance when integrated with clustering methods, optimization techniques, and fuzzy logic, enabling them to effectively handle stochastic and small datasets. Additionally, ML models not only predict performance but also help identify key variables, enabling “what‐if” scenario analyses and parameter optimization for better decision‐making.^[^ ^571^, ^602^ ^]^

**Figure 17:**
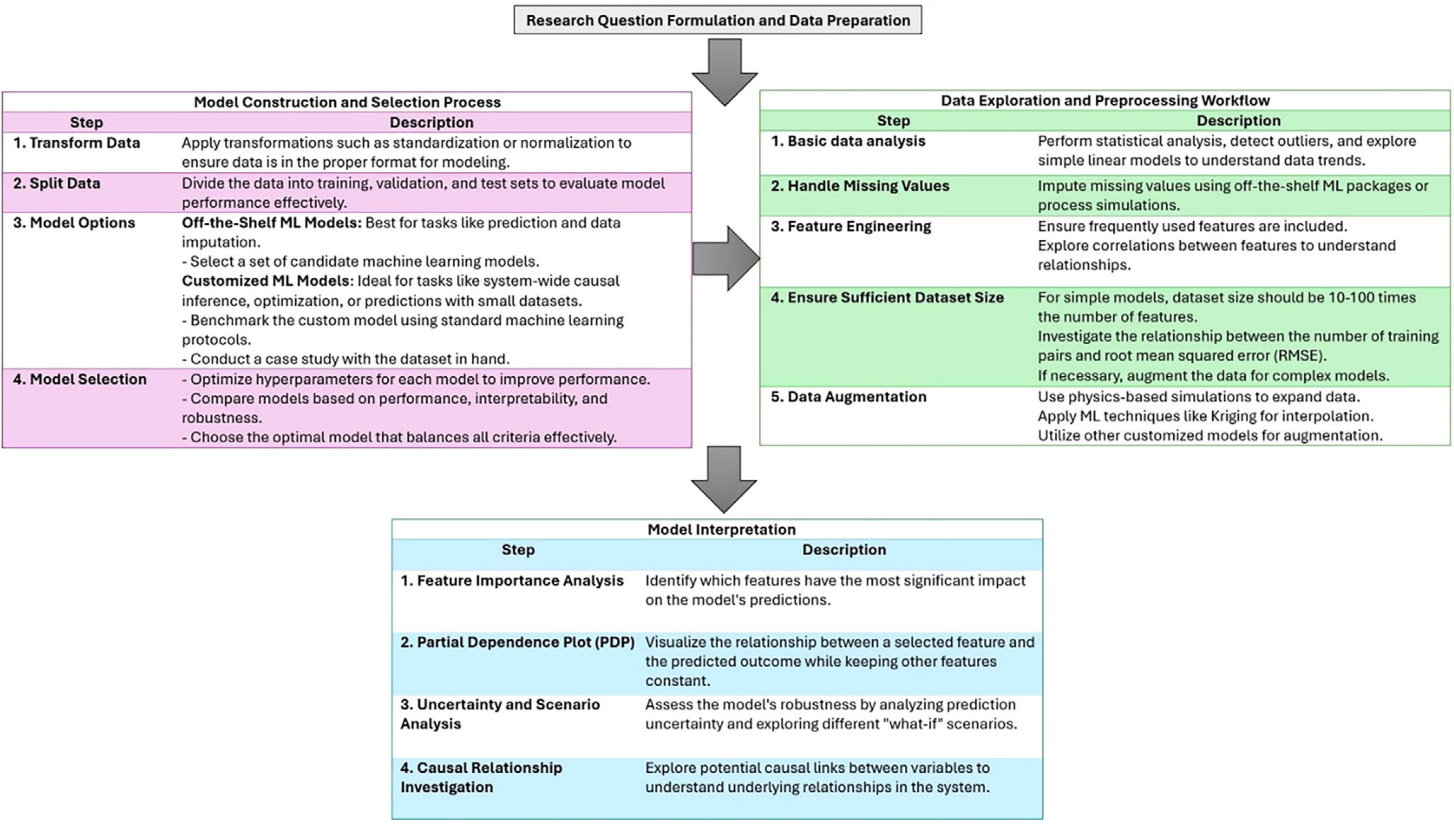
Recommended machine learning workflow for research formulation and data preparation in the knowledge discovery process.

**Figure** **17**, inspired from Wang et al.^[^ ^571^ ^]^ shows a recommended workflow for the knowledge discovery process in databases. In the initial stage of data exploration, commonly used parameters for feature engineering may include reaction parameters (e.g., temperature, time),^[^ ^603^, ^604^, ^605^, ^606^, ^607^, ^608^ ^]^ textural properties of biomass‐derived adsorbents (e.g., mesopore volume, ultramicropore volume, specific surface area),^[^ ^579^, ^580^, ^609^, ^610^ ^]^ feedstock properties (e.g., carbon, hydrogen, nitrogen content, lignin weight %, ash weight %),^[^ ^604^, ^611^, ^612^, ^613^, ^614^ ^]^ and environmental conditions (e.g., pH, temperature, time, gas pressure).^[^ ^574^, ^583^, ^615^ ^]^ Before building a model, both data quantity and quality need to be evaluated. In terms of data quantity, while it is challenging to estimate the required dataset size due to the diverse nature of the data and complexity of ML models, a general rule of thumb is that the sample size should be at least 10–100 times the number of features.^[^ ^616^, ^617^, ^618^, ^619^ ^]^ If the dataset needs to be expanded, methods such as interpolation, simulation, or Kriging, along with customized ML techniques, can be used. For deep neural networks, researchers have shown that using more than 15000 training pairs significantly improves model performance, indicated by reduced mean squared error.^[^ ^510^, ^571^ ^]^

When constructing a model, using off‐the‐shelf ML models is often a good approach for projects focused on predicting specific outcomes. It is recommended to compare the performance of both simple and complex models to guide algorithm selection. For model selection, candidate models should be optimized and evaluated against one another. In cases where system‐wide decision‐making is required, developing customized, physics‐informed ML models is advisable to ensure robustness and real‐world applicability. For new algorithms, it is essential to benchmark their performance using standard ML protocols, such as cross‐validation, and compare outputs and computation times with results from widely‐used ML packages.^[^ ^503^ ^]^

Once an ML model is constructed, various analyses can be applied to interpret its results. For biomass‐derived adsorbents, techniques such as feature importance analysis, partial dependence plots (input‐output relationship analysis), and SHAP (SHapley Additive exPlanations) have been commonly used. Uncertainty and scenario analysis are also valuable for supporting sustainability decision‐making and optimizing the application of biomass‐derived adsorbents.^[^ ^620^, ^621^ ^]^ Additionally, ML models enable the prediction of “what‐if” scenarios and simulations to guide decision‐making. Investigating causal relationships through ML provides further insights into process and material optimization, enhancing our fundamental understanding of bio‐derived materials.^[^ ^571^ ^]^

Overall, the integration of AI in carbon capture, whether for process optimization or materials discovery, has yielded highly promising results, particularly in estimating critical parameters and identifying high‐performance sorbents. For AI‐enabled carbon capture to reach its full potential, close collaboration between engineers and AI experts is crucial. These partnerships can drive significant advancements, including the optimization of CO_2_ capture processes, reduction of installation and operational costs, increasing the efficiency of carbon capture plans, development of novel sorbent materials with enhanced capacity and selectivity, and ultimately, the creation of more efficient and innovative CO_2_ capture facility designs.

## 7 Conclusions and Future Perspectives

Over the past decades, the field of CO_2_ capture has witnessed remarkable progress. While conventional liquid amine‐based sorbents have been utilized for more than five decades, their limitations—such as high regeneration costs, corrosive properties, and amine loss—have driven extensive research into alternative materials. Among these, porous solid materials, including AC, have gained significant attention due to their cost‐effectiveness, high specific surface area, and well‐developed pore structure, making them widely applicable in adsorption technologies.

Building on these advancements, the synthesis of bio‐based AC offers a promising pathway to further mitigate CO_2_ emissions while addressing environmental and sustainability challenges. This review emphasizes the importance of biomass‐derived precursors in producing AC with tailored structural and surface properties optimized for carbon capture. Techniques such as nitrogen doping and metal impregnation have shown potential in enhancing the basicity and adsorption performance of AC, while both physical and chemical activation methods allow for precise control over pore structure and surface chemistry. Furthermore, innovative strategies for designing carbon‐based materials capable of capturing CO_2_ in humid environments—such as hydrophobic modifications, the incorporation of polar functional groups, water‐facilitated CO_2_ adsorption, and amine functionalization—have been explored.

Additionally, careful consideration is necessary during modifications to avoid pore blockage, which can hinder CO_2_ diffusion within the porous structure and reduce the number of active adsorption sites. Modifications must be optimized to suit the specific process for which the sorbent is designed. It is essential to precisely control treatment methods to ensure that the introduced functional groups enhance CO_2_ capture, as not all functional groups contribute positively. Furthermore, special attention should be given to the regeneration of surface‐functionalized activated porous carbons to mitigate issues related to toxicity and the potential corrosion of downstream equipment.

The integration of AI‐driven tools into material discovery and process modeling has significantly advanced the development of bio‐based adsorbents, enabling more efficient optimization and performance prediction. This progress is paving the way for next‐generation carbon capture solutions that are both effective and adaptable. Beyond material discovery, AI algorithms are also instrumental in monitoring the performance of CO_2_ capture systems, allowing for real‐time adjustments to maximize efficiency and prevent costly disruptions, ensuring the long‐term operational reliability of these technologies.

However, the application of AI in carbon capture is not without challenges. For instance, a study by the University of Massachusetts revealed that training and searching specific neural network architectures can emit ≈626000 pounds of CO_2_—nearly five times the lifetime emissions of an average American car, including its manufacturing. To mitigate this, improving the computational efficiency of AI systems is essential. Advances in automated AI training and optimization methods have the potential to dramatically reduce these emissions, in some cases lowering them to the low triple digits.^[^ ^622^ ^]^ By addressing these challenges, AI can continue to drive innovations in carbon capture while minimizing its environmental footprint.

The use of bio‐based AC for CO_2_ capture holds significant promise, yet several challenges and complexities must be addressed to optimize its effectiveness. Achieving high selectivity for CO_2_ over competing gases and water vapor is critical, requiring precise optimization of the capture process. Enhancing reaction kinetics, often through the application of catalytic methods, and employing viscosity‐reducing agents can facilitate improved gas flow and sorbent performance by mitigating the effects of CO_2_ adduct formation. Additionally, ensuring the stability and durability of bio‐based AC is vital for sustained operation in long‐term capture applications. For example, amine‐based sorbents can experience degradation over repeated reaction cycles due to temperature variations or contamination by acidic gases like SO_x_ and NO_x_, underscoring the need for robust material design and process control.^[^ ^623^ ^]^

Despite their significant potential, many of these materials are still in the early stages of development for commercialization. Among the various options available, post‐combustion CO_2_ capture emerges as the most feasible for large‐scale implementation, given its minimal modification requirements and the ease of retrofitting capture units into existing plants. However, the large‐scale production of AC from biomass remains underexplored. Addressing this gap requires a comprehensive evaluation of production costs, including the expenses associated with biomass precursors, additional chemicals, and the necessary experimental infrastructure. Scaling up AC production must not only achieve high efficiency in CO_2_ capture but also align with environmental sustainability principles. Additionally, future research should prioritize the effective management and disposal of by‐products and waste generated during production, with a strong focus on pollution control strategies to ensure minimal environmental impact.

In parallel, the reliance on biomass feedstock for AC production introduces additional challenges, as biomass is also in demand for other critical applications, such as food and energy production. This competition limits the sustainable availability of raw materials and is further exacerbated in regions facing water scarcity. Consequently, the use of biomass for carbon capture faces several key obstacles, including: variability and inconsistency in feedstock and biomass composition, leading to challenges in scalability; traditional synthetic adsorbents often have a cost advantage over biomass‐based alternatives; industries may hesitate to adopt biomass sorbents and transition from established carbon capture technologies, particularly as bio‐based sorbents remain in the developmental stage.

On another note, it is essential to consistently evaluate the environmental impact of biomass cultivation and mitigate any adverse effects. Comprehensive environmental assessments and the adoption of sustainable practices are essential for the effective integration of biomass‐based adsorbents into CO_2_ capture technologies. Additionally, promoting large‐scale reforestation and forest planting initiatives is crucial for building a sustainable future while securing a dependable supply of biomass.

However, the environmental benefits of biomass‐based materials must be balanced against the energy consumption and economic challenges associated with their production. Developing cost‐effective, high‐performance solutions remains a critical goal in the field. The production of high‐performance porous carbon materials often involves energy‐intensive processes, such as high‐temperature carbonization and activation, which can lead to elevated preparation costs. These expenses may restrict the competitiveness of such materials in large‐scale industrial applications, highlighting the need for innovative approaches to reduce energy demands and production costs while maintaining sustainability. To reduce preparation costs and enhance production efficiency for industrial applications, the development of more efficient and eco‐friendly methods for producing porous carbon materials is essential. Approaches such as green synthesis techniques and rapid carbonization processes are expected to play a pivotal role in achieving these goals.

To overcome these challenges, future research and development efforts should focus on enhancing production efficiency, lowering energy consumption costs, ensuring consistent production quality, optimizing the properties and performance of bio‐based materials, and minimizing their environmental footprint to position them as a competitive and sustainable alternative to traditional solutions.

## Conflict of Interest

The authors declare no conflict of interest.
